# Autonomous Multi‐Step and Multi‐Objective Optimization Facilitated by Real‐Time Process Analytics

**DOI:** 10.1002/advs.202105547

**Published:** 2022-02-01

**Authors:** Peter Sagmeister, Florian F. Ort, Clemens E. Jusner, Dominique Hebrault, Thomas Tampone, Frederic G. Buono, Jason D. Williams, C. Oliver Kappe

**Affiliations:** ^1^ Institute of Chemistry University of Graz NAWI Graz, Heinrichstrasse 28 Graz 8010 Austria; ^2^ Center for Continuous Flow Synthesis and Processing (CCFLOW) Research Center Pharmaceutical Engineering GmbH (RCPE) Inffeldgasse 13 Graz 8010 Austria; ^3^ Chemical Development US Boehringer Ingelheim Pharmaceuticals, Inc. 900 Ridgebury Road Ridgefield Connecticut 06877 USA

**Keywords:** chemometrics, data‐rich experimentation, flow chemistry, machine learning, organic synthesis, process analytical technology, self‐optimization

## Abstract

Autonomous flow reactors are becoming increasingly utilized in the synthesis of organic compounds, yet the complexity of the chemical reactions and analytical methods remains limited. The development of a modular platform which uses rapid flow NMR and FTIR measurements, combined with chemometric modeling, is presented for efficient and timely analysis of reaction outcomes. This platform is tested with a four variable single‐step reaction (nucleophilic aromatic substitution), to determine the most effective optimization methodology. The self‐optimization approach with minimal background knowledge proves to provide the optimal reaction parameters within the shortest operational time. The chosen approach is then applied to a seven variable two‐step optimization problem (imine formation and cyclization), for the synthesis of the active pharmaceutical ingredient edaravone. Despite the exponentially increased complexity of this optimization problem, the platform achieves excellent results in a relatively small number of iterations, leading to >95% solution yield of the intermediate and up to 5.42 kg L^−1^ h^−1^ space‐time yield for this pharmaceutically relevant product.

## Graphical abstract

A modular platform combines rapid spectroscopic analytics and chemometric processing to facilitate autonomous optimization of organic synthesis in flow. This enables increased complexity of the optimization problem. As a demonstration, a two‐step reaction is tackled: synthesis of the active pharmaceutical ingredient, edaravone. This seven variable, three objective optimization provides conditions for a mass‐efficient and highly productive process.

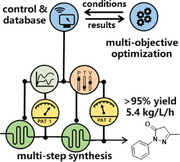

## 1 Introduction

The digitalization of synthetic chemistry has seen significant interest in recent years, with a focus on data‐rich environments^[^ ^1^ ^]^ and automated (closed‐loop) experimentation.^[^ ^2^ ^]^ Continuous flow processing provides a perfect arena to test and implement this automation, due to the continuous nature of experiments and the ease of automating unit operations, such as delivering reagents via pumps.^[^ ^3^ ^]^ Accordingly, a wide range of self‐optimizing flow reactor systems has been disclosed over the past ten years, often focusing on synthetic organic chemistry applications,^[^ ^4^ ^]^ but also polymer synthesis^[^ ^5^ ^]^ and materials science.^[^ ^6^ ^]^ These applications highlight the exciting prospects when the benefits of continuous flow processing, such as improved heat‐ and mass transfer,^[^ ^7^ ^]^ are combined with its enhanced automation potential, to rapidly explore chemical space and optimize processes.

**Figure 1:**
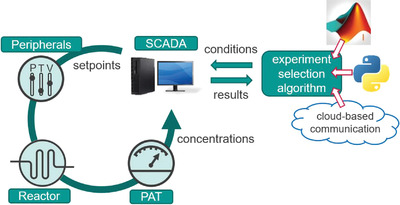
Simplified schematic depiction of the present self‐optimizing reactor platform. Input variables are delivered to the central SCADA system in a universal format, by an experiment selection algorithm of the operator's choice. The SCADA system calculates the required set points for pumps and other reactor peripherals. After the reaction takes place, spectra are measured inline using PAT, then processed in real‐time using chemometric models to determine reactant concentrations. The concentrations are then further processed by the SCADA system to provide the desired objective values, then returned to the decision algorithm for inclusion to the current model and selection of the next reaction conditions.

A key aspect of automated reactor platforms is connectivity and compatibility between hardware and software. This can be accomplished in a flexible and straightforward way through the use of a central supervisory control and data acquisition (SCADA) system.^[^ ^8^ ^]^ Using a universal communication protocol, such as open platform communication unified architecture (OPC UA),^[^ ^9^ ^]^ all data can be logged and monitored, whilst the hardware can also be controlled. This system bridges the gap between an experiment selection/optimization algorithm and the laboratory equipment required to operate the experiments. In turn, this enables access to a wealth of already developed optimization algorithms,^[^ ^2c^ ^]^ which require only the addition of a simple functionality (e.g., read/write to a text file) for back‐and‐forth communication between the two (**Figure** **1**). This mode of operation also provides the possibility of cloud‐based optimization. Therein, the experiment selection algorithm could be based anywhere in the world, whilst making use of the central reactor hardware setup.^[^ ^10^ ^]^

Arguably the most challenging detail of closed‐loop optimization is the analysis of output chemical composition using process analytical technology (PAT),^[^ ^11^ ^]^ which provides the corresponding responses for each set of input variables. The majority of self‐optimizing reactors use gas or liquid chromatographic methods (GC^[^ ^12^ ^]^ or HPLC/UHPLC), providing a high level of accuracy due to the separation of species prior to detection. However, this separation also requires a significant amount of time, often leading to analysis times in the range of several minutes. Therefore, these methods are often limited by the time required for analysis and the results will be based on only a single measurement, which could lead to errors in the case of an inhomogeneous sample or sample injection issues.

Conversely, spectroscopic methods have more recently come to the fore in the field of organic synthesis in flow. These instruments have the capability of measuring in‐ or online (analyzing the whole reaction stream or in a bypass) and have significantly faster measurement times, in the range of 10–15 s per data point. Measuring data points more frequently allows more efficient reaction operation, by automated steady‐state detection and time‐averaged data. The combination of these techniques with chemometric modeling can provide accurate quantification of component concentrations, even in cases of complex mixtures with overlapping components.^[^ ^13^ ^]^ Numerous examples of self‐optimization using spectroscopic methods (e.g., FTIR, NMR, UV/vis) exist,^[^ ^14^ ^]^ but their combination with chemometric modeling approaches will provide a powerful real‐time evaluation method for closed‐loop applications.

The current state‐of‐the‐art applications of autonomous optimization are based only on a single reaction step, with a single analysis point. Moreover, the number of optimizable variables (e.g., reaction temperature, concentration, reagent loadings) is most frequently between 2 and 4. To increase the applicability and utility of autonomous optimization in flow reactors, the incorporation of additional complexity would be highly desirable. Increasing the number of optimizable variables exponentially expands the accessible experimental space, but is a necessary step to further advance the field. The use of multi‐objective optimization algorithms also provides an opportunity to implement additional measurement points, further increasing the coverage and applicability of experimental results.

Herein, we report the development and operation of a modular reaction platform, which can integrate multiple PAT tools with chemometric processing. The platform is flexible, allowing straightforward communication with numerous reaction selection/optimization algorithms. To demonstrate the high level of functionality and flexibility, we carried out the multi‐objective self‐optimization of a two‐step process for the synthesis of the amyloid lateral sclerosis drug edaravone, with seven optimizable variables and three competing optimization objectives.

## 2 Results and Discussion

### 2.1 Reactor Concept and Setup

The present reactor setup is based on a modular microreactor system (Ehrfeld, MMRS), incorporating thermostated (Huber, Unistat 240) reactor units, as well as temperature and pressure sensors. Syringe pumps (HiTec Zang, SyrDos 2) or HPLC pumps (Knauer, Azura) were used to deliver reagent and solvent streams to the reactor itself. All sensors, peripherals, and PAT tools were controlled by a central SCADA system (Evon, XAMControl), via OPC UA.^[^ ^13d^ ^]^

In contrast to many other reported self‐optimization systems, this platform was designed to make optimal use of frequent concentration measurements, provided in real‐time from the spectroscopic PAT methods.^[^ ^14^ ^]^ First, a finite impulse response (FIR) filter^[^ ^15^ ^]^ was incorporated, which was applied every time a new measurement was received. The filter first corrected for outliers, then averaged the past ten concentration measurements in a time‐weighted fashion (i.e., most recent results have the highest weighting). This served to minimize noise in the data stream and to ensure that the objective values were as accurate as possible. Furthermore, there is a clear advantage versus, single measurement‐based approaches, which could be skewed by momentary inhomogeneities or sample injection/measurement errors.

Representative results for each set of conditions can only be assessed once the reactor is in steady state. A common approach to assigning steady state is to simply wait for a set number of residence times (often 3) to pass through the reactor, yet this is time (and material) consuming and may not always be necessary. In addition to this approach, we applied a gradient‐based steady state check, using the data recorded by the PAT instruments. If the (absolute) gradient between two filtered concentration measurements was lower than 5 mM, the waiting time for steady state could be reduced to 1.5 residence times. Although a seemingly minor alteration, this check served to significantly reduce the time for reaction iterations in many cases—often by 10–15 min over a two‐step sequence.

### 2.2 Single Step Self‐Optimization

**Figure 2:**
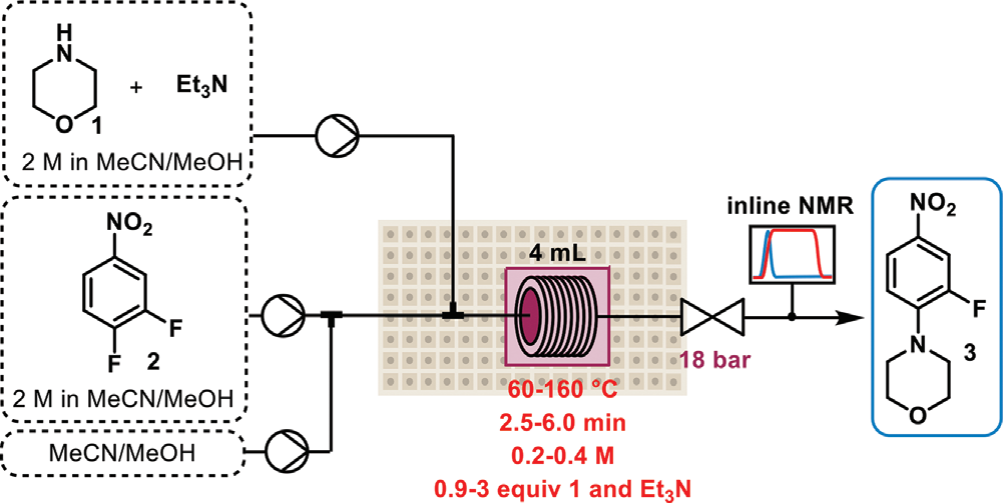
Simplified reaction scheme of the model single step reaction used to test the self‐optimization platform—synthesis of linezolid intermediate **3** by S_N_Ar reaction of morpholine **1** with 3,4‐difluoronitrobenzene **2**.

To examine the platform's capabilities, a single step optimization was first carried out using the nucleophilic aromatic substitution (S_N_Ar) reaction between morpholine (**1**) and 3,4‐difluoronitrobenzene (**2**) (**Figure** **2**). Despite its simplicity, this reaction makes up the first step in the synthesis of linezolid, an antibiotic drug on the WHO list of essential medicines.^[^ ^16^ ^]^ This was performed in a 4 mL tubular reactor, with four optimizable variables (and their corresponding upper and lower boundaries): 1) concentration (0.2–0.4 M); 2) temperature (60–160 °C); 3) equivalents of morpholine and triethylamine base (0.9–3.0 equiv); 4) residence time (2.5–6 min).

After leaving the reactor and passing through the back pressure regulator (BPR, set to 18 bar to prevent solvent evaporation at high reaction temperature), the reaction mixture was analyzed by inline NMR (Magritek, Spinsolve Ultra 43 MHz). The starting material **2** and product **3** could be quantified using an indirect hard model (IHM),^[^ ^17^ ^]^ constructed using chemometrics software (S‐PACT, PEAXACT). The model was calibrated using four different mixture concentrations, resulting in a root mean square error of cross validation (RMSE_CV_) of 12.2 mM and 4.8 mM for starting material **2** and product **3**, respectively. This error represents <5% of the expected reaction concentration in almost all cases.

A broad range of optimization algorithms for chemical systems have been investigated in previous work,^[^ ^18^ ^]^ but the most commonly used approaches are: simplex‐based (e.g., Nelder‐Mead),^[^ ^19^ ^]^ gradient‐independent methods (e.g., SNOBFIT)^[^ ^14d^ ^]^ or Bayesian approaches (e.g., TSEMO).^[^ ^20^ ^]^ The Thompson sampling efficient multi‐objective (TSEMO) optimization algorithm, recently introduced by Bourne, Lapkin, and coworkers,^[^ ^20^ ^]^ operates by building Gaussian process (GP) surrogate models at each iteration, then selecting an experimental point from this model, which is expected to provide the greatest increase in well‐described model space (known as hypervolume improvement). The resulting set of optimal conditions is generally a trade‐off between different objectives, so would allow an operator to tune the reaction conditions toward whichever objective is most important at that time.^[^ ^20^ ^]^

In order to begin the optimization, the original algorithm creates a Latin hypercube^[^ ^21^ ^]^ (semi‐random, but well‐spaced selection) of conditions, to scan the parameter space. This serves to provide a good initial model for selection of conditions, but may also be unnecessary for an effective self‐optimization experiment (recommended five experiments per optimizable variable). Numerous other types of initial data sets for self‐optimization exist, such as performing an automated DoE study (e.g., two‐level full factorial design—16 experiments and one center point for this four‐variable setup).^[^ ^22^ ^]^ Finally, it may be resource‐effective to simply provide the optimization algorithm with a very minimal amount of data, such as one central set of conditions.

**Figure 3:**
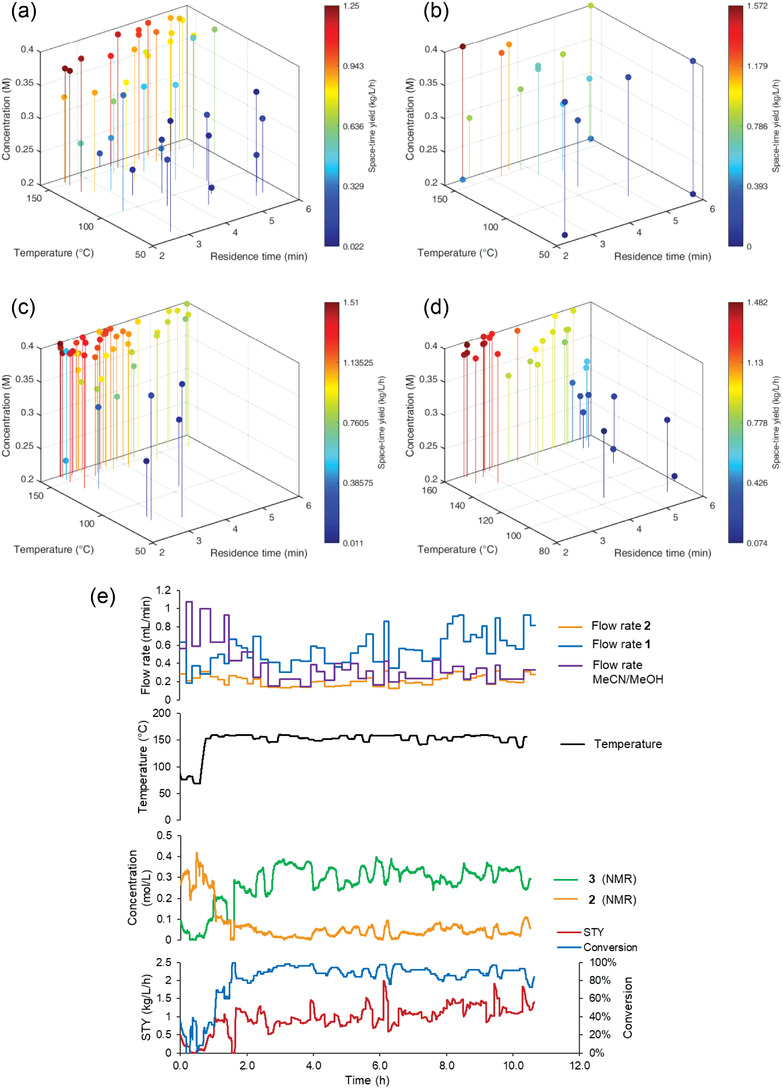
Plots showing the space‐time yield achieved as a function of concentration, residence time, and temperature, in self‐optimization experiments using different starting points: a) Latin hypercube; b) Full factorial DoE; c) Center‐point only (1); d) Center‐point only (2); e) Data summary from center‐point only (2) experiment (Table 1 Entry 5, Figure 3d), showing flow rate and temperature set points as well as NMR measured concentrations and both objectives over time.

We endeavored to determine the most effective starting point for this type of system, by running self‐optimization experiments using numerous different start points. For this comparison, the two objectives: 1) conversion (quantity of starting material **2** consumed; %); 2) space‐time yield (reaction throughput divided by reactor volume; kg l^−1^ h^−1^) were selected. The performance of each approach in finding optimal solutions within ≈34 iterations is compared below (**Table** **1**). The maximum space‐time yield, and the number of experiments taken to reach that value, are compared, as well as the number of iterations taken to reach >95% conversion. A graphical comparison of each of these experiments is shown in **Figure** **3**, depicting the space‐time yield achieved as a function of temperature, concentration, and residence time. Full details of each optimization experiment are available in the Supporting Information.

**Table 1:** Comparison of optimization experiments using different starting points for the optimization algorithm initial model

Entry	Starting point type	Number of iterations prior to self‐optimization	Max space‐time yield [kg L^−1^ h^−1^] (total iterations taken)	Iterations taken to reach >95% conversion
1	Latin hypercube	20	1.25 (24)	28
2	Full factorial DoE	17	1.57 (16)	17
3	Restricted full factorial DoE	17	1.23 (27)	29
4	Center‐point only (1)	1	1.29 (13) 1.43 (28)	17
5	Center‐point only (2)	1	1.27 (22) 1.48 (23)	14
6	Center‐point only (3‐Objective optimization)	1	1.26 (33)	17

Although the Latin hypercube approach (Entry 1, Figure 3a) clearly benefited from the initial knowledge provided to the optimization algorithm, 4 and 8 additional experiments were still required to reach the maximum space‐time yield and conversion, respectively. This implies that much of the initial data was likely in areas away from the optimal reaction conditions and, therefore, less useful to the iterative process models. It should be noted, however, that a reduced number of Latin hypercube iterations may likely be incorporated without much effect on the quality of the starting self‐optimization model. This is particularly true for relatively simple reaction systems, such as this one, but will require additional experiments as the optimization problem increases in complexity.

Another approach to provide a significant amount of information to the optimization algorithm would be the use of a full‐factorial DoE design, which maps out the results at maximum and minimum parameter values, as well as the center of all parameters (Entry 2, Figure 3b). This approach is often used to build reaction models with quantified variable effects (as well as variable interaction effects), so it should also provide a good basis for self‐optimization experiments.^[^ ^22^ ^]^ A high space‐time yield (1.57 kg L^−1^ h^−1^), as well as >95% conversion, was reached within the first 17 DoE experiments (albeit in two separate iterations), which proved to be advantageous. However, for a more complex reaction system, it is unlikely that such solutions would lie on the initial DoE corner points. Furthermore, when a more complex system with more input variables is examined, the number of experiments in a full factorial design increases exponentially (2*^n^* experiments, where *n* = number of variables). In such cases, a reduced factorial design (e.g., 2*^n^* ^−1^ or 2*^n^* ^−2^) could be applied.

To test whether this approach is still effective if the DoE design space is different from the self‐optimization design space, a second full‐factorial DoE experiment was performed (Entry 3). However, in this case, the DoE was performed in a reduced area of the parameter space, with the hypothesis that it should still be possible to approximate the effects of each optimization variable, despite not covering the exact same experimental space. As could be expected, this approach took significantly longer to reach optimal conditions (29 versus 17 iterations to reach >95% conversion), implying that there is a significant penalty to the self‐optimization efficiency.

Building the initial GP model was attempted with only a single experimental point, prior to optimization experiments (the algorithm requires two data points, but in this case an identical set point was used twice). To ensure that the optimization pathway was reproducible, this was performed twice (Entry 4 and Entry 5, Figure 3c and Figure 3d). Although the initial experimental set points were somewhat divergent, the same patterns were found within a small number of experiments. In both attempts, a similar space‐time yield maximum (1.2–1.3 kg L^−1^ h^−1^) was achieved in as little as 13 total iterations. Furthermore, the increased number of available optimization iterations actually allowed higher maximum space‐time yields of 1.43 and 1.48 kg L^−1^ h^−1^ to be found. In addition, the total number of iterations required to reach >95% conversion was minimal (17 and 14 versus 28 with Latin hypercube).

Finally, an additional experiment was performed, in which a third optimization objective, minimized E‐factor,^[^ ^23^ ^]^ was added (Entry 6). This is simply defined as the mass of waste generated in the reaction, divided by the mass of product formed, serving to optimize toward a process which makes optimal use of the input materials. This was calculated continuously, based on the input variables and the last measured product yield (see Supporting Information section 5.2 for details of the calculation). Despite the addition of this third objective, >95% conversion was reached in only 17 iterations. Since the space‐time yield and the E‐factor are often opposing objectives (i.e., space‐time yield benefits from incomplete conversion and excess reagents), it took 33 iterations to reach the optimum 1.48 kg L^−1^ h^−1^ space‐time yield. Despite the requirement for additional iterations, the optimization was effective for all three objectives, also achieving low E‐factor values below 9 (including solvent usage).

In conclusion, it appears that in this case, the most efficient method of operating this algorithm is by simply starting the optimization algorithm as early as possible, without expending resources on pre‐iterations to enhance the initial model quality. One important consideration, though, is the amount of the design space covered by such initial data. When complex local and global optima are present, the implementation of a design space search prior to optimization may prevent the optimization falling into a local optimum. The quantity of material consumed in these experiments amounts to roughly 3.1 g h^−1^ of starting material **2**, which further highlights the benefit of minimizing experimental iterations.

Due to the iterative construction of GP models in this optimization algorithm, additional process knowledge is gained, when compared to gradient‐based and other “black box” optimization methods. A qualitative measure of the parameter's influence on the optimization objective, *θ*, is provided after each iteration. The lower its value, the higher impact this parameter has on the outcome.^[^ ^20a^ ^]^ This revealed that in the S_N_Ar reaction, the reaction temperature had the greatest influence on both conversion and space‐time yield, followed by the equivalents of morpholine. Pleasingly, all of the experiments listed in Table 1 provided similar arrays of *θ* values, providing further confidence in this methodology (see Supporting Information for values arising from each optimization experiment).

### 2.3 Multi‐Step Self‐Optimization

To further test the limits of the optimization platform, a telescoped flow process was examined. Previous applications of self‐optimizing reactors have been limited to only a single chemical step, with one point of analysis. Due to the rapid data acquisition employed here, it was thought possible to optimize two chemical reaction steps simultaneously, taking advantage of the multi‐objective optimization possibilities to take two measuring points into account. The number of optimizable variables in past demonstrations has typically been 3 or 4 (5 in a small number of cases).^[^ ^10b^ ^]^ The complexity of the optimization problem increases exponentially with the number of variables, therefore, we attempted to push this platform to its limits with 7 optimizable variables.

**Figure 4:**
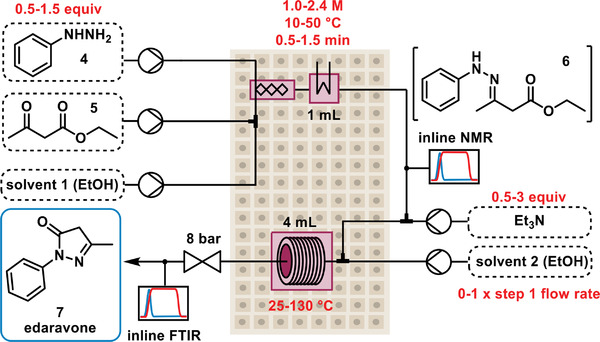
Simplified reaction scheme of the two‐step reaction—synthesis of edaravone **7** by condensation of hydrazine **4** and ketoester **5**, followed by cyclization of imine intermediate **6**. Upper and lower bounds of optimizable variables are shown in red.

The chemistry to be optimized was the synthesis of the active pharmaceutical ingredient edaravone, a treatment for amyloid lateral sclerosis and for stroke recovery.^[^ ^24^ ^]^ This can be achieved in two reaction steps: first a condensation of phenylhydrazine **4** with ethyl acetoacetate **5** to form the imine intermediate **6**, followed by a base‐mediated cyclization (**Figure** **4**). A recent publication detailed the need to separate the imine formation and cyclization reaction steps, to minimize the formation of undesired side products.^[^ ^25^ ^]^

Analysis of the reaction took place after the first and second reaction steps, using inline NMR (Magritek, Spinsolve Ultra 43 MHz) and inline FTIR (Mettler Toledo, ReactIR 15, SiComp flow cell), respectively. The use of high temperatures for the cyclization necessitated elevated pressure to prevent solvent vaporization. As a result, the inline NMR had to be operated using polymer tubing in place of the glass flow‐through cell, which lowered the resolution of the measurements. The IHM calibrated for the three species at this point (**4**, **5,** and **6**) had higher validation errors than in the previous method (15.4, 17.9, and 74.4 mM, respectively), but due to the high concentrations in this step (1.0–2.4 M), these values were deemed to be acceptable (e.g., 3.1% error for **6** at 2.4 M concentration). The FTIR used a partial least squares (PLS) model, which also provided suitably low errors, when compared with the reaction concentrations (69.3, 36.7, 98.3, and 26.6 mM for **4**, **5**, **6,** and **7**, respectively).

In this two‐step sequence, seven optimizable variables were set: 1) equivalents of phenylhydrazine **4** (0.5–1.5 equiv); 2) concentration in Step 1 (1.0–2.4 M); 3) residence time in Step 1 (0.5–1.5 min); 4) temperature in Step 1 (10—50 °C); 5) temperature in Step 2 (25—130 °C); 6) equivalents of triethylamine (0.5–3.0); 7) additional solvent in Step 2 (0–1 × Step 1 total flow rate). Three optimization objectives were defined: 1) maximize solution yield of **6**, as measured by NMR analysis; 2) maximize space‐time yield of **7**, based on FTIR analysis 3) minimize overall equivalents of reagents used (**4** + triethylamine). This combination of objectives, optimizing both reaction steps simultaneously, makes use of both PAT instruments and provides a challenging optimization problem, which will require a trade‐off between different objectives.

**Figure 5:**
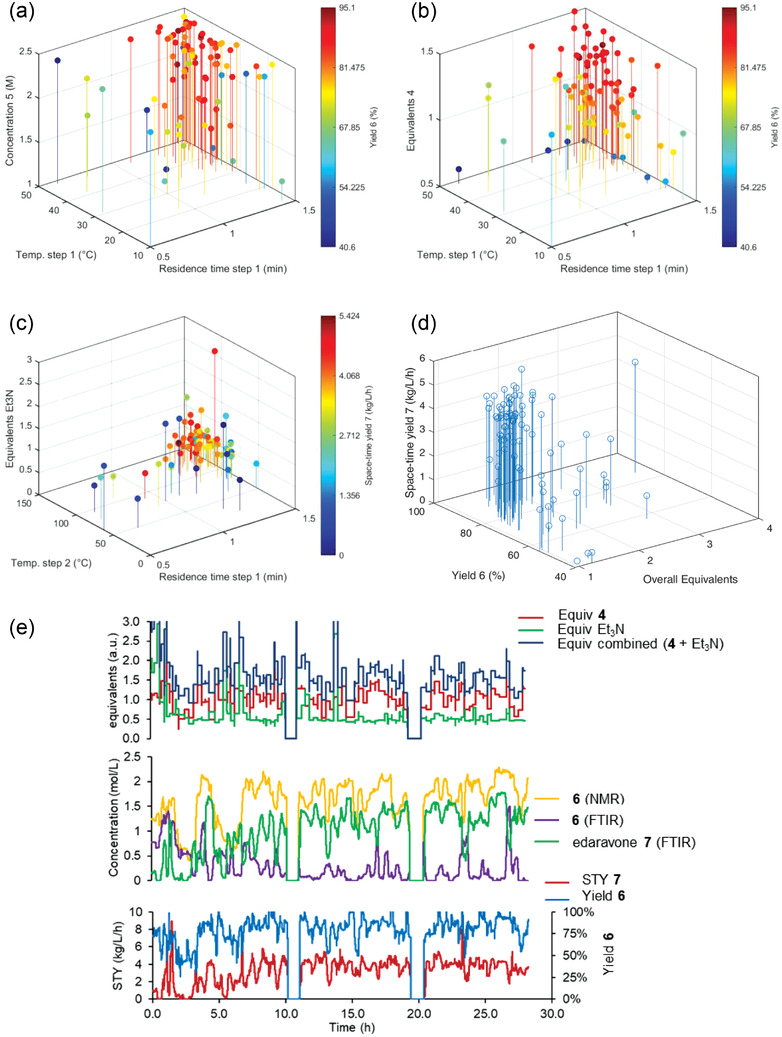
Plots showing the solution yield (step 1) or space‐time yield (step 2) achieved as a function of various optimizable parameters, in a two‐step self‐optimization experiment. a) and b) solution yield 1 versus 3 variables; c) space‐time yield versus 3 variables; d) Plot of all three objectives during the optimization experiment; e) Data readout from the two‐step self‐optimization experiment, showing reagent equivalents as well as NMR and FTIR measured concentrations and both solution yield/space‐time yield objectives over time. Note: breaks at ≈10 and ≈20 hours are present because the reactor was shut down overnight.

The optimization experiment was initiated from the center point of the design space (c.f. Table 1, Entries 4 and 5), which was expected to provide the most expeditious optimization. Over the course of ≈26 h, 85 optimization iterations were run, achieving >95% solution yield of imine intermediate **6** and a 5.42 kg L^−1^ h^−1^ space‐time yield of edaravone. Plots showing the results of each iteration are shown in **Figure** **5**. The solution yield of imine **6** increasing with increased concentration and temperature, as well as longer residence time (Figure 5a). The addition of a third optimization objective (minimizing equivalents of reagents in both steps) served as an additional weighting, to prevent the overuse of excess reagents. As a result, the optimization algorithm attempted to reduce the equivalents of phenylhydrazine **4** (“equivalents 1”), whilst also retaining high values for the other two objectives (Figure 5b).

In the second reaction step, the effects of minimizing reagent excess can be seen more clearly (Figure 5c). It appears that substoichiometric quantities of triethylamine are sufficient to promote the reaction, as evident by most experimental conditions using between 0.5–1.0 equiv triethylamine. This contributes to a total reagent excess of less than 2 in most iterations, which serves to decrease wastage. When considering all three objectives (Figure 5d), it is clear that no single solution provides optimal values for each. However, a distinct cluster of results exists in the top left corner (high solution yield 1, high space‐time yield, but low overall equivalents), within which the ideal conditions can be chosen. As in the previous experiment, all transient data were also recorded (Figure 5e), showing the set points and responses for all 85 iterations.

This experiment was estimated to consume roughly 14.3 g h^−1^ of starting material **5**, which does represent a significant quantity. Accordingly, it is envisaged that this approach to reaction to optimization would be invoked at an appropriate state in the reaction/process development timeline, where significant quantities of material are already available. The high material consumption per iteration can be put down to the requirement for the reactor to reach a steady state, even with the implemented steady‐state monitoring feature. Incorporation of a droplet generating system^[^ ^26^ ^]^ could be used to improve the applicability of this platform to earlier stage of development, when materials are scarcer. Alternatively, the material consumption could also be limited in future developments by leveraging dynamic experimentation, to capitalize further on the rapid analytical measurements.

## 3 Conclusion

We have developed a flow reactor platform for autonomous self‐optimization of complex reaction systems, based on rapid spectroscopic measurements, processed using chemometric models. The efficiency of the system was demonstrated with two case studies. The first reaction, an S_N_Ar reaction with four optimizable variables, was examined using multiple different starting points for initial model building prior to self‐optimization experiments. Here, it was found that beginning with a single experiment in the center of the design space provided the fastest path to optimal conditions. This knowledge was then taken on to the optimization of a significantly more complex reaction system, comprising of two chemical steps with seven optimizable variables. Due to this increased complexity, a larger number of iterations (85) were allowed, to explore the design space. Within this period, a 5.42 kg L^−1^ h^−1^ space‐time yield of the API product edaravone **7** was achieved, whilst minimizing reagent excesses. This represents a marked advance in the complexity of autonomous flow reactors in both the quantity of obtained data and the size of the optimizable design space.

## Conflict of Interest

The authors declare no conflict of interest.

## Supporting information

Supporting Information

## Data Availability

The data that support the findings of this study are available from the corresponding author upon reasonable request.
