# Supplementary material for: Autonomous Multi‐Step and Multi‐Objective Optimization Facilitated by Real‐Time Process Analytics
Source: Adv Sci (Weinh). 2022 Feb 1;9(10):2105547. doi: 10.1002/advs.202105547 (PMC8981902; doi:10.1002/advs.202105547)
Supplement: Supplementary file 1 — Supporting Information [file ADVS-9-2105547-s001.pdf]

ADVANCED SCIENCE
Open Access

Supporting Information

for *Adv. Sci.*, DOI 10.1002/advs.202105547

Autonomous Multi-Step and Multi-Objective Optimization Facilitated by Real-Time Process Analytics

Peter Sagmeister, Florian F. Ort, Clemens E. Jusner, Dominique Hebrault, Thomas Tampone, Frederic G. Buono, Jason D. Williams and C. Oliver Kappe**

ADVANCED SCIENCE
Open Access

Supporting Information

for *Adv. Sci.*, DOI: 10.1002/advs.202105547

Autonomous Multi-Step and Multi-Objective Optimization Facilitated by Real-Time Process Analytics

Peter Sagmeister, Florian F. Ort, Clemens E. Jusner, Dominique Hebrault, Thomas Tampone Frederic G. Buono, Jason D. Williams and C. Oliver Kappe**

Supporting Information

Autonomous Multi-Step and Multi-Objective Optimization Facilitated by Real-Time Process Analytics

Peter Sagmeister, Florian F. Ort, Clemens E. Jusner, Dominique Hebrault, Thomas Tampone Frederic G. Buono, Jason D. Williams and C. Oliver Kappe**

1 Experimental Details 4
1.1 General experimental details 4
2 Control Software 7
2.1 Process visualization 7
2.2 Design of the programmable logic controller (PLC) 8
2.2.1 Single step optimization 8
2.2.2 Multi-step optimization 10
2.2.3 Applied Filter on the Data recorded from NMR and FTIR 12
3 Optimization Algorithm Software 13
4 PAT Instrument Details 14
4.1 Inline NMR 14
4.1.1 General Details 14
4.1.2 Process Integration (S_NAr) 14
4.1.3 Data Analysis Through Indirect Hard Modelling (S_NAr) 15
4.1.4 Process Integration (Edaravone) 17
4.1.5 Data Analysis Through Indirect Hard Modelling (Edaravone) 18
4.2 Inline FTIR 21
4.2.1 General Details 21
4.2.2 Process Integration (Edaravone) 22
4.2.3 Data Analysis Through Partial Least Squares (PLS) Regression (Edaravone) 22
5 S_NAr 26
5.1 Reactor Platform 26
5.2 Reactor Inputs and Reactor Outputs 28
5.3 Optimization Parameters 29
5.4 Reaction Optimization (S_NAr) 31
5.4.1 Batch Experiments 31
5.4.2 Self-Optimization (LHC) 32
5.4.3 Self-Optimization (Restricted full factorial DoE) 35
5.4.4 Self-Optimization (Full factorial DoE) 38
5.4.5 Self-Optimization ("center points") 41
5.4.6 Self-Optimization (3 Objective) 47
6 Edaravone 50
6.1 Reactor Platform 50
6.2 Reactor Inputs and Reactor Outputs 53
6.3 Optimization Parameters 55

6.4 Reaction Optimization (Edaravone Synthesis) ... 56
6.4.1 Batch Experiments ... 56
6.4.2 Self-Optimization ... 57
7 NMR Spectra ... 61
8 References ... 64

1 Experimental Details

1.1 General experimental details

Solvents and chemicals were purchased from commercial suppliers and were used without further purifications. For the S_NAr reaction 3,4-difluoronitrobenzene (purity >98%) and 1,8-diazabicyclo[5.4.0]undec-7-ene (DBU, purity 98 %) were obtained from TCI. Morpholine (purity >99%) and triethylamine (purity >99.5%), were purchased from Sigma Aldrich. Acetonitrile (MeCN) (HPLC-grade) and Methanol (MeOH) (HPLC-grade) were obtained from VWR. For the edaravone synthesis, phenylhydrazine (purity 97%), ethyl acetoacetate (purity >99%) and triethylamine (purity 99.5%) were purchased from Sigma Aldrich and ethanol (EtOH) (purity 99%) from VWR.

High field NMR spectra were recorded on a Bruker 300 MHz instrument. ^{1}H spectra were recorded at 300 MHz, respectively, with a chemical shift (δ) relative to the methyl group (3.31 ppm) of methanol-d_4 expressed in parts per million. The letters s, d, dd, td, t, and m are used to indicate singlet, doublet, doublet of doublets, triplet of doublets, multiplet respectively.

High resolution mass spectrometry (HR-MS) measurements were performed using a Q-Exactive Hybrid Quadrupole-Orbitrap MS following flow injection analysis of the dissolved (in acetonitrile) sample with a Dionex Ultimate 3000 series HPLC-system (Thermo Fisher Scientific). The injection volume was 5 µL and the flow was 200 µL/min of acetonitrile. The HR-MS was fitted with a HESI-II atmospheric pressure electrospray ionization source.

Self-optimization reactions were performed using the automated continuous flow chemistry platform at the Kappe Laboratories in Graz. This platform is comprised of a Supervisory Control and Data Acquisition (SCADA) software (Evon, XAMControl), which is further connected to a Distributed Control System (DCS) (HiTec Zang, LabVision software and LabManager hardware), which communicates to actuators and sensors. The platform includes several syringe pumps (HiTec Zang, SyrDos2 equipped with high- or low pressure pump heads) and HPLC pumps (Knauer, AZURA P 4.1S with 10 mL or 50 mL pump heads made out of stainless steel, ceramic or Hastelloy). Additionally, the platform includes thermostats (Huber, Ministat 240 and CC-304), gas and liquid mass flow controllers (Bronkhorst), pressure controllers (Bronkhorst, EL-PRESS) and a hydrogen generator (Thales Nano Energy, H-Genie). A modular micro reaction system (Ehrfeld, MMRS), a shell-and-tube reactor

(Ehrfeld, Miprowa Lab reactor) provides the flexibility of performing different reaction types in the automated platform. Several real-time PAT instruments such as temperature and pressure sensors, FTIR (Mettler Toledo, ReactIR 15), benchtop NMR (Magritek, Spinsolve Ultra), UV-vis spectrometer (Avantes, AvaSpec ULS2048) and online UHPLC (Shimadzu, Nexera X2) are established within the platform. The automated data processing is accomplished with PEAXACT and ProcessLink (S-PACT), Matlab or Python. Advanced Process Control is enabled by communication to Matlab, Python or DLLs embedded in XAMControl.

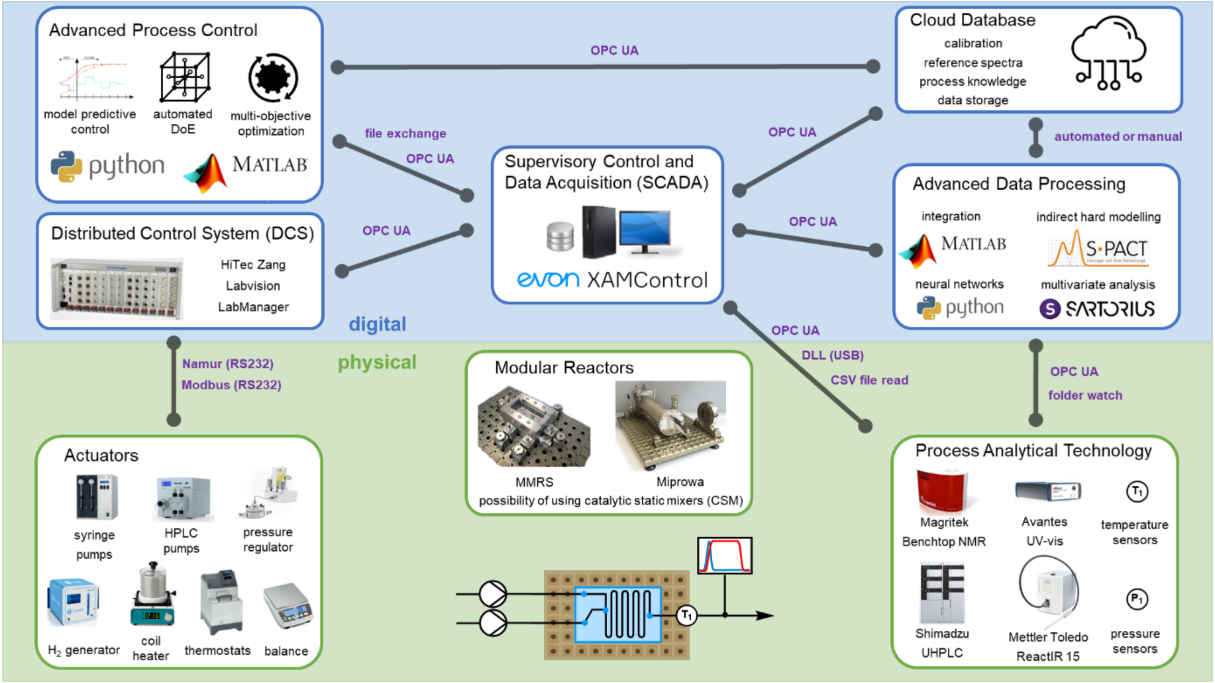

Figure S1. Schematic overview of the automated modular continuous flow chemistry platform at the Kappe Lab. Green background represents the physical world and blue the digital.

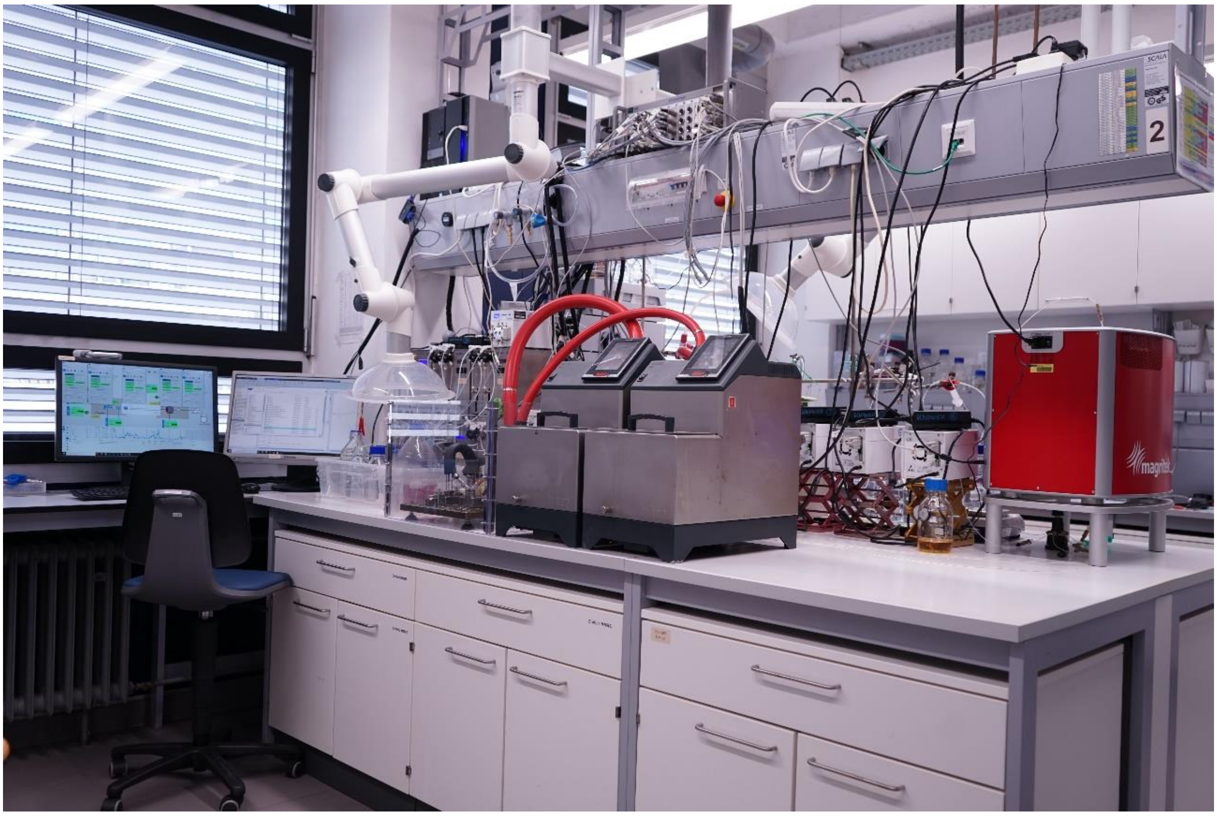

Figure S2. Photograph of the automated modular continuous flow chemistry platform at the Kappe Lab.

2 Control Software

XAMControl is a SCADA software for industrial automation. It allows direct communication with actuators and sensor with different field bus protocols as well as communication via OPC UA to different DCS systems. The process is visualized in XAMControl Iris (**Figure S3** and **Figure S4**), which allows the display of real-time process data and manual process control from the operator. The backbone of XAMControl is the designer, which allows PLC integration either with object orientated programming (**Figure S5** and **Figure S9**) or coding in C# programming language (**Figure S6** to **Figure S8** and **Figure S10**). All recorded data points are stored in a cloud repository and can be accessed with XAMControl Iris or be exported to csv files.

2.1 Process visualization

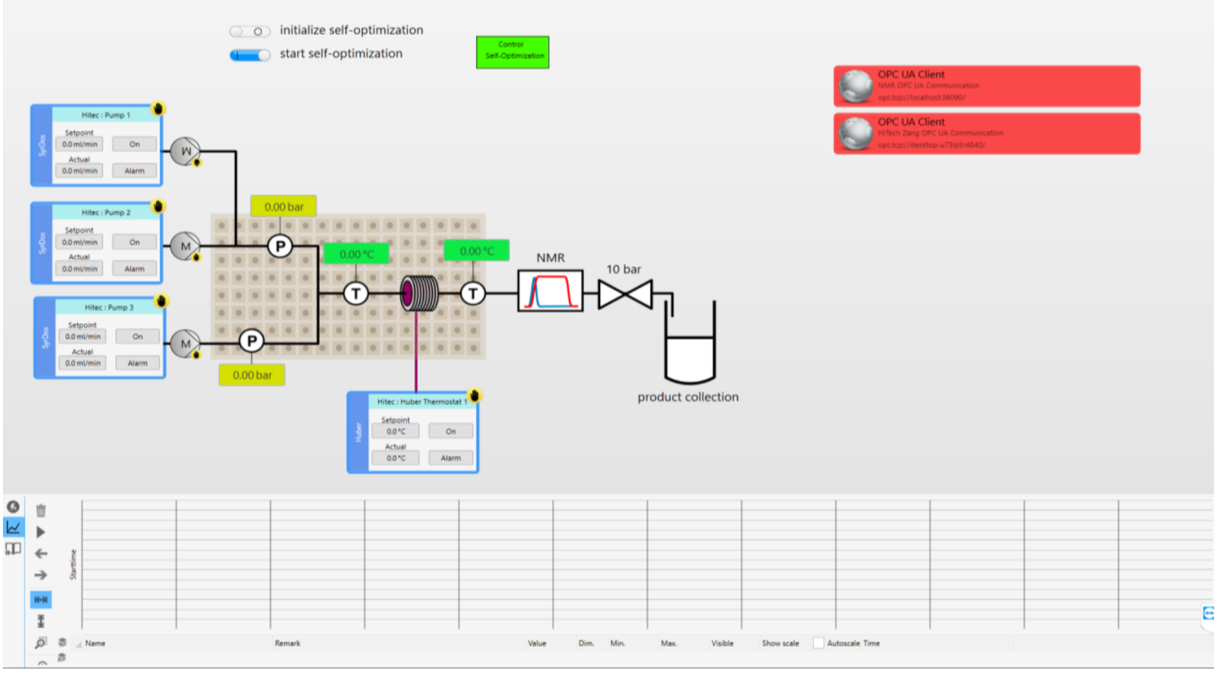

Figure S3. Process visualization in XAMControl for the single step self-optimization. The lab equipment and PAT can be monitored and controlled from this view.

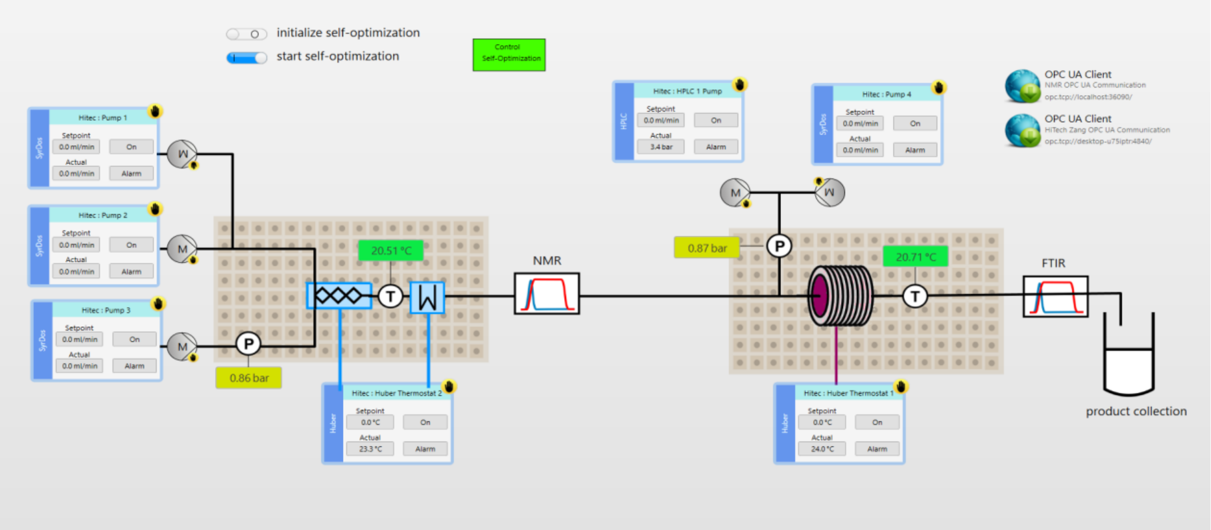

Figure S4. Process visualization in XAMControl for the multi-step self-optimization experiments. The lab equipment and PAT can be monitored and controlled from this view.

2.2 Design of the programmable logic controller (PLC)

2.2.1 Single step optimization

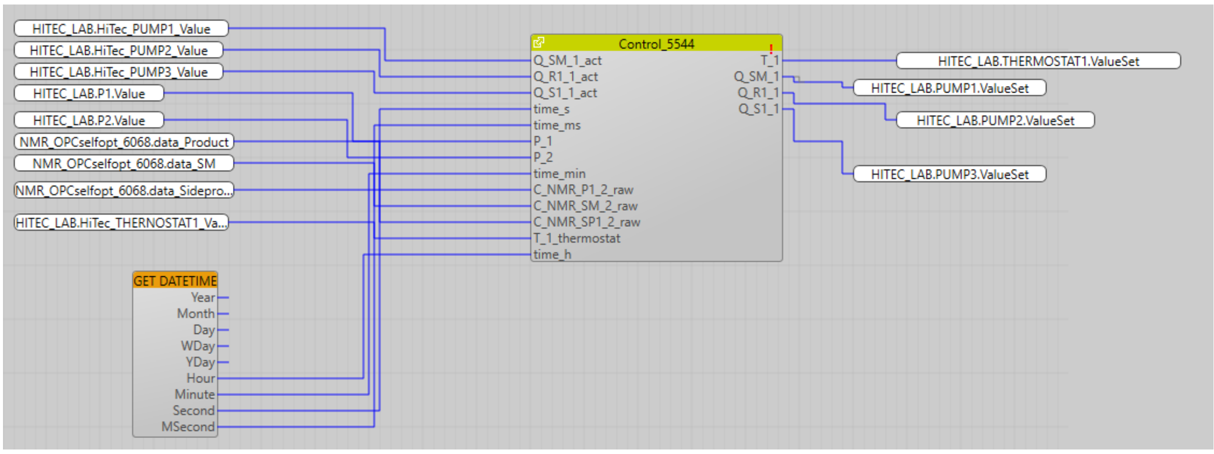

Figure S5. Object oriented PLC design in XAMControl with the different inputs and outputs of the PLC for the single step optimization.

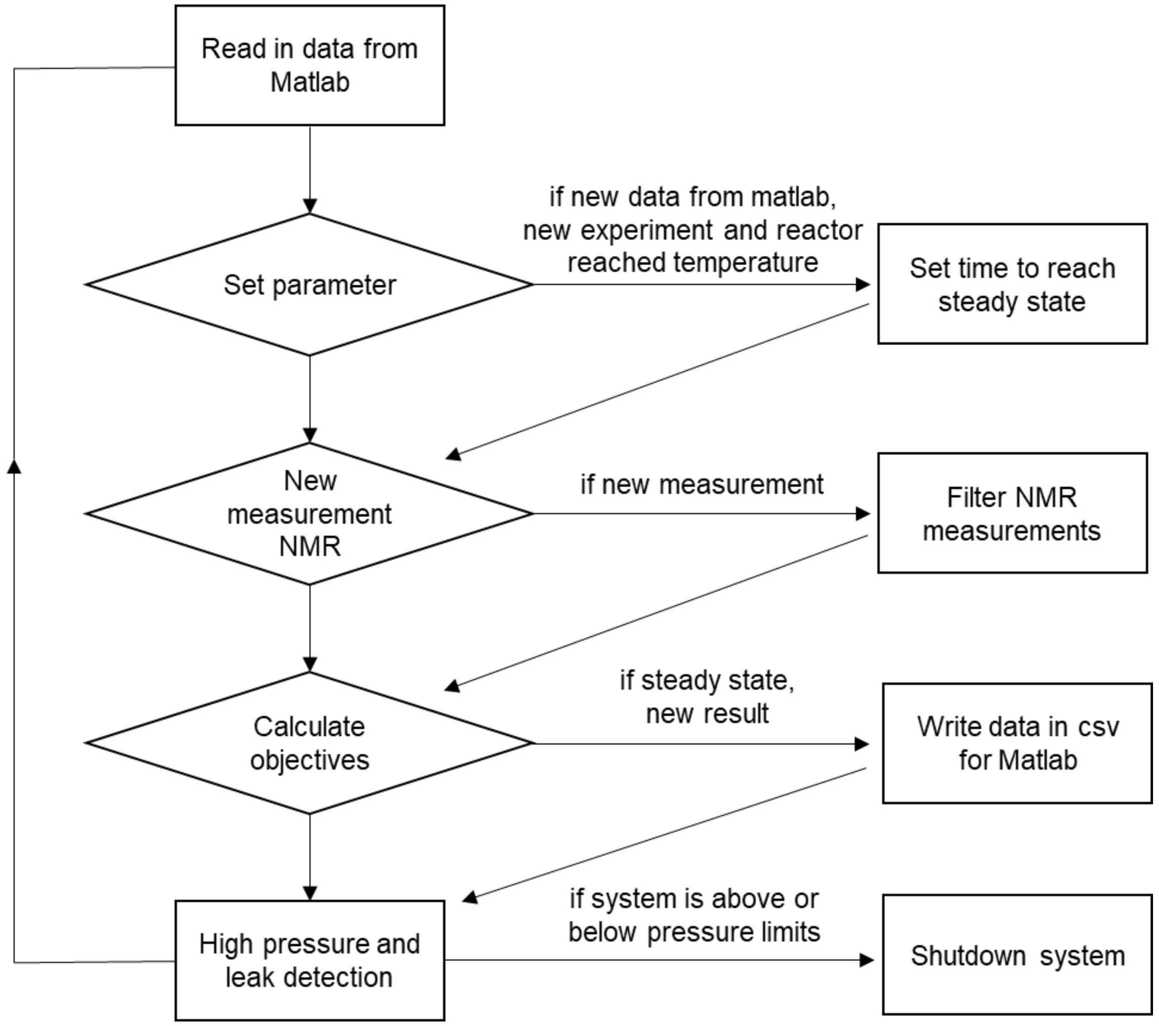

Figure S6. A flowchart of the PLC in XAMControl for the single step optimization.

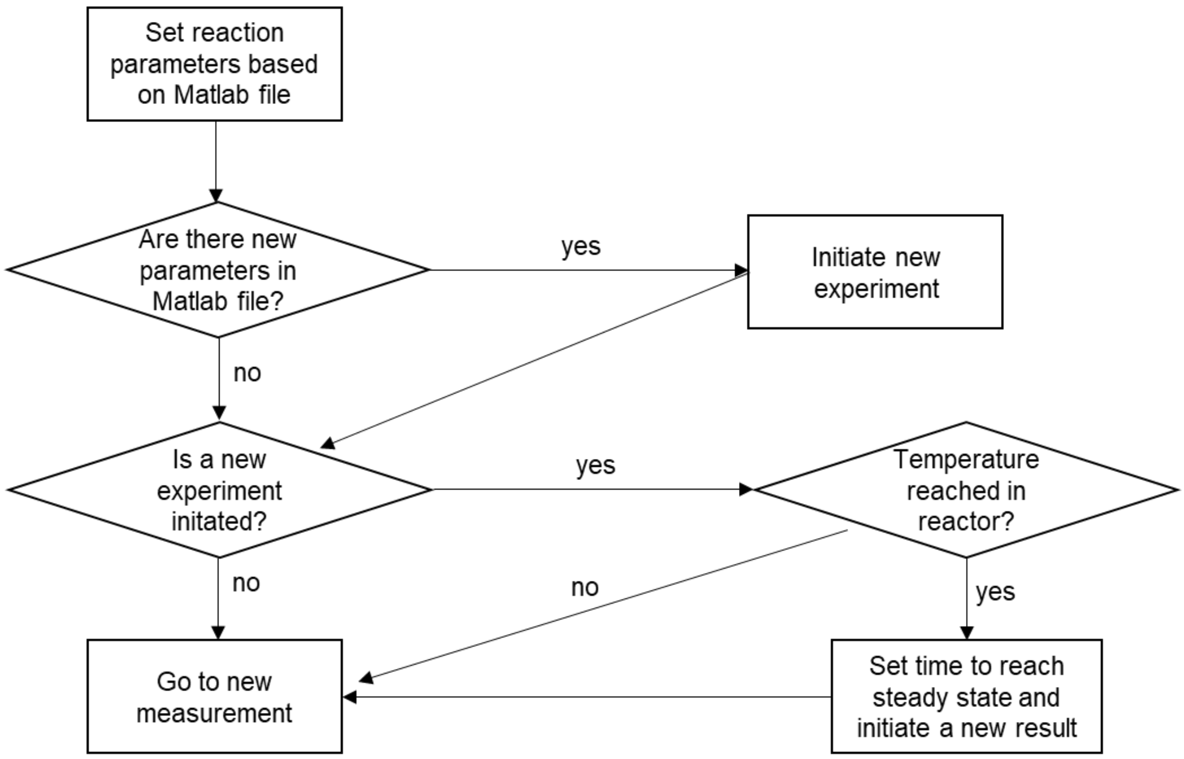

Figure S7. A flowchart of the method (Set parameters) in XAMControl for the single step optimization.

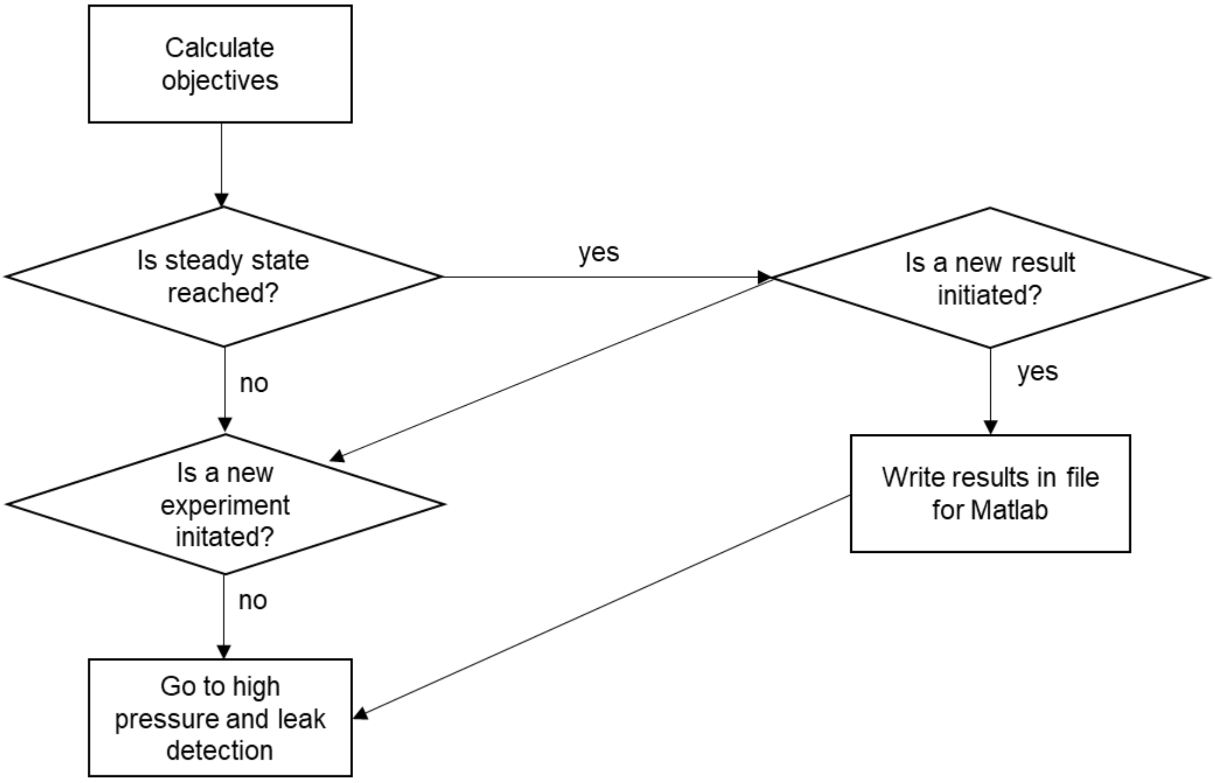

Figure S8. A flowchart of the method (Calculate objectives) in XAMControl for the single step optimization.

2.2.2 Multi-step optimization

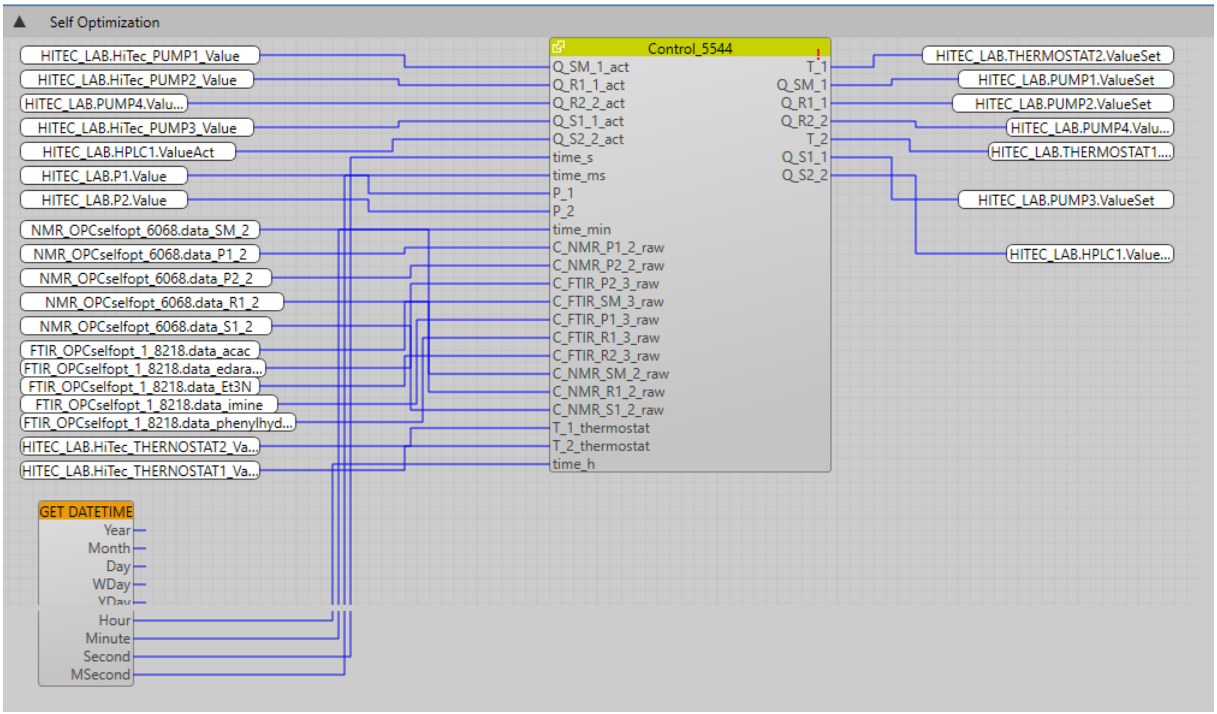

Figure S9. Object oriented PLC design in XAMControl with the different inputs and outputs of the PLC for the multi-step optimization.

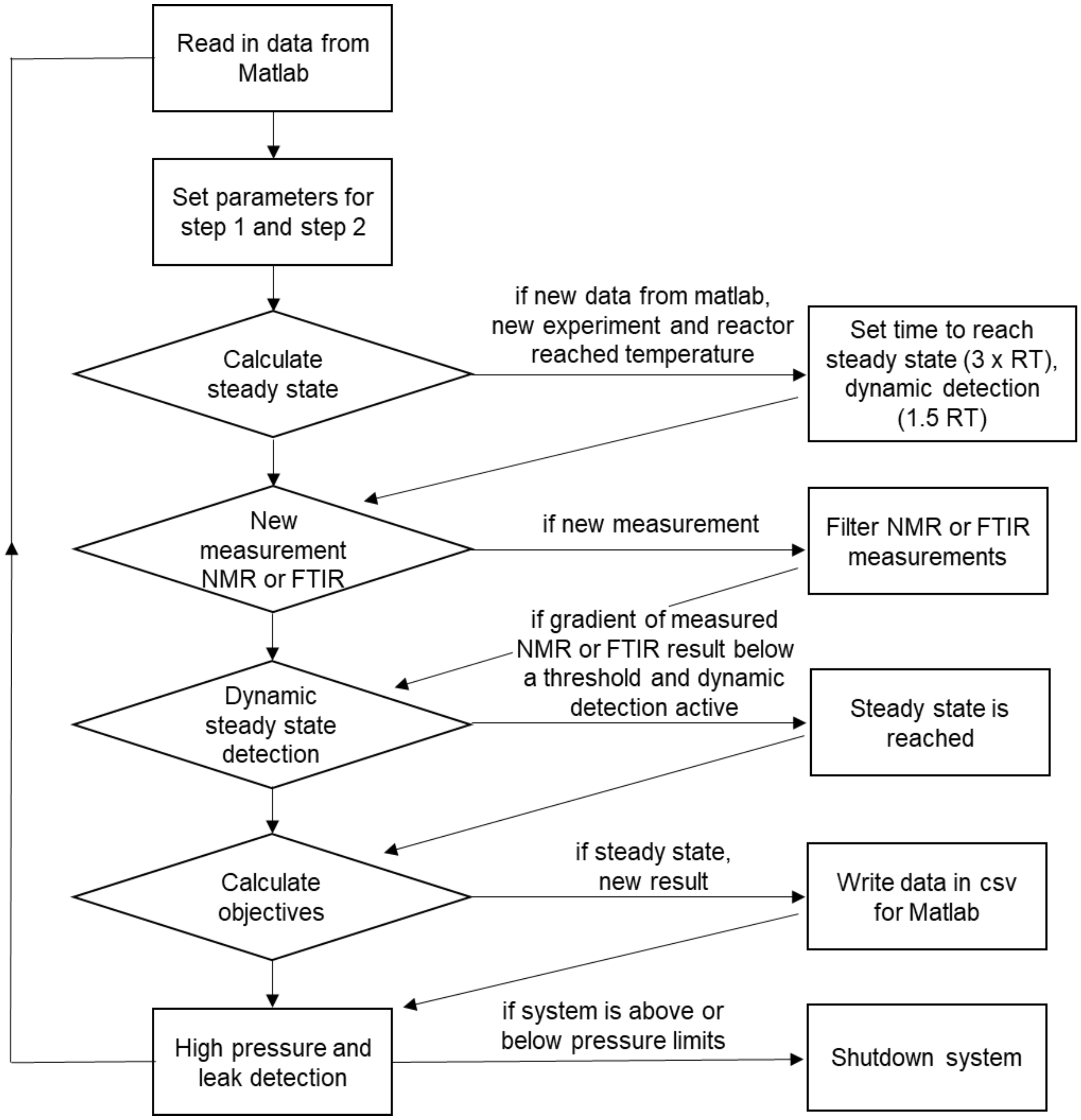

Figure S10. A flowchart of the PLC in XAMControl for the single step optimization.

2.2.3 Applied Filter on the Data recorded from NMR and FTIR

Raw data sent by ProcessLink via OPC UA to XAMControl were saved in the database and further processed. The processing was done automatically by embedding Matlab programs as DLLs in the XAMControl PLC. First a filter (CutPeaksFilter) was applied to smooth sudden changes (e.g. in case of an outlier because of an air bubble going through the NMR). This CutPeaksFilter is calculating a threshold from the last 10 measured points, if the next result exceeds the threshold, the data point is removed. Additionally, a new data point is inserted based on the average of the last measurement points and the trend of the outlier. The second filter was a finite impulse response filter (FIR-filter) which was based on the last 10 measurement points. In **Figure S11** an example of the filtered data compared to the real-time raw data is displayed.

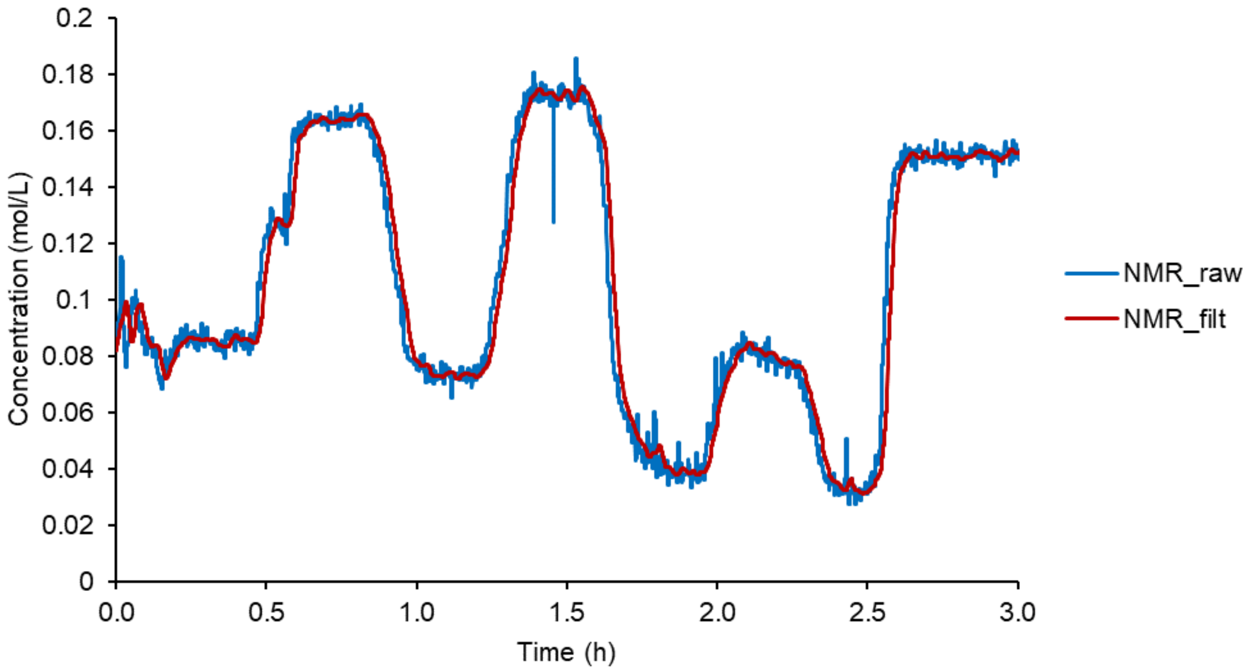

Figure S11. An example for the comparison of raw NMR data (blue) and filtered NMR data (red).

3 Optimization Algorithm Software

The optimization algorithm TS-EMO was adapted from Schweidtmann et al. and changed in minor parts.[1] This included a file exchange between the SCADA software (XAMControl) and the Matlab interface. Additionally, a Matlab program was written to automatically create and execute full factorial DoE designs, LHC or single experiment points. These initial experiments were typically sorted in order of increasing temperature to reduce the experimental time.

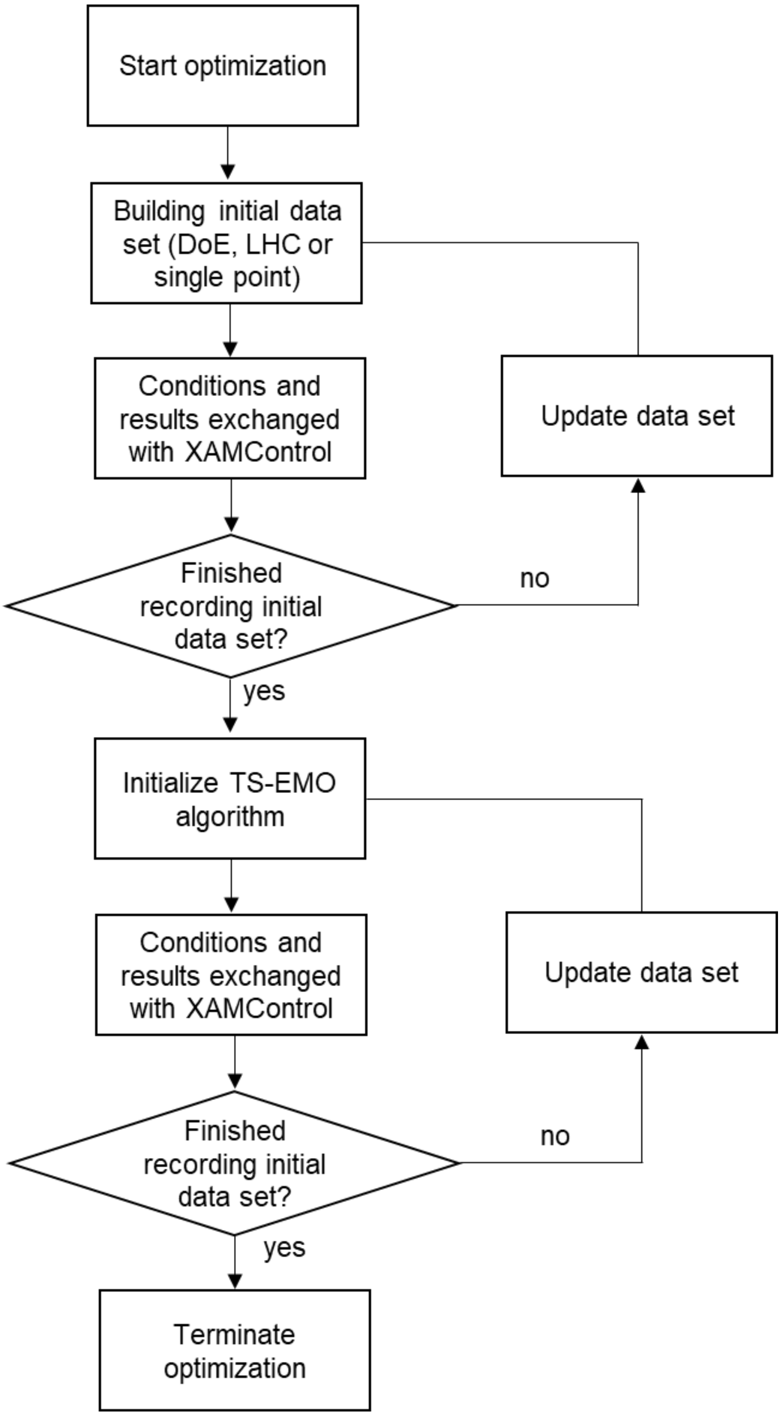

Figure S12. A flowchart of the optimization algorithm in Matlab.

4 PAT Instrument Details

4.1 Inline NMR

4.1.1 General Details

Inline reaction monitoring of ^{1}H-NMR was accomplished by using a benchtop 43.795 MHz spectrometer (Magritek, Spinsolve Ultra 43 MHz). The benchtop NMR was typically shimmed on a regular basis with a mixture of deionized H_2O (10%) and D_2O (90%) ("QUICKSHIM ALL", linewidth at 50%: <0.4 Hz, linewidth at 0.55%: <8.0 Hz, signal to noise ratio: >20,000). Additionally, shims were directly performed with the flow cell for the S_NAr reaction on MeCN or the reaction mixture ("QUICKSHIM ALL", linewidth at 50%: <0.6 Hz, linewidth at 0.55%: <9.0 Hz, signal to noise ratio: >16,000). For the edaravone synthesis shims were performed with standard 1/8" o.d. (1.6 mm i.d.) PTFE tubing on EtOH or the reaction solution ("QUICKSHIM ALL", linewidth at 50%: <1.0 Hz, linewidth at 0.55%: <25.0 Hz, signal to noise ratio: >2,300). The spectra for the S_NAr reaction were recorded in the reaction monitoring mode with a pulse angle of 90°, acquisition time of 6.4 s, repetition time of 10.0 s and a single scan. The spectra for the edaravone synthesis were recorded in the reaction monitoring mode with a pulse angle of 90°, acquisition time of 1.6 s, repetition time of 2.0 s and 4 scans. ProcessLink (S-PACT) enabled automated reading and processing of the recorded spectra by using an indirect hard model (model development for the S_NAr and edaravone synthesis see section 4.1.3 and 4.1.5, respectively).

4.1.2 Process Integration (S_NAr)

The reaction monitoring was with benchtop NMR was enabled by using the Magritek reaction monitoring kit (Kit RM2) which is comprised of a glass flow cell. Prior to entering the NMR, a T-piece with a back pressure regulator (2.8 bar) was installed. In case of a blockage (causing an increase in pressure) within the NMR flow-through cell, it would open to avoid any damage to the NMR instrument. The reaction mixture then entered a 6-port valve at port 4 prior to analysis with the benchtop NMR (**Figure S13**). When in position P 1, the process stream left the valve through port 3 and went through the benchtop NMR glass flow-through cell (internal volume = 800 µL, length = 550 mm) and entered again at port 6. Finally, the process stream left for the next reaction step through port 5. The 6-port valve in position P 2 allowed the NMR to be shimmed on MeCN or process solution, without any solution flowing through the NMR.

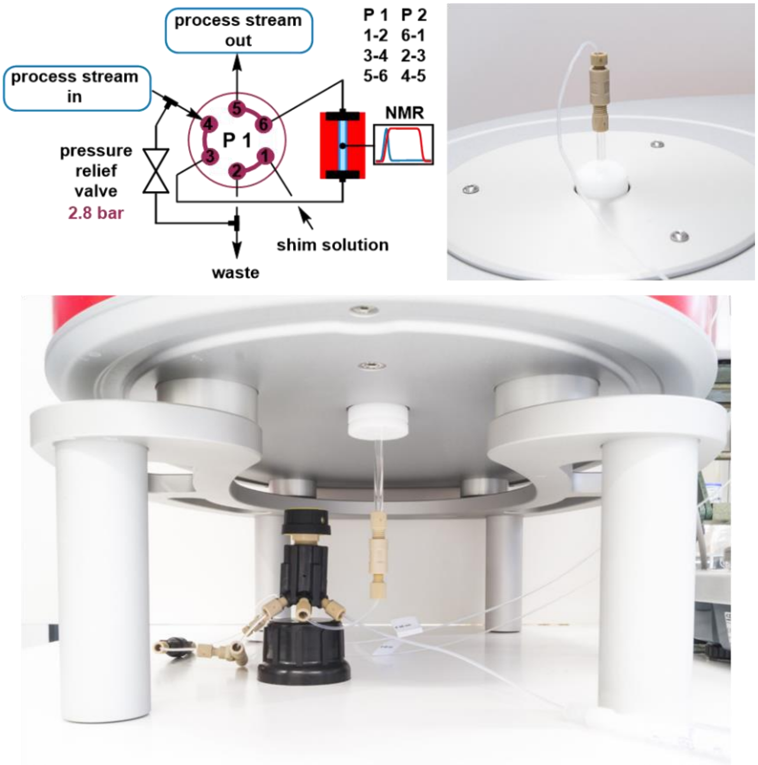

Figure S13. An overview of the NMR configuration using the glass flow cell reaction monitoring kit (Magritek, Kit RM2)

4.1.3 Data Analysis Through Indirect Hard Modelling (S_NAr)

Recording of training and validation data

Preparation of training and validation solutions were performed by weighing the corresponding amount of 3,4-difluoronitrobenzene **2**, linezolid intermediate **3**, triethylamine into 10 mL volumetric flasks. The flasks were then filled up to the 10 mL mark with a mixture of MeOH and MeCN (1 + 1 v/v) and sonicated to encourage dissolution. The prepared solutions were stored at room temperature until the start of the measurement and were pumped with a peristaltic pump (Ismatec, ISM834C) through the benchtop NMR. The pump was set to 10 rpm, which corresponds to a flow rate of approximately 1 mL/min. The tubing and NMR flow cell were flushed first with air, then with a mixture of MeOH and MeCN (1 + 1 v/v) and with air again to avoid cross-contamination. For each training and validation level, 100 spectra were acquired (number of scans: 1, repetition time: 10 seconds, pulse angle: 90 °, acquisition time: 6.4 seconds).

Table S1. Overview of the prepared solutions for building the S_NAr NMR model.

Entry	Usage	3,4-difluoronitrobenzene **2** (mol/L)	linezolid intermediate **3** (mol/L)
Pure_2	Train	0.494	0
Pure_3	Train	0	0.285
Level_1	Train	0.365	0.131
Level_2	Train	0.251	0.243
Val_1	Test	0.167	0.330

Workflow for indirect hard modelling

The acquired spectra for each model were loaded into PEAXACT, assigned labels and grouped into different levels. A representative spectrum for each level was obtained by calculating the mean of the individual spectra.

Pretreatment model: All spectra underwent the same pretreatment conditions: base line correction (linear fit subtraction), phasing (Auto, Negative Peak Penalization), smoothing (filter size: 5), and spectral alignment of the highest peak (MeOH) to 3.43 ppm. The global range was from 6 ppm to 9 ppm.

Generation of pure component models: Peaks were added empirically and stepwise to the model (20 – 40 peaks per model) until the residuals were roughly two orders of magnitude lower than the largest peak. The fitting mode was set to maximal interactions, allowing the greatest flexibility within the model.

Generation of mixture model: A weighted sum of each pure component model represents the mixture model, including flexible but constrained peak parameters.

Calibration model and validation: The training set was comprised of the pure component spectra and two different component mixtures (Level_1 and Level_2). The calibration model provided a performance indicator of model error, the root-mean-square error of calibration ($RMSE_C$). Additionally, to perform cross-validation (CV), the training set was divided into subgroups (leave group out) by concentration level. The CV algorithm generates reduced data sets to get a performance indicator of model error, the root-mean-square error of cross validation ($RMSE_{CV}$). To validate the model, the root-mean-square error of validation ($RMSE_V$) was calculated from the validation set (Val_1).

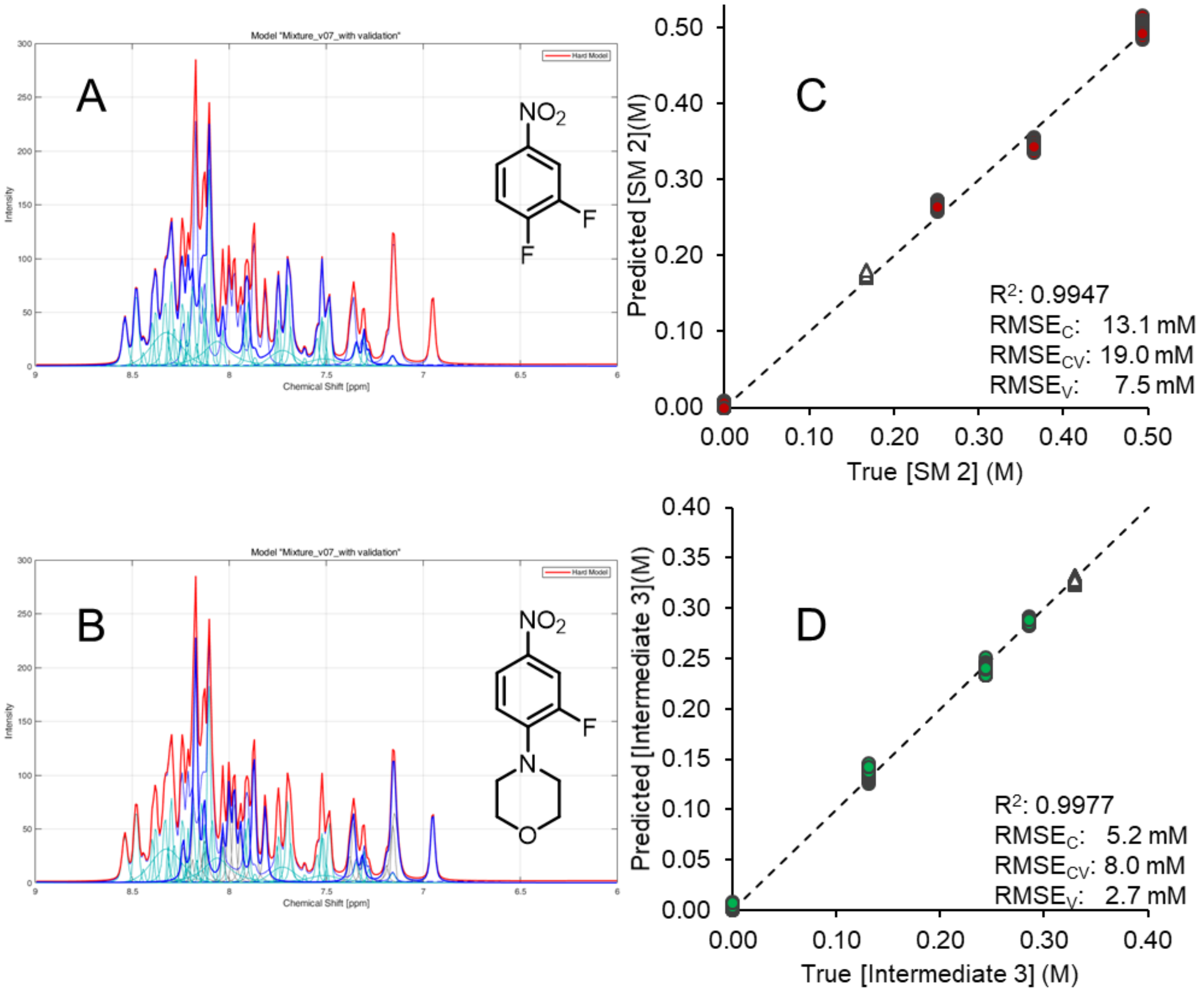

Figure S14. The mixture model is highlighted in spectra A and B (red). The pure component models (blue) of the starting material 3,4-difluoronitrobenzene **2** and the linezolid intermediate **3** in A and B, respectively. Parity plots are shown in C and D with the target line (y = x). Colored circles show the training data, white triangles show the validation data. Relevant statistical data is provided in the parity plots ($RMSE_C$: error of calibration, $RMSE_{CV}$: error of cross validation, $RMSE_V$: error of validation)

4.1.4 Process Integration (Edaravone)

The reaction monitoring was with benchtop NMR was enabled by using the Magritek reaction monitoring kit (Kit RM1) which is comprised of a glass tube guide and a length of PTFE tubing. The entire process stream entered a 6-port valve at port 4 (**Figure S15**) prior to analysis with the benchtop NMR. When in position P 1, the process stream left the valve through port 3 and went through the benchtop NMR via a PTFE tube (1/8” o.d., 1.6 mm i.d., length = 600 mm, internal volume = 1.14 mL) and entered again at port 6. Finally, the process stream left for the next reaction step through port 5. The 6-port valve in position P 2 allowed the NMR to be shimmed on EtOH or process solution, without any solution flowing through the NMR.

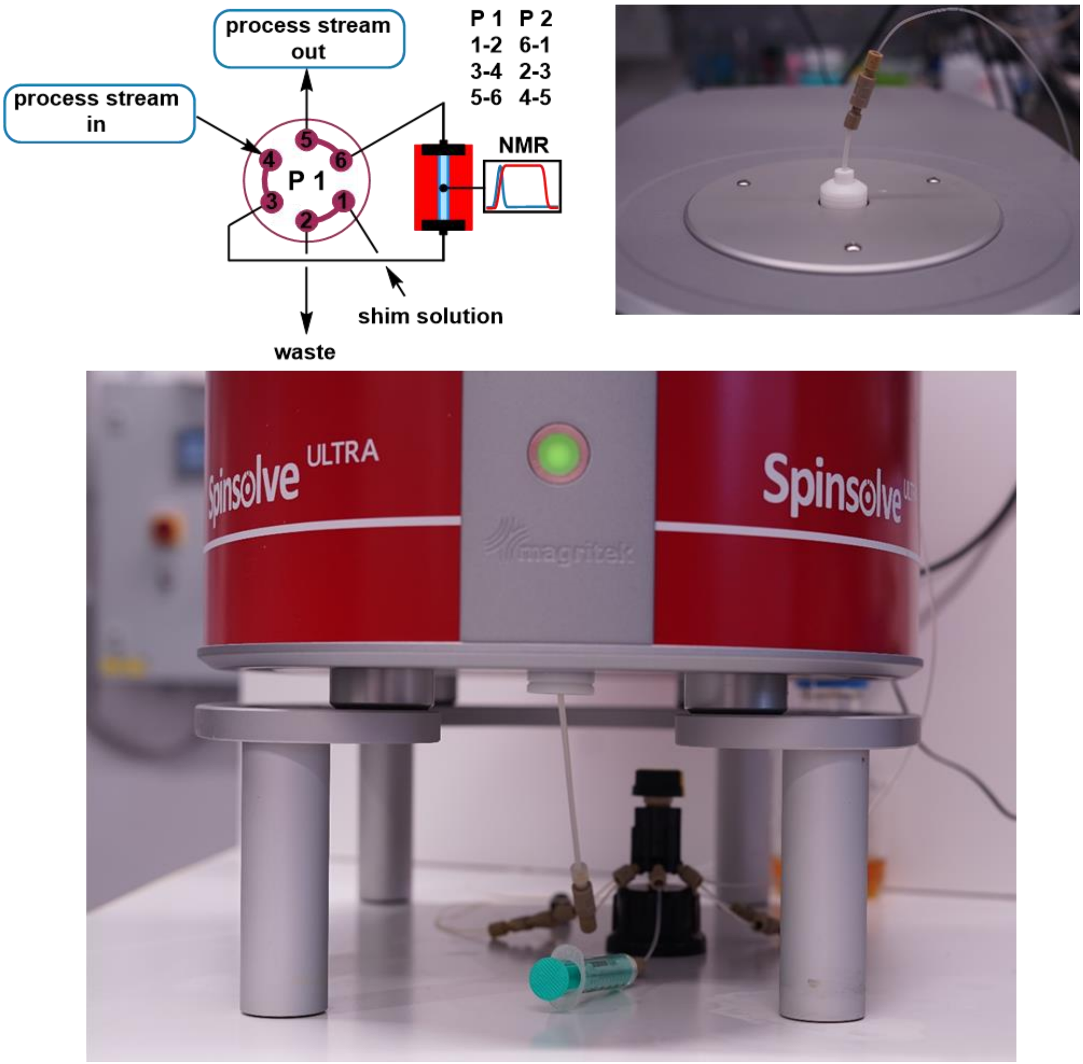

Figure S15. An overview of the NMR configuration using the PTFE tubing reaction monitoring kit (Magritek, Kit RM1)

4.1.5 Data Analysis Through Indirect Hard Modelling (Edaravone)

Recording of training and validation data

Preparation of training and validation solutions was performed by weighing the corresponding amount of phenyl hydrazine, ethyl acetoacetate, imine (**6**) and edaravone (**7**) into either 5 or 10 mL volumetric flasks. The flasks were then filled up to the 5 or 10 mL mark with EtOH (96 %) and sonicated. The prepared solutions were stored at room temperature until the start of the measurement and were pumped with a peristaltic pump (Ismatec, ISM834C) through the benchtop NMR. The pump was set to 20 rpm which corresponds to a flow rate of approximate 2 mL/min. The tubing and NMR flow cell were flushed first with air, then with EtOH and with air again to avoid cross-contamination. For each training and validation level, 100 spectra were acquired (number of scans: 4, repetition time: 2 seconds, pulse angle: 90 °, acquisition time: 1.6 seconds).

Table S2. Overview of the prepared solutions for building the edaravone NMR model.

Entry	Usage	Phenylhydrazine **4** (mol/L)	Ethyl acetoacetate **5** (mol/L)	Imine **6** (mol/L)
Pure_1	Train	0	0	0
Pure_2	Train	0	0	1.895
Pure_3	Train	1.935	0	0
Pure_4	Train	0	2.425	0
Val_1	Test	0.806	0	1.830
Level_1	Train	0.395	0	1.601
Level_2	Train	1.199	0	2.198
Val_2	Test	0	0.499	0.916
Level_3	Train	0	0.749	1.373

Workflow for indirect hard modelling

The acquired spectra for each model were loaded into PEAXACT and assigned with labels and grouped into different levels. A representative spectrum for each level was obtained by calculating the mean of each individual spectrum.

Pretreatment model: All spectra underwent the same pretreatment conditions: zero filling (32k), phasing (Auto, Negative Peak Penalization) and spectral alignment of the highest peak (EtOH) to 1.19 ppm. The global range was from 0 ppm to 11 ppm.

Generation of pure component models: Peaks were added empirically and stepwise to the model (7–17 peaks per model) until the residuals were roughly two orders of magnitude with respect to the largest peak. The fitting mode was set to maximal interactions, allowing the greatest flexibility within the model.

Generation of mixture model: A weighted sum of each pure component model represents the mixture model, including flexible but constrained peak parameters.

Calibration model and validation: The training set was comprised of the pure component spectra and three different component mixtures (Level_1 to Level_3). The calibration model provided a performance indicator of model error, the root-mean-square error of calibration ($RMSE_C$). Additionally, to perform cross-validation (CV), the training set was divided into subgroups (leave group out) by concentration level. The CV algorithm generates reduced data sets to get a performance indicator of model error, the root-mean-square error of cross

validation ($RMSE_{CV}$). To validate the model, the root-mean-square error of validation ($RMSE_V$) was calculated from the validation sets (Val_1 and Val_2). Two validation sets were required in order to cover all three species due to incompatibilities between some species.

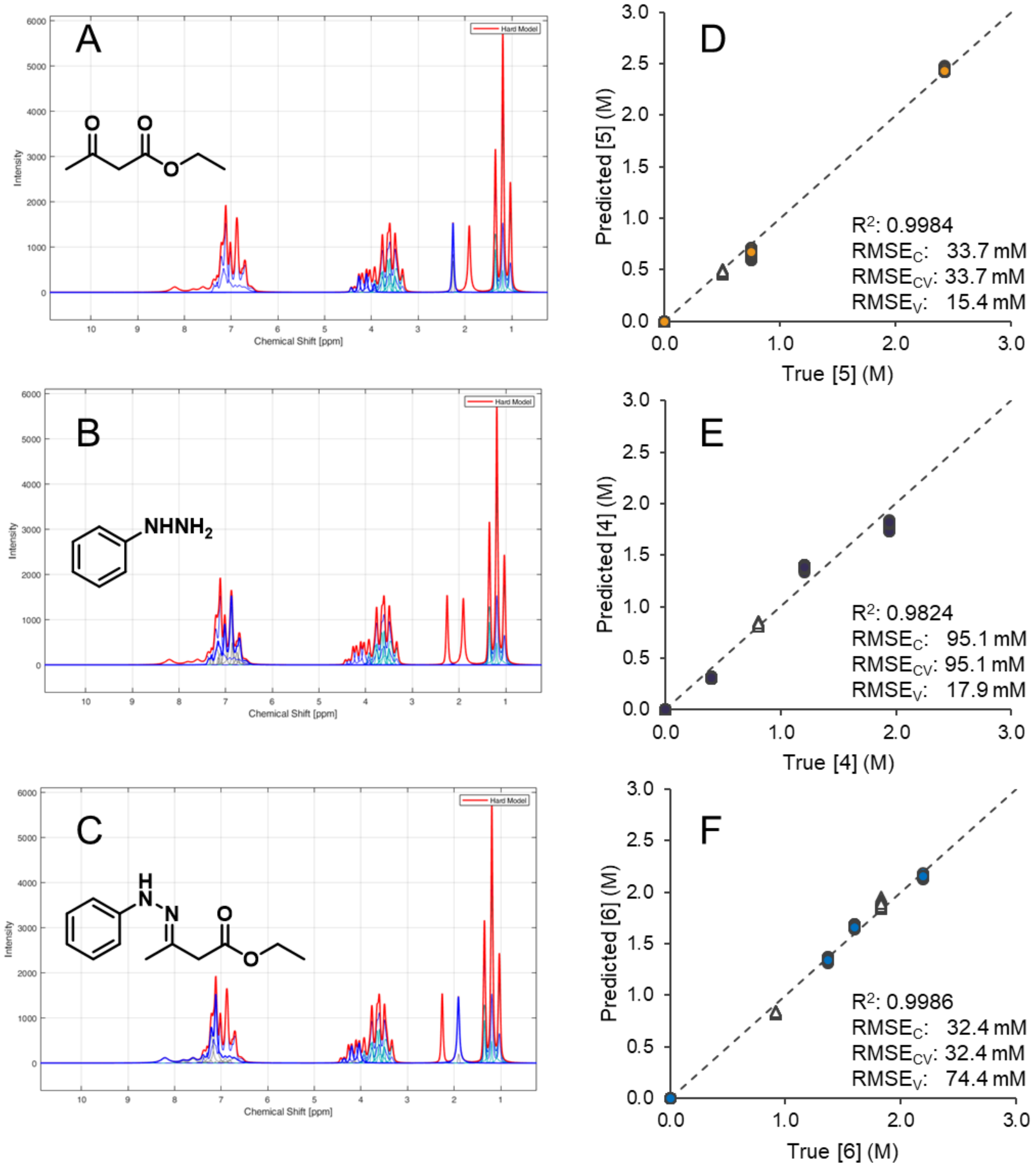

Figure S16. The mixture model is highlighted in spectra A, B and C (red). The pure component models (blue) of the ethyl acetoacetate **5**, phenylhyrdazine **4** and the imine intermediate **6** in A, B and C, respectively. Parity plots are shown in D, E and F with the target line (y = x). Colored circles show the training data, triangles show the validation data. Relevant statistical data is provided in the parity plots ($RMSE_C$: error of calibration, $RMSE_{CV}$: error of cross validation, $RMSE_V$: error of validation)

4.2 Inline FTIR

4.2.1 General Details

Inline FTIR spectra were recorded on a ReactIR 15 instrument (Mettler Toledo, ReactIR 15) equipped with a flow through cell (Mettler Toledo, Micro Flow Cell DS SiComp). The acquisition time was 15 s per data point and the spectra were recorded between 600 and 4000 cm^{-1} using a resolution of 4 cm^{-1}. It was ensured that the MCT detector was initially warmed up, then cooled with liquid N_2, the peak height between 18000 and 24000 and the signal to noise ratio was above 5000.

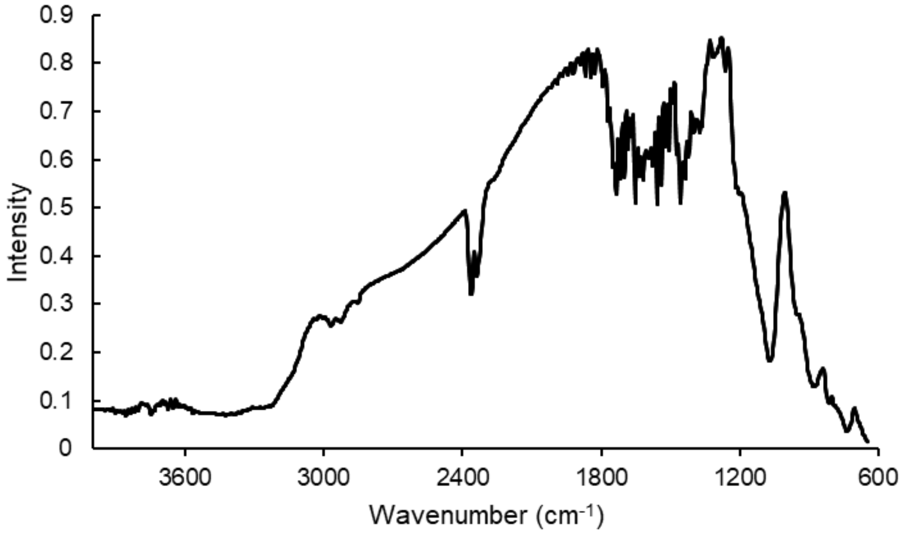

Figure S17. Background measurement from the ReactIR 15 with the Micro Flow Cell DS SiComp.

4.2.2 Process Integration (Edaravone)

The process stream was directly connected to the flow through cell, after exiting the BPR. A material test kit was used to ensure that the SiComp flow cell was compatible with the reaction stream (particularly triethylamine).

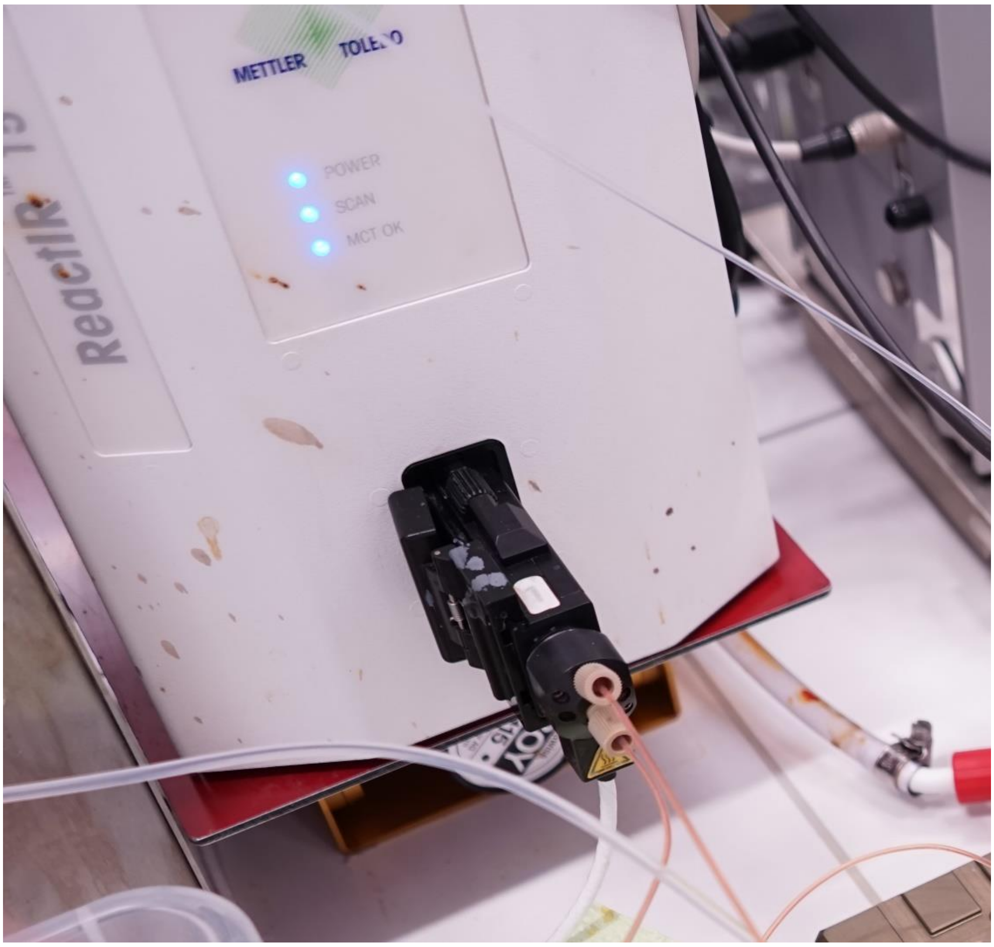

Figure S18. Photograph of the ReactIR 15 setup used for FTIR analysis.

4.2.3 Data Analysis Through Partial Least Squares (PLS) Regression (Edaravone)

Recording of training and validation data

Preparation of training and validation solutions was performed by weighing the corresponding amount of phenylhydrazine (**4**), ethyl acetoacetate (**5**), imine (**6**), triethylamine and edaravone (**7**) into either 5 or 10 mL volumetric flasks. The flasks were then filled with EtOH (96%) to the 5 or 10 mL marks and sonicated. The prepared solutions were stored at room temperature until the start of the measurement and injected with a syringe into the flow cell. Between recording different training or validation levels the flow cell was washed with EtOH to avoid cross-contamination.

Table S3. Overview of the prepared solutions for building the edaravone FTIR model.

Entry	Usage	Edaravone **7** (mol/L)	Et_3N (mol/L)	Imine **6** (mol/L)	Ethyl acetoacetate **5** (mol/L)	Phenylhydrazine **4** (mol/L)
Pure_1	Train	0	0	0	0	0
Pure_2	Train	0	0	0	2.425	0
Pure_3	Train	0	0	0	0	1.935
Pure_4	Train	0	0	1.895	0	0
Pure_5	Train	0	1.976	0	0	0
Pure_6	Train	1.374	0.747	0	0	0
Level_1	Train	0	0	0.474	2.364	0
Level_2	Train	0	0	0.947	1.183	0
Val_1	Test	0	0	0.474	0	1.866
Level_3	Train	0	0	0.947	0	0.979
Level_4	Train	0.344	0.187	0	0	2.024
Level_5	Train	0.687	0.374	0	0	1.494
Level_6	Train	0.344	0.187	0	2.441	0
Val_2	Test	0.687	0.374	0	1.205	0

Workflow for PLS regression

The acquired spectra for each model were loaded into PEAXACT, assigned labels and grouped into different levels.

Pretreatment model: All spectra underwent the same pretreatment conditions:
wavenumber range reduction to 600-1800 cm^{-1}, exclusion of solvent signal ranges (850-910 cm^{-1} and 1010-1120 cm^{-1}), rubberband baseline correction, 1st order derivative (filter length = 5).

Calibration model and validation: The training set was comprised of the pure component spectra and six different component mixtures (Level_1 to Level_6). The calibration model provided a performance indicator of model error, the root-mean-square error of calibration ($RMSE_C$). In this case, cross-validation proved to be problematic, due to the relatively small number of calibration levels available. Instead, only the errors of calibration and validation ($RMSE_C$ and $RMSE_V$, respectively) were used. To validate the model, the root-mean-square error of validation ($RMSE_V$) was calculated from the validation sets (Val_1 and Val_2). Two validation sets were required in order to cover all five species due to incompatibilities between some species.

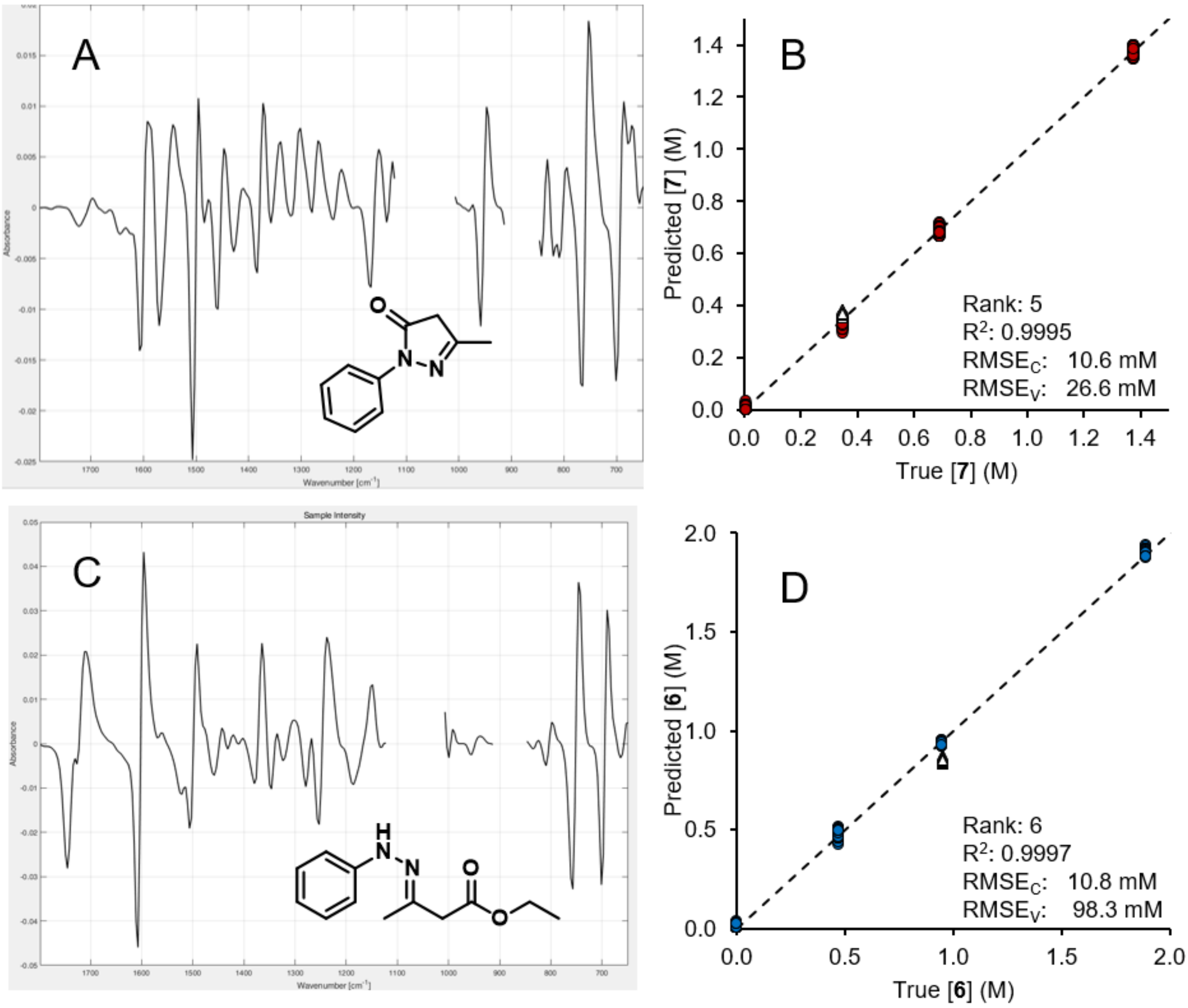

Figure S19. FTIR models for edaravone **7** and imine **6**.The individual component spectra (following preprocessing) are shown in A and C. Parity plots are shown in B and D with the target line (y = x). Colored circles show the training data, triangles show the validation data. Relevant statistical data is provided in the parity plots ($RMSE_C$: error of calibration, $RMSE_V$: error of validation).

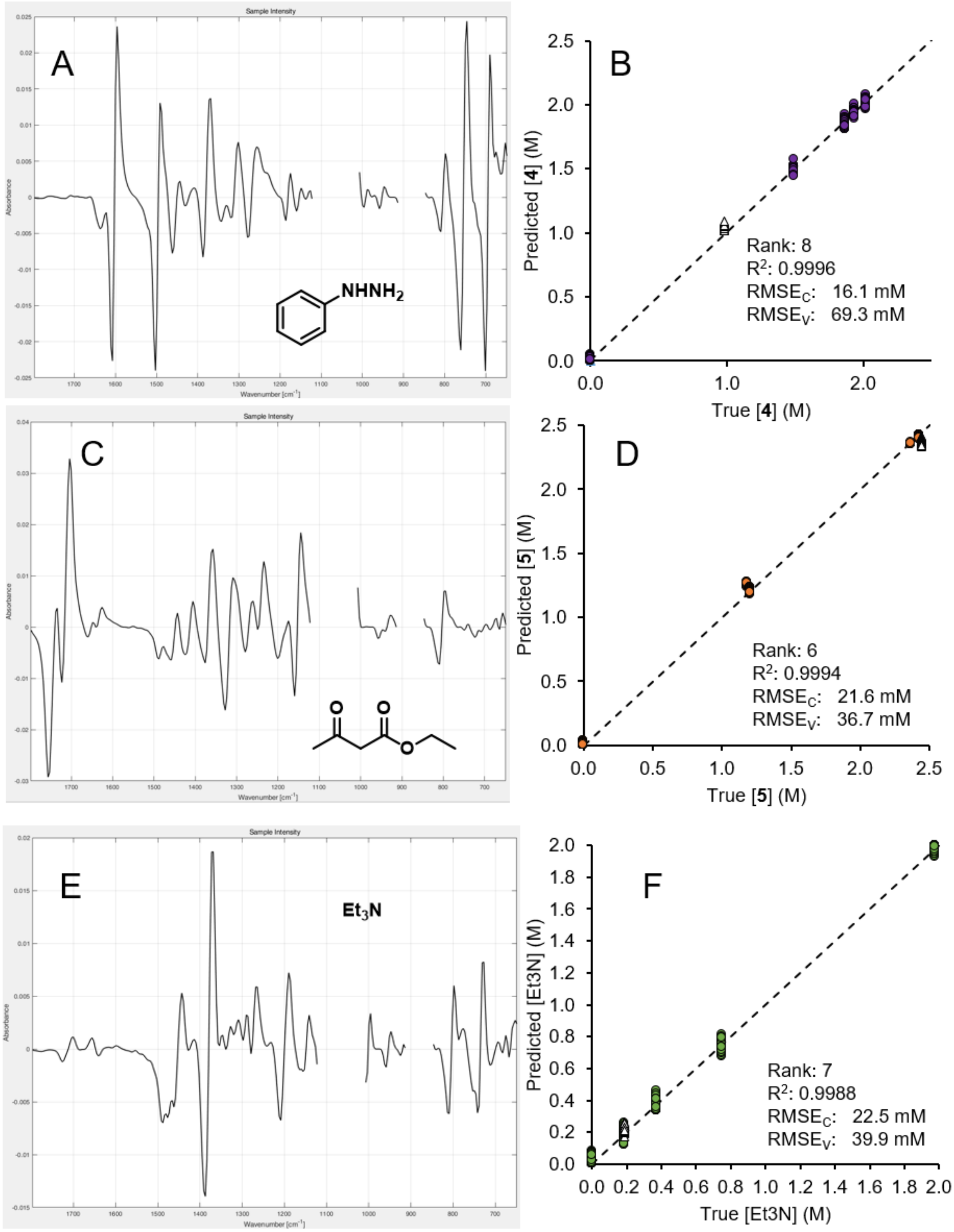

Figure S20. FTIR models for phenylhydrazine **4**, ethyl acetoacetate **5** and triethylamine. The individual component spectra (following preprocessing) are shown in A, C and E. Parity plots are shown in B, D and F with the target line (y = x). Colored circles show the training data, triangles show the validation data. Relevant statistical data is provided in the parity plots ($RMSE_C$: error of calibration, $RMSE_V$: error of validation).

5 S_NAr

5.1 Reactor Platform

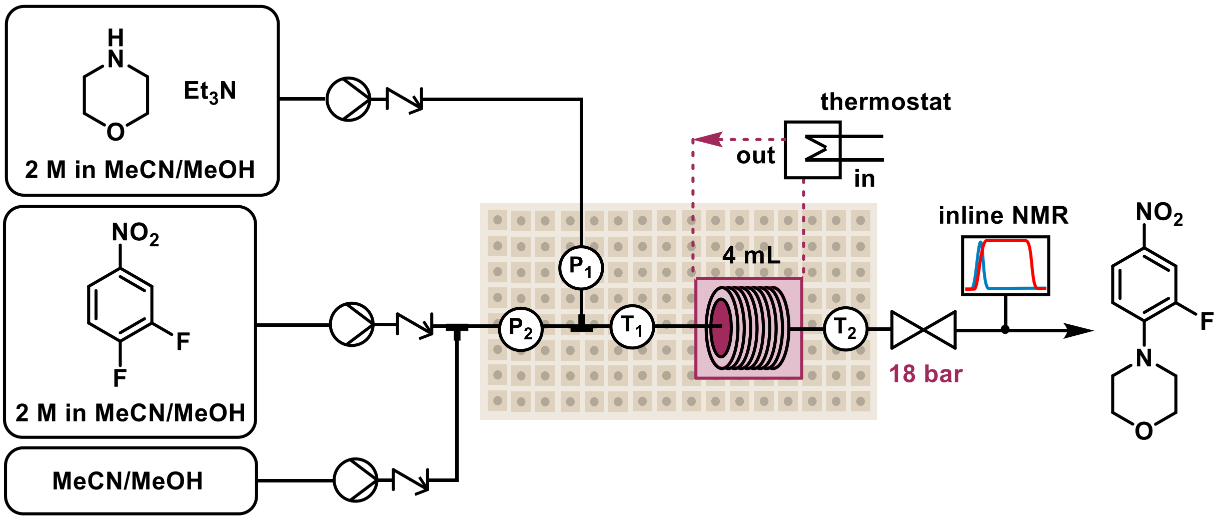

Figure S21. Detailed flow setup for the single step self-optimization of S_NAr reaction to form linezolid intermediate **3**.

Preparation of stock solutions:

2.0 M 3,4-difluoronitrobenzene solution: In a 250 mL volumetric flask 3,4-difluoronitrobenzene (79.5 g) was dissolved with a mixture of MeCN/MeOH (1+1 v/v).
2.0 M morpholine and 2.0 M triethylamine solution: In a 500 mL volumetric flask morpholine (87 g) and triethylamine (101.1 g) were diluted with a mixture of MeCN/MeOH (1+1 v/v).
Solvent mixture: In a 2 L duran bottle, HPLC grade MeCN and HPLC grade MeOH were mixed (1+1 v/v).

The S_NAr reaction was performed in a Modular MictroReaction System (Ehrfeld Mikrotechnik, MMRS). The morpholine and triethylamine stream and solvent (MeCN/MeOH) stream was delivered with two SyrDos2 pumps (90 bar valve, 1.0 mL syringes). The two streams were mixed prior to the MMRS system in a T-piece (PEEK, 0.5 mm i.d.) and entered the MMRS system through a 1/16" in/out connector (0711-2-0124-F, Hastelloy C-276), followed by a pressure sensor module (0518-1-60x4-F, Hastelloy C-276). The substrate feed with 3,4-difluoronitrobenzene was delivered with a SyrDos2 pump (90 bar valve, 1.0 mL syringes) through PFA tubing to the MMRS system. The feed entered the system through a 1/16" in/out connector (0711-2-0124-F, Hastelloy C-276), followed by a pressure sensor module (0518-1-60x4-F, Hastelloy C-276) and was mixed in a T-type

connecting module (0723-1-0004, Hastelloy C-276) with the diluted morpholine stream. The reaction mixture passed through a temperature sensor (0501-2-1004-X, Hastelloy C-276), followed by a capillary reactor (0214-1-1004-F, build in connection body of 4.00 mL, Hastelloy C-276) which was temperature controlled by a thermostat (Huber, Ministat 240). After the capillary reactor, the reaction solution passed through another temperature sensor (0501-2-1004-X, Hastelloy C-276) and exited the MMRS system via a 1/16" in/out connector (0711-2-0124-F, Hastelloy C-276). The outlet PFA tubing (0.2 mL, 0.8 mm i.d.) from the MMRS system was placed in a water bath and connected to a membrane based BPR (Zaiput, BPR-10) which was set to 18 bar. The reaction stream was delivered through PFA tubing (0.9 mL, 0.8 mm i.d.) to the benchtop NMR (Magritek, Spinsolve 43 Ulta). A 6-port valve was installed prior to the glass flow cell (800 μL internal volume, 550 mm length) by-passing the NMR in case of re-shimming the instrument (see section 4.1.2). The reaction stream left the NMR through PFA tubing (0.3 mL, 0.8 mm i.d.) and was collected in the receiver vessel.

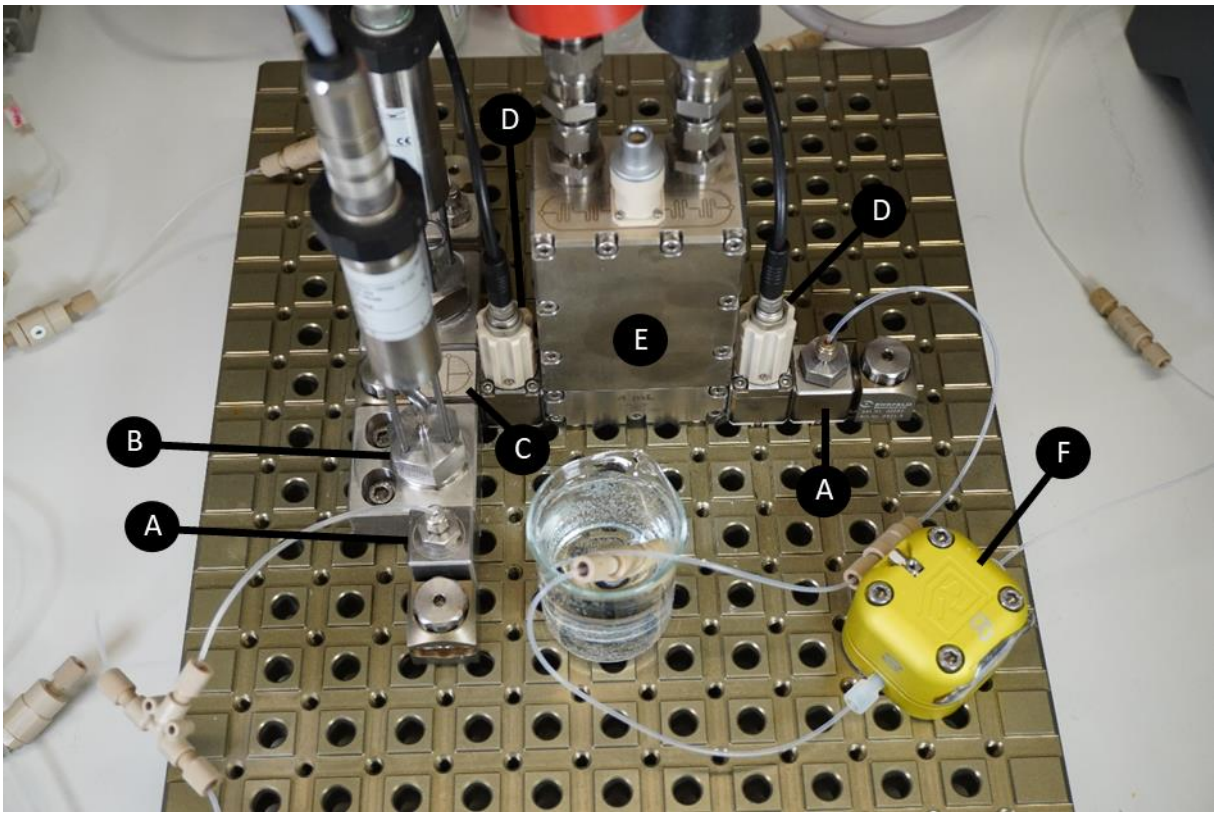

Figure S22. Image of the first S_NAr reaction setup in the MMRS system. (A) in/out connector, (B) pressure sensor module, (C) T-type connecting module, (D) temperature sensor module, (E) capillary reactor, (F) Zaiput BPR

5.2 Reactor Inputs and Reactor Outputs

During the self-optimization experiment there are reactor inputs which are fixed and some which are manipulated. Additionally, there are reactor inputs which depend on the manipulated inputs (**Figure S23**). Reactor outputs could be either measured directly from the NMR or calculated from the measured outputs.

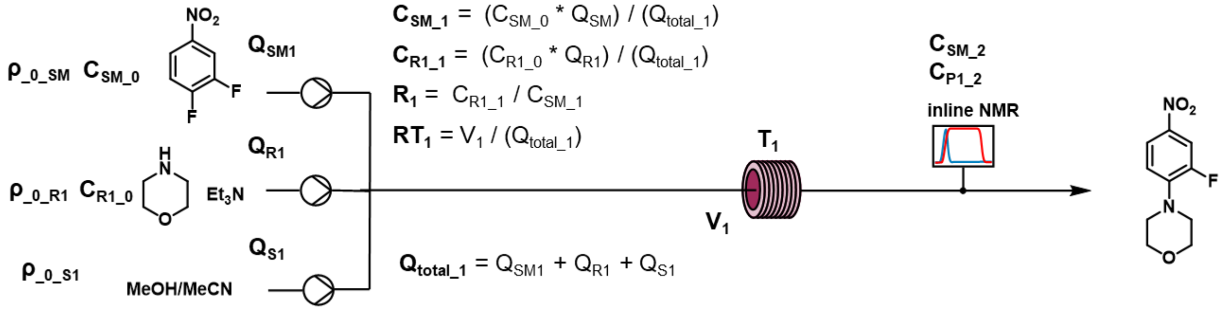

Figure S23. Schematic overview of reactor inputs and outputs for the S_NAr self-optimization experiments.

The following variables (reactor inputs) were fixed during a self-optimization experiment:

$\mathbf{C_{SM\_0}}$: initial concentration of starting material

$\mathbf{C_{R1\_0}}$: initial concentration of reagent 1

$\mathbf{V_1}$: reactor volume for the reaction step

$\boldsymbol{\rho_{\_0\_SM}}$: density of starting material solution

$\boldsymbol{\rho_{\_0\_R1}}$: density of reagent 1 solution

$\boldsymbol{\rho_{\_0\_S1}}$: density of solvent 1 solution

The following variables (reactor inputs) were manipulated during a self-optimization experiment:

$\mathbf{Q_{SM1}}$: volume flow of starting material

$\mathbf{Q_{R1}}$: volume flow of reagent 1

$\mathbf{Q_{S1}}$: volume flow of solvent 1

$\mathbf{T_1}$: temperature of the reactor

The following variables (reactor inputs) were depending on the manipulated inputs during a self-optimization experiment:

$\mathbf{C_{SM\_1}}$: concentration of starting material

$\mathbf{C_{R1\_1}}$: concentration of reagent 1

$\mathbf{R_1}$: ratio of reagent 1 to starting material

RT_1: residence time (time of material in the reactor "reaction time")

The following variables (reactor outputs) were measured during a self-optimization experiment.

C_{SM_2}: concentration of starting material

C_{P1_2}: concentration of product 1

Q_{total_1}: total volume flow for first step

The following variables (reactor outputs) were calculated for the measured outputs during a self-optimization experiment.

Throughput: $TP\_1 = C_{P1\_2} * Q_{total\_1}$

Conversion step 1: $Conv\_1 = 1 – (C_{SM\_2} / C_{SM\_1})$

Yield step 1: $Yield\_1 = C_{P1\_2} / ((C_{SM\_0} * Q_{SM\_1}) / Q_{total\_1})$

Selectivity step 1: Selectivity_1 = Yield_1 / Conv_1

Space-time yield step 1: $STY\_1 = (C_{P1\_2} * Q_{total\_1} / V1 * M_{P1} / 1000 * 60)$

E-Factor step 1: $E\_Factor\_1 = (((\rho_{\_0\_SM} * Q_{SM}) + (\rho_{\_0\_R1} * Q_{R1}) + (\rho_{\_0\_S1} * Q_{S1})) / (C_{P1\_2} * M_{P1} * Q_{total\_1} / 1000)) – 1$

5.3 Optimization Parameters

In the self-optimization experiments of the S_NAr reaction, the optimization algorithm could adjust four different variables: ratio of reagent 1 (morpholine) to starting material (3,4-difluoronitrobenzene), concentration of starting material, residence time in the reactor and the temperature of the reactor. The adjustable variables had the following upper and lower bounds (**Table S4**). The objective of the optimization was to simultaneously maximize the space-time yield (STY) and conversion in the reaction, as defined by Equation (1). In the multi-objective optimization, the STY and conversion were maximized and the E-factor minimized, according to Equation (2)

Table S4. Lower and upper bounds for the four adjustable variables in the self-optimization experiments for the S_NAr reaction.

Limits	R_1 / SM	Conc. SM (mol/L)	RT (min)	Temp 1 (°C)
Lower	0.9	0.2	2.5	60
Upper	3.0	0.4	6.0	160

$$\text{minimize}\ [-\ln\ (\text{STY}), -\ln\ (\text{Conversion})] \quad (1)$$

$$\text{minimize}\left[-\ln(\text{STY}), -\ln(\text{Conversion}), \ln(\text{E}-\text{factor})\right] \quad (2)$$

5.4 Reaction Optimization (S_NAr)

5.4.1 Batch Experiments

4-(2-fluoro-4-nitrophenyl)morpholine (3)

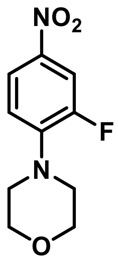

To a solution of 3,4-difluoronitrobenzene (15.0 g, 94.3 mmol) in DMAc (60 mL) was added morpholine (9.04 g, 104 mmol, 1.1 equiv). The solution was cooled to 0 °C and DBU (15.8 g, 104 mmol, 1.1 equiv) was slowly added under vigorous stirring. The solution was warmed and stirred for 1 h at room temperature, after which the mixture was diluted with water (500 mL). A yellow precipitate was filtered off and the residue was washed with water (3 × 100 mL) and petroleum ether (2 × 100 mL). The resulting yellow solids were dried in a vacuum oven for approximately 4 h at 80 °C, providing the pure product (18.6 g, 87%).

^{1}H NMR (^{1}H NMR (300 MHz, $CDCl_3$) δ 7.92 (dd, J = 9.0, 2.7 Hz, 1H), 7.84 (dd, J = 13.1, 2.6 Hz, 1H), 6.85 (t, J = 8.8 Hz, 1H), 3.85 – 3.75 (m, 4H), 3.25 – 3.18 (m, 4H).

^{13}C NMR (75 MHz, $CDCl_3$) δ 153.3 (d, *J* = 249.6 Hz), 145.6 (d, *J* = 7.6 Hz), 140.9 (d, *J* = 8.8 Hz), 121.1 (d, *J* = 3.0 Hz), 117.0 (d, *J* = 4.0 Hz), 112.7 (d, *J* = 26.3 Hz), 66.7, 50.0 (d, *J* = 4.9 Hz).

LCMS (m/z): 227 [M+H], 268 [M+MeCN+H]

5.4.2 Self-Optimization (LHC)

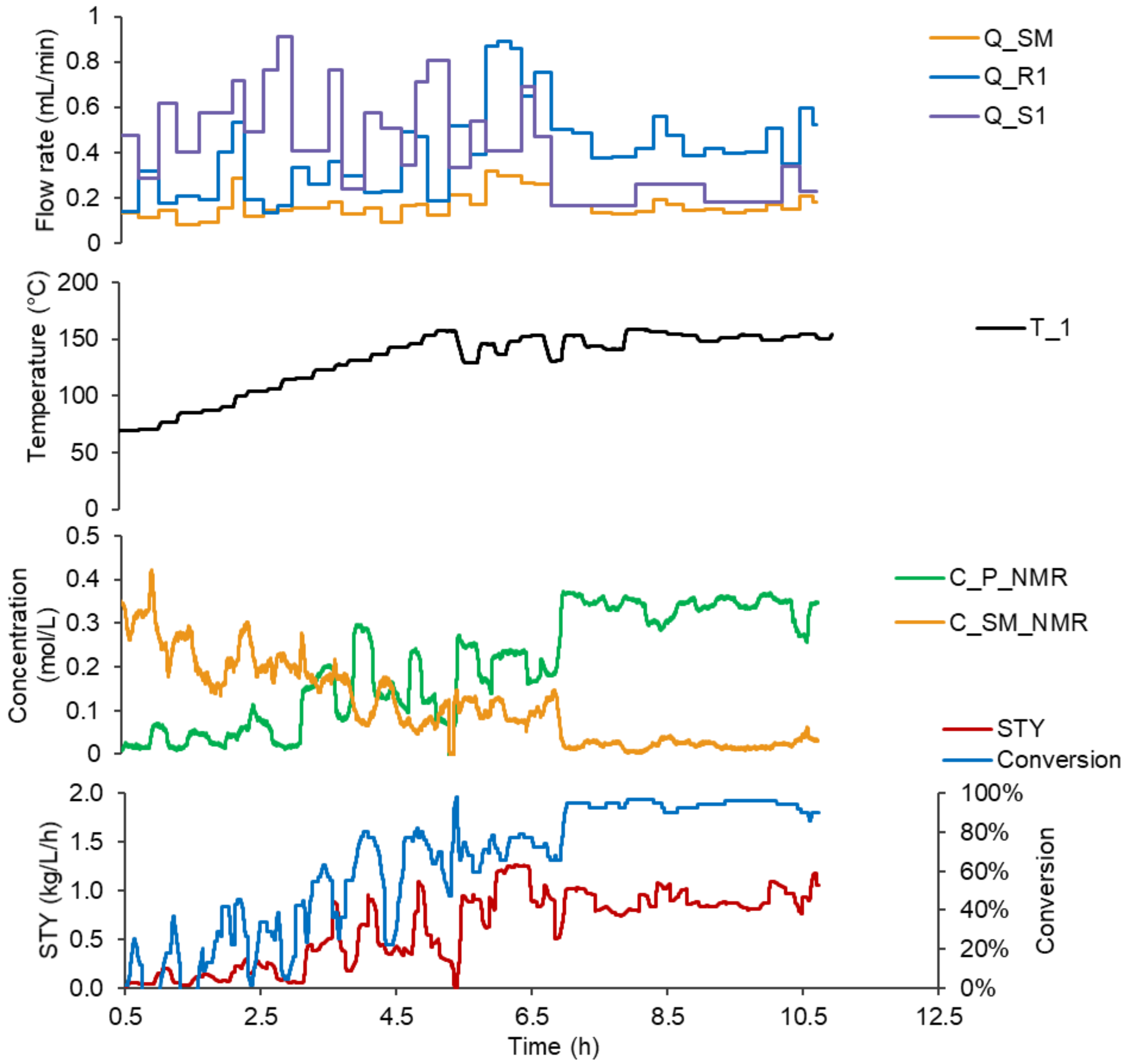

Figure S24. Real-time data from the S_NAr self-optimization experiment using a LHC as initial data set.

Table S5. Hyperparameters resulting from the GPs generated in the experiment using LHC as initial data set. The lower the hyperparameter for a variable, the higher its influence on the optimization objective.

Variable	STY	Conversion
$\theta_{R\_1/SM}$	3.2453	6.4862
θ_{Conc}	44.8582	94.8807
θ_{RT}	8.6003	7.3272
θ_{Temp}	0.0375	0.0273

Table S6. Results from the self-optimization experiment using a LHC design as initial data set. The adjusted variables were the ratio of reagent 1 (morpholine) to starting material (3,4-difluoronitrobenzene), concentration of starting material, residence time in the reactor and the temperature of the reactor. The objectives were space-time yield (STY) and conversion.

Entry	R_1 / SM	Conc. SM (mol/L)	RT (min)	Temp (°C)	STY (kg/L/h)	Conversion
LHC_1	1.82	0.366	2.77	60.3	0.02	2.9%
LHC _2	1.05	0.357	5.38	69.4	0.04	10.0%
LHC _3	2.85	0.312	5.58	70.8	0.16	8.2%
LHC _4	1.25	0.304	4.28	76.7	0.04	13.1%
LHC _5	2.47	0.241	5.82	84.7	0.11	20.7%
LHC _6	2.14	0.210	4.67	87.6	0.06	25.3%
LHC _7	2.63	0.269	3.53	90.2	0.16	21.0%
LHC _8	1.85	0.374	2.61	99.9	0.31	24.8%
LHC _9	1.59	0.299	5.02	104.3	0.19	33.2%
LHC _10	0.90	0.281	3.84	106.2	0.08	24.5%
LHC _11	1.14	0.238	3.27	114.4	0.07	17.2%
LHC _12	2.16	0.343	4.49	115.9	0.45	47.9%
LHC _13	1.69	0.396	5.17	122.8	0.52	55.3%
LHC _14	1.98	0.279	3.05	127.5	0.37	37.5%
LHC _15	2.30	0.387	5.98	131.0	0.63	81.0%
LHC _16	1.42	0.328	4.22	136.8	0.43	50.0%
LHC _17	2.54	0.218	4.87	143.1	0.33	62.7%
LHC _18	2.97	0.330	3.99	145.8	0.80	83.0%
LHC _19	2.78	0.250	2.97	153.5	0.54	70.1%
LHC _20	1.50	0.221	3.59	157.1	0.26	46.0%
Self_1	2.45	0.398	3.75	129.7	0.89	68.4%
Self_2	2.31	0.308	3.63	145.5	0.63	69.1%
Self_3	2.72	0.400	2.50	136.7	1.21	68.3%
Self_4	3.00	0.372	2.51	148.0	1.25	78.6%
Self_5	2.89	0.372	2.51	152.6	1.24	79.8%
Self_6	2.47	0.328	2.50	153.1	0.89	70.7%
Self_7	2.90	0.350	2.70	130.9	0.90	63.7%
Self_8	3.00	0.400	4.79	153.0	1.02	96.4%
Self_9	2.93	0.395	4.75	143.6	0.96	92.8%
Self_10	2.88	0.374	5.70	141.3	0.78	93.3%
Self_11	2.95	0.380	5.88	158.2	0.78	98.3%
Self_12	2.94	0.346	4.89	156.6	0.84	93.5%
Self_13	2.93	0.366	3.82	154.6	1.07	89.8%
Self_14	2.76	0.392	4.56	153.8	1.02	93.2%
Self_15	2.70	0.379	5.30	148.3	0.85	93.0%
Self_16	2.78	0.399	5.35	150.9	0.89	96.3%
Self_17	3.00	0.360	5.42	153.8	0.82	96.1%
Self_18	2.82	0.396	5.55	149.4	0.88	96.2%
Self_19	2.94	0.388	4.51	152.1	1.04	94.1%
Self_20	2.36	0.355	4.77	154.4	0.76	90.1%
Self_21	2.89	0.400	3.90	150.7	1.18	91.6%

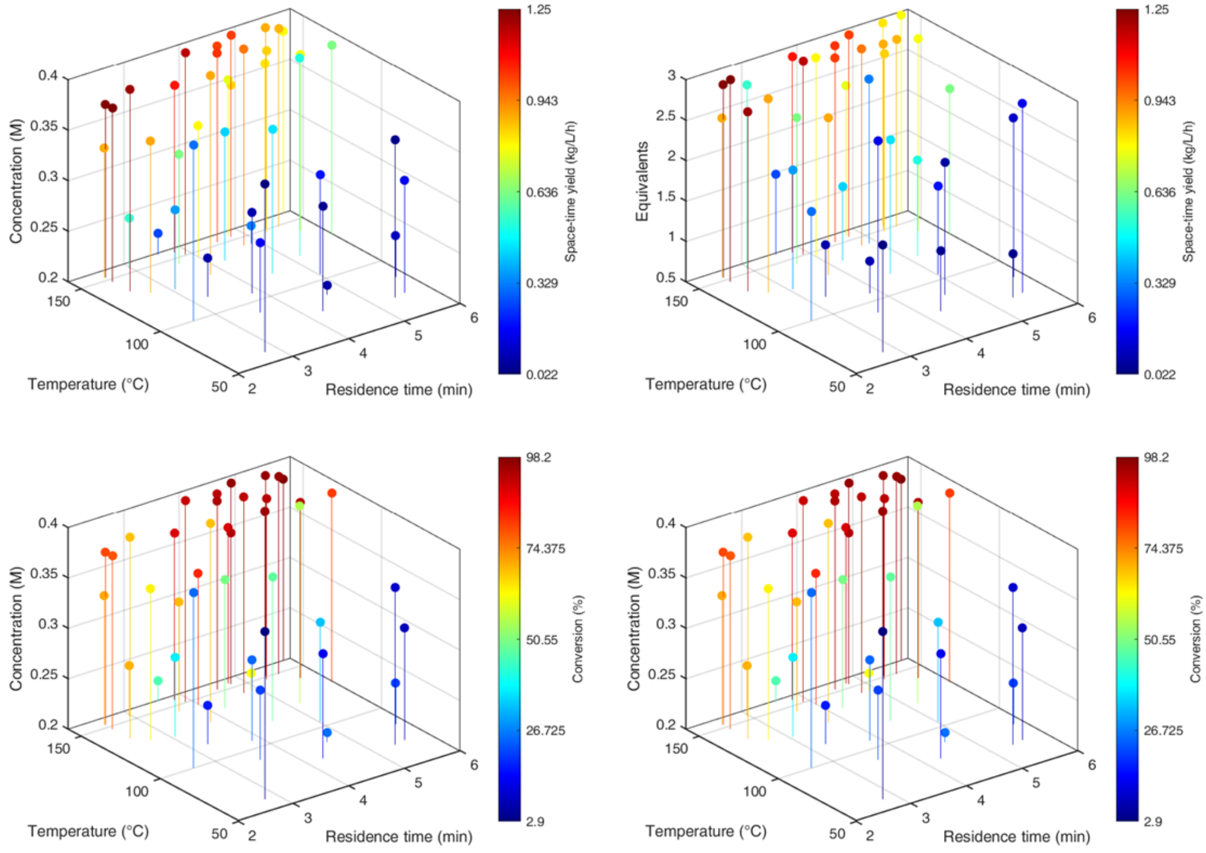

Figure S25. Optimization plots for the S_NAr reaction using a LHC as initial data set.

5.4.3 Self-Optimization (Restricted full factorial DoE)

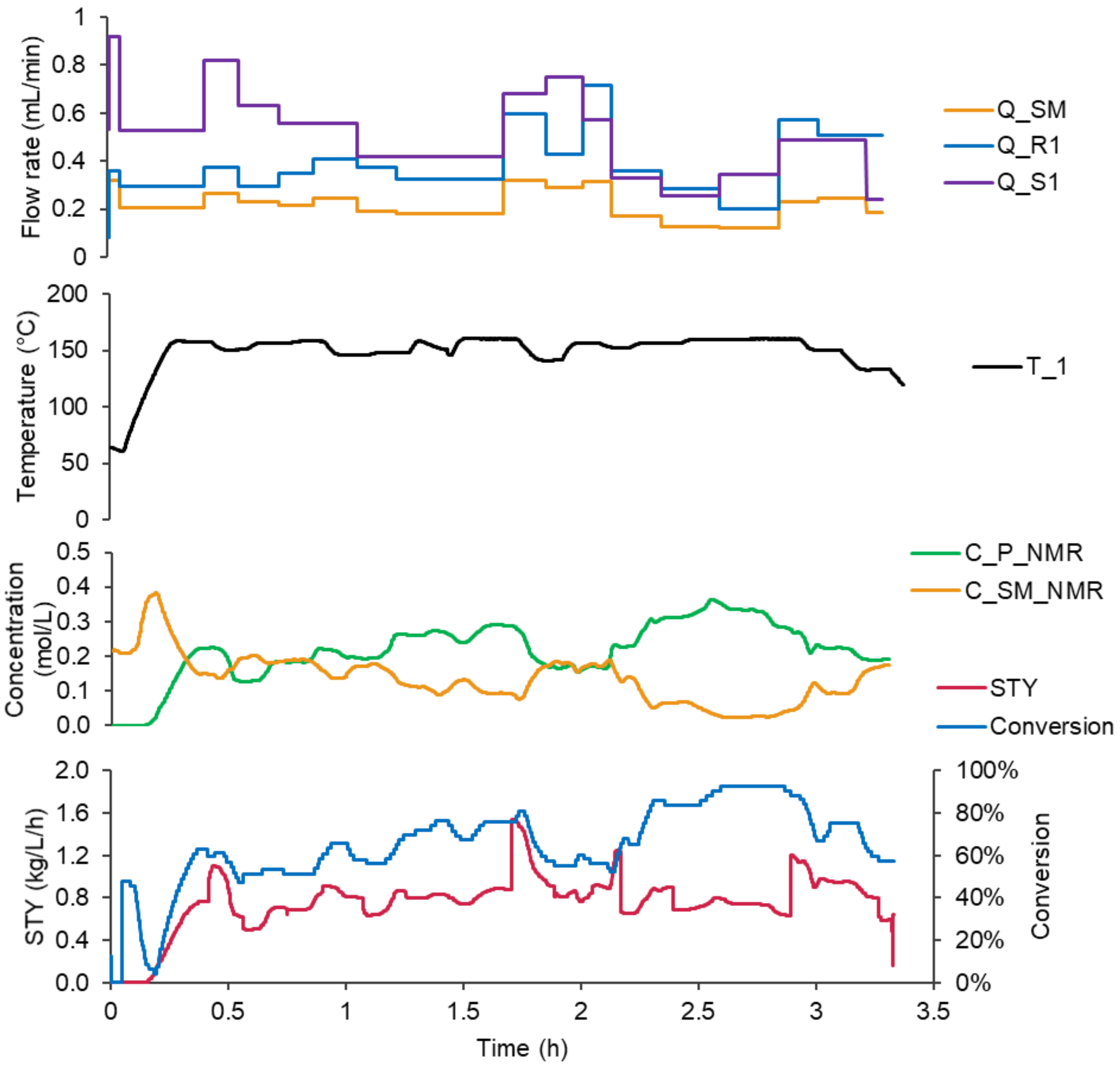

Figure S26. Real-time data from the S_NAr self-optimization experiment using a restricted full factorial DoE as initial data set.

Table S7. Hyperparameters resulting from the GPs generated in the experiment using restricted full factorial DoE as initial data set. The lower the hyperparameter for a variable, the higher its influence on the optimization objective.

Variable	STY	Conversion
$\theta_{R\_1/SM}$	2.3102	0.9048
θ_{Conc}	30.0476	36.3219
θ_{RT}	9.0351	1.6472
θ_{Temp}	0.0277	0.0145

Table S8. Results from the self-optimization experiment using a restricted full factorial design as initial data set. The adjusted variables were the ratio of reagent 1 (morpholine) to starting material (3,4-difluoronitrobenzene), concentration of starting material, residence time in the reactor and the temperature of the reactor. The objectives were space-time yield (STY) and conversion.

Entry	R_1 / SM	Conc. SM (mol/L)	RT (min)	Temp (°C)	STY (kg/L/h)	Conversion
DoE_1	1.11	0.200	2.50	60.0	0.01	3.5%
DoE_2	1.11	0.200	6.00	60.0	0.01	2.0%
DoE_3	0.50	0.200	2.50	60.0	0.01	4.3%
DoE_4	0.50	0.200	6.00	60.0	0.00	1.1%
DoE_5	1.11	0.400	2.50	60.0	0.03	8.1%
DoE_6	1.11	0.400	6.00	60.0	0.05	3.5%
DoE_7	0.50	0.400	2.50	60.0	0.02	2.6%
DoE_8	0.50	0.400	6.00	60.0	0.02	0.1%
DoE_9	0.69	0.300	4.25	105.0	0.08	8.1%
DoE_10	0.69	0.300	4.25	105.0	0.07	9.0%
DoE_11	1.11	0.200	2.50	150.0	0.12	25.5%
DoE_12	1.11	0.200	6.00	150.0	0.13	39.2%
DoE_13	0.50	0.200	2.50	150.0	0.05	15.6%
DoE_14	0.50	0.200	6.00	150.0	0.05	23.5%
DoE_15	1.11	0.400	2.50	150.0	0.58	42.1%
DoE_16	1.11	0.400	6.00	150.0	0.42	58.2%
DoE_17	0.50	0.400	2.50	150.0	0.27	21.8%
DoE_18	0.50	0.400	6.00	150.0	0.20	25.7%
DoE_19	0.69	0.300	4.25	105.0	0.07	9.6%
Self_1	1.44	0.400	3.89	157.3	0.76	63.3%
Self_2	1.42	0.362	2.74	150.6	0.62	46.1%
Self_3	1.28	0.400	3.45	156.2	0.72	53.6%
Self_4	1.64	0.382	3.57	157.9	0.83	56.1%
Self_5	1.65	0.400	3.24	146.0	0.80	57.6%
Self_6	1.94	0.390	4.07	147.9	0.86	69.5%
Self_7	1.79	0.397	4.40	160.0	0.88	76.9%
Self_8	1.87	0.400	2.50	141.3	0.91	55.6%
Self_9	1.46	0.397	2.72	155.9	0.84	55.8%
Self_10	2.27	0.393	2.50	152.5	1.23	64.5%
Self_11	2.12	0.395	4.64	156.1	0.90	83.8%
Self_12	2.24	0.382	6.00	159.4	0.75	94.3%
Self_13	1.65	0.365	6.00	160.0	0.63	89.0%
Self_14	2.49	0.356	3.11	150.2	0.95	73.2%
Self_15	2.05	0.390	3.15	133.0	0.80	57.6%

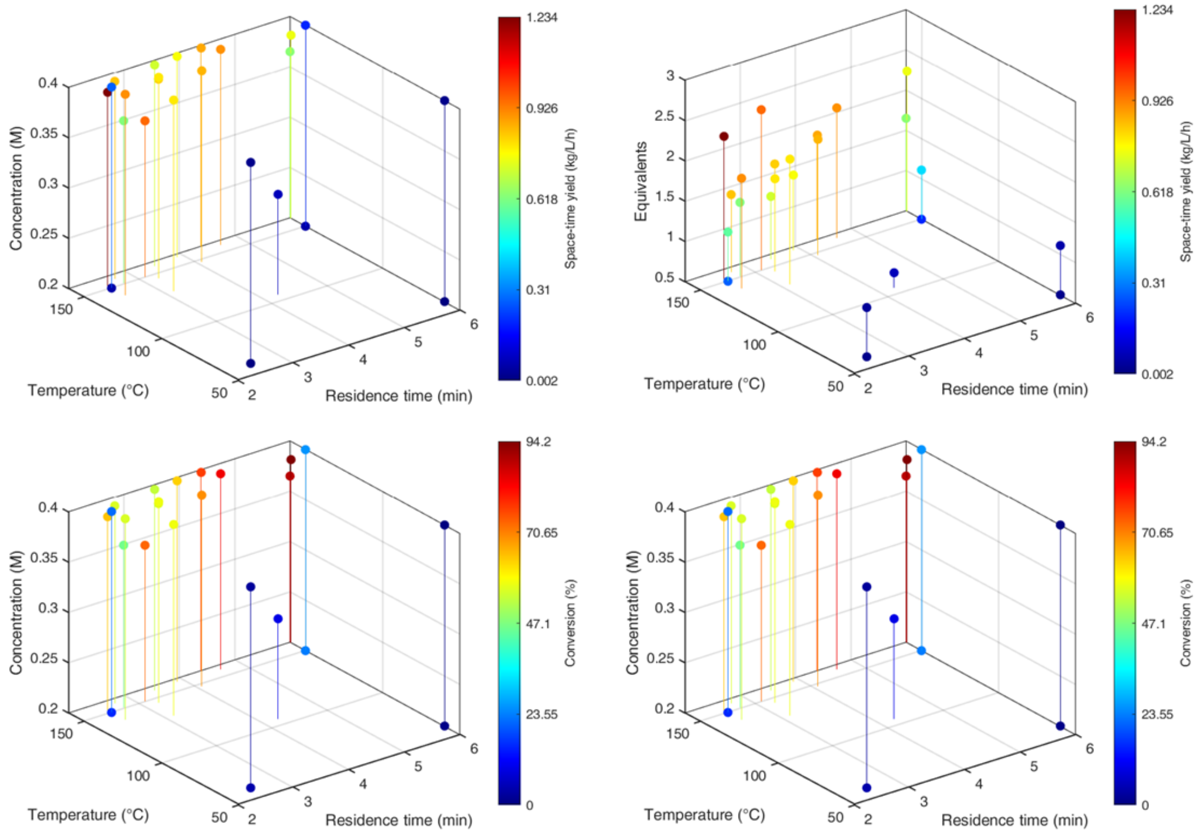

Figure S27. Optimization plots for the S_NAr reaction using a restricted DoE as initial data set.

5.4.4 Self-Optimization (Full factorial DoE)

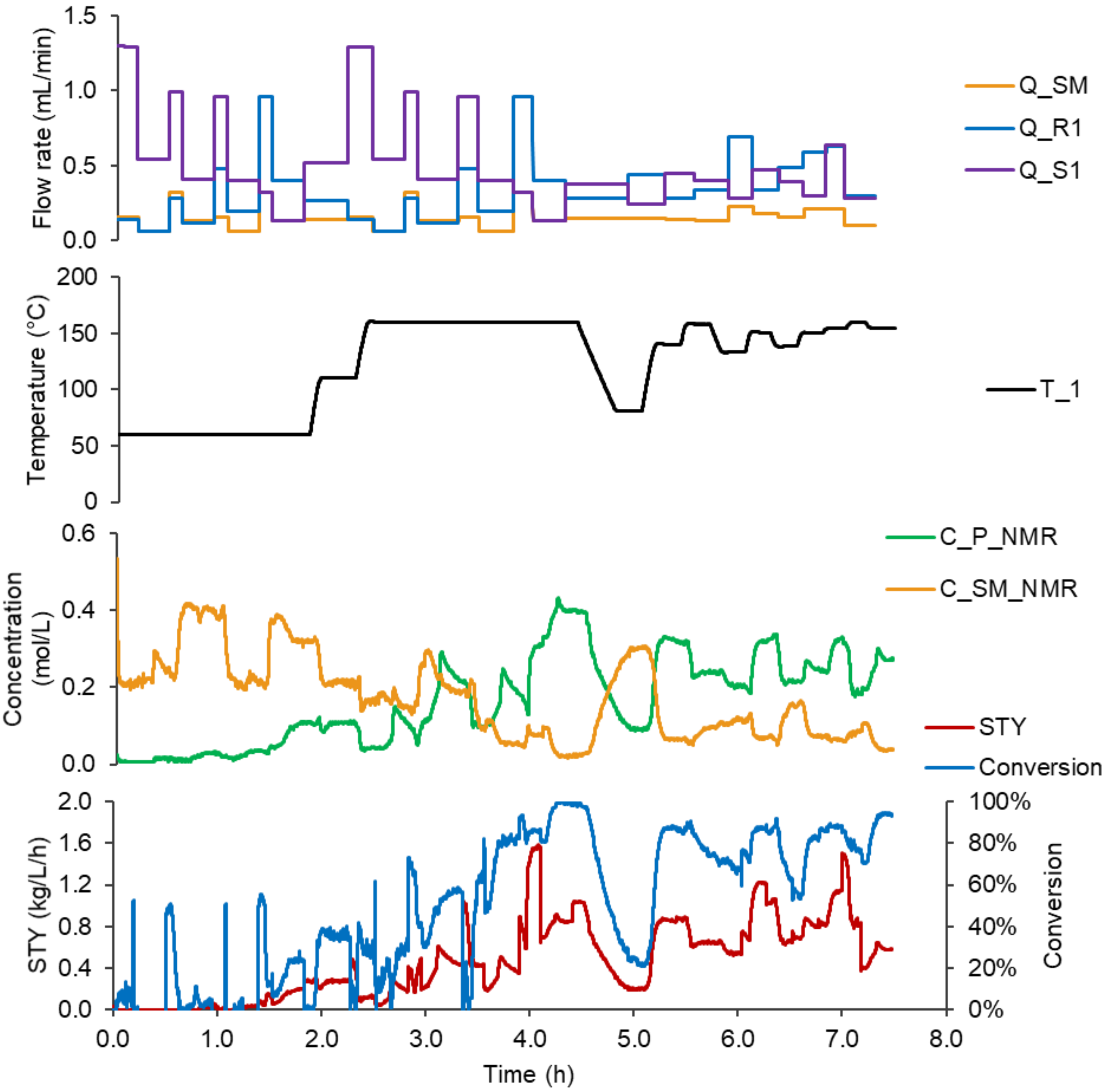

Figure S28. Real-time data from the S_NAr self-optimization experiment using a full factorial DoE as initial data set.

Table S9. Hyperparameters resulting from the GPs generated in the experiment using a full factorial DoE as initial data set. The lower the hyperparameter for a variable, the higher its influence on the optimization objective.

Variable	STY	Conversion
$\theta_{R\_1/SM}$	1.9107	0.6846
θ_{Conc}	21.6386	8.3102
θ_{RT}	1.7382	0.4449
θ_{Temp}	0.0190	0.0093

Table S10. Results from the self-optimization experiment using a full factorial design as initial data set. The adjusted variables were the ratio of reagent 1 (morpholine) to starting material (3,4-difluoronitrobenzene), concentration of starting material, residence time in the reactor and the temperature of the reactor. The objectives were space-time yield (STY) and conversion.

Entry	R_1 / SM	Conc. SM (mol/L)	RT (min)	Temp (°C)	STY (kg/L/h)	Conversion
DoE_1	0.90	0.200	2.50	60.0	0.00	28.1%
DoE_2	0.90	0.200	6.00	60.0	0.00	0.0%
DoE_3	0.90	0.400	2.50	60.0	0.00	14.1%
DoE_4	0.90	0.400	6.00	60.0	0.02	5.2%
DoE_5	3.00	0.200	2.50	60.0	0.00	0.0%
DoE_6	3.00	0.200	6.00	60.0	0.04	2.6%
DoE_7	3.00	0.400	2.50	60.0	0.14	12.6%
DoE_8	3.00	0.400	6.00	60.0	0.20	25.2%
DoE_9	1.95	0.300	4.25	110.0	0.28	35.0%
DoE_10	1.95	0.300	4.25	110.0	0.28	35.4%
DoE_11	1.95	0.300	4.25	110.0	0.28	34.9%
DoE_12	0.90	0.200	2.50	160.0	0.12	25.6%
DoE_13	0.90	0.200	6.00	160.0	0.19	36.4%
DoE_14	0.90	0.400	2.50	160.0	0.42	40.7%
DoE_15	0.90	0.400	6.00	160.0	0.44	57.5%
DoE_16	3.00	0.200	2.50	160.0	0.42	62.7%
DoE_17	3.00	0.200	6.00	160.0	0.36	84.5%
DoE_18	3.00	0.400	2.50	160.0	1.57	85.8%
DoE_19	3.00	0.400	6.00	160.0	0.85	99.8%
Self_1	1.82	0.379	4.82	81.2	0.20	26.1%
Self_2	2.87	0.367	4.69	140.2	0.85	87.8%
Self_3	1.96	0.332	4.51	158.2	0.65	78.5%
Self_4	2.59	0.301	4.49	133.3	0.54	67.5%
Self_5	3.00	0.384	3.30	150.8	1.22	87.3%
Self_6	1.90	0.364	3.96	138.4	0.66	62.9%
Self_7	2.92	0.321	3.81	150.4	0.80	83.6%
Self_8	2.76	0.388	3.61	154.4	1.14	88.5%
Self_9	2.93	0.289	2.68	160.0	0.82	77.2%
Self_10	2.96	0.298	5.82	154.9	0.58	93.4%
Self_11	2.21	0.231	2.83	154.3	0.41	52.3%
Self_12	2.68	0.219	3.37	159.5	0.46	63.8%
Self_13	2.03	0.360	4.82	154.2	0.71	81.6%
Self_14	1.95	0.396	3.87	152.0	0.89	75.3%
Self_15	2.98	0.379	2.89	123.4	0.96	61.7%
Self_16	2.28	0.398	4.11	108.6	0.59	50.6%
Self_17	2.96	0.399	3.20	95.6	0.59	41.7%
Self_18	1.35	0.351	2.93	154.7	0.61	51.6%
Self_19	2.56	0.390	2.55	112.5	0.73	44.7%
Self_20	2.72	0.383	2.66	157.6	1.29	82.6%
Self_21	2.51	0.296	2.89	160.0	0.76	73.8%
Self_22	2.92	0.400	4.07	146.8	1.09	90.4%
Self_23	2.65	0.340	3.10	127.2	0.70	59.7%
Self_24	2.95	0.288	4.99	114.9	0.40	61.1%
Self_25	2.97	0.400	3.65	132.4	1.04	80.6%
Self_26	2.79	0.364	3.91	160.0	1.01	89.6%

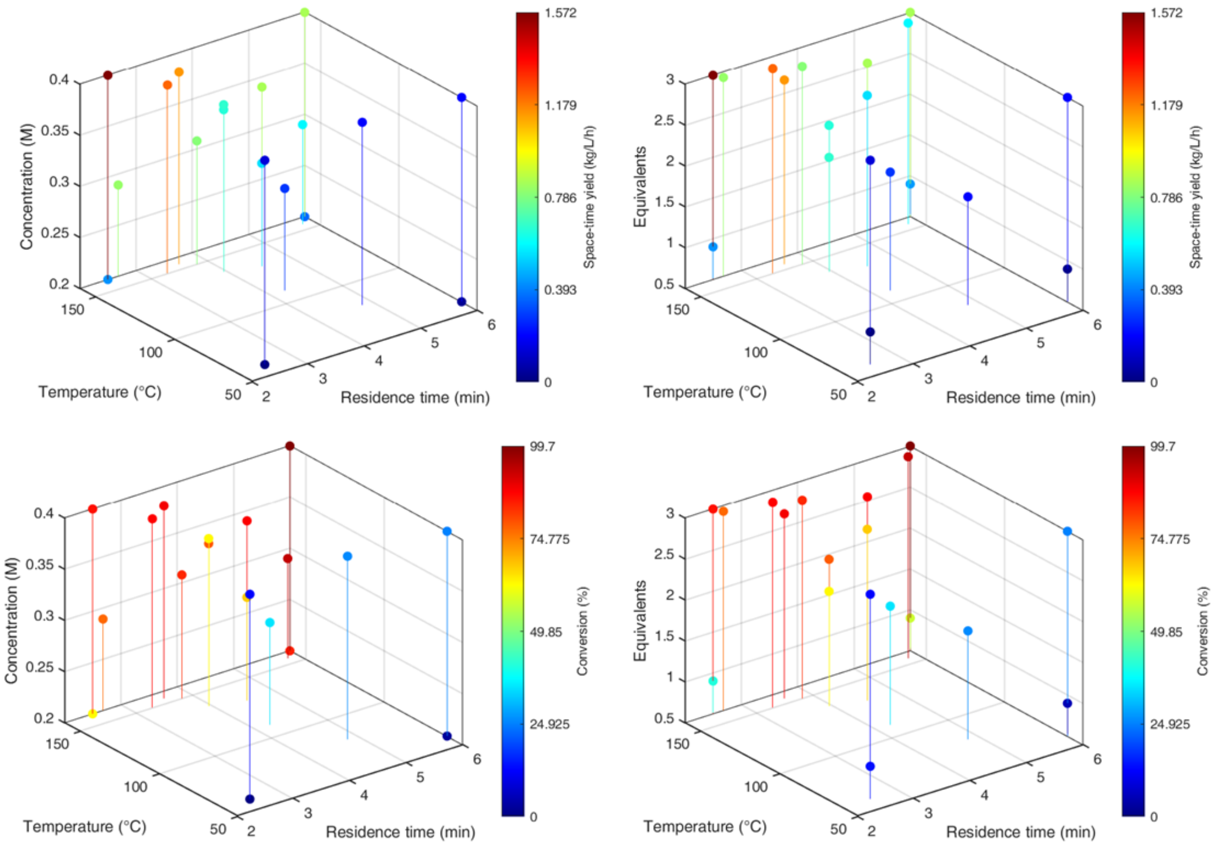

Figure S29. Optimization plots for the S_NAr reaction using a full factorial DoE as initial data set.

5.4.5 Self-Optimization (“center points”)

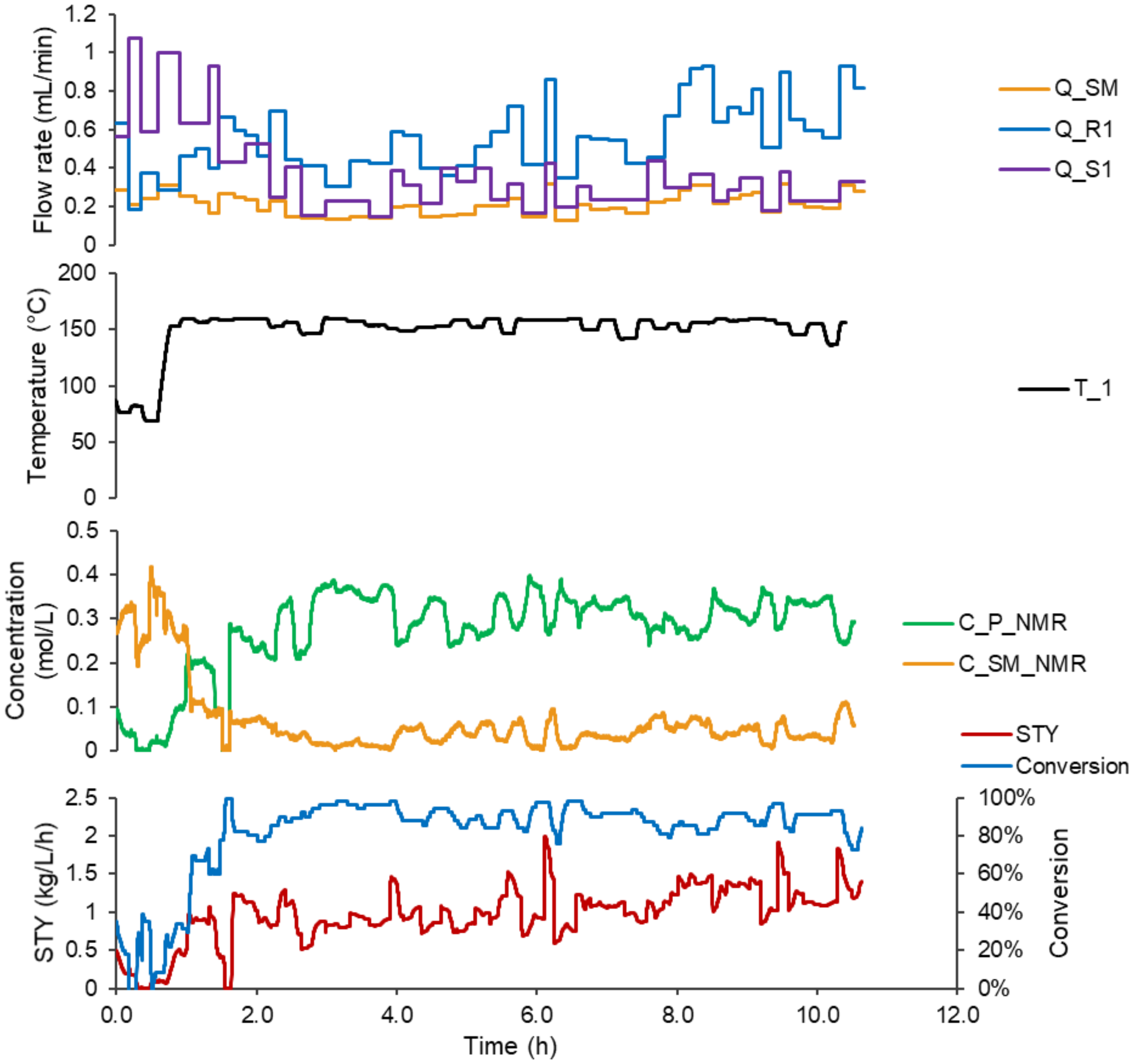

Figure S30. Real-time data from the S_NAr self-optimization experiment using centerpoints (1) as initial data set.

Table S11. Hyperparameters resulting from the GPs generated in the experiment using centerpoints (1) as initial data set. The lower the hyperparameter for a variable, the higher its influence on the optimization objective.

Variable	STY	Conversion
$\theta_{R\_1/SM}$	4.5958	2.7890
θ_{Conc}	37.7925	132.9373
θ_{RT}	9.0351	9.0351
θ_{Temp}	0.0339	0.0233

Table S12. Results from the self-optimization experiment using center points (1) as initial data set. The adjusted variables were the ratio of reagent 1 (morpholine) to starting material (3,4-difluoronitrobenzene), concentration of starting material, residence time in the reactor and the temperature of the reactor. The objectives were space-time yield (STY) and conversion.

Entry	R_1 / SM	Conc. SM (mol/L)	RT (min)	Temp (°C)	STY (kg/L/h)	Conversion
Inital_1	0.69	0.300	4.25	105.0	0.07	9.0%
Inital_2	0.69	0.300	4.25	105.0	0.08	8.1%
Self_1	1.31	0.322	2.79	131.6	0.33	30.4%
Self_2	1.23	0.393	2.50	158.4	0.72	49.2%
Self_3	2.20	0.387	2.69	76.6	0.19	16.8%
Self_4	0.90	0.283	2.73	82.4	0.01	14.2%
Self_5	1.55	0.400	3.32	68.7	0.09	8.6%
Self_6	0.91	0.394	2.50	152.9	0.51	34.9%
Self_7	1.83	0.376	2.97	159.5	0.92	70.5%
Self_8	2.25	0.335	3.03	156.9	0.86	73.9%
Self_9	2.43	0.220	2.68	159.8	0.44	60.2%
Self_10	2.47	0.394	2.94	158.6	1.25	83.3%
Self_11	2.42	0.375	3.04	160.0	1.13	82.4%
Self_12	2.46	0.351	3.01	159.5	1.03	79.7%
Self_13	2.61	0.313	3.54	159.9	0.81	81.6%
Self_14	3.00	0.395	3.42	152.8	1.29	90.5%
Self_15	3.00	0.296	4.00	156.8	0.71	89.5%
Self_16	2.93	0.399	5.66	146.4	0.88	96.5%
Self_17	2.30	0.398	6.00	160.0	0.82	96.3%
Self_18	2.99	0.372	5.12	157.3	0.90	96.6%
Self_19	2.95	0.400	5.57	153.8	0.91	98.4%
Self_20	2.98	0.337	3.41	150.9	0.94	87.6%
Self_21	2.76	0.380	3.67	148.8	1.06	87.0%
Self_22	2.68	0.390	5.26	152.3	0.87	95.3%
Self_23	2.34	0.338	4.36	153.3	0.74	86.4%
Self_24	2.58	0.354	4.44	158.3	0.85	90.0%
Self_25	2.49	0.368	3.59	152.7	0.99	83.2%
Self_26	2.87	0.397	3.90	159.7	1.21	94.0%
Self_27	2.98	0.379	3.13	147.0	1.19	83.9%
Self_28	2.91	0.397	5.48	159.0	0.91	97.9%
Self_29	2.72	0.395	2.50	158.4	1.43	82.7%
Self_30	2.73	0.377	5.92	158.6	0.75	98.1%
Self_31	2.67	0.392	3.72	160.0	1.17	90.5%
Self_32	3.00	0.379	4.11	150.0	1.05	92.1%
Self_33	2.86	0.384	4.04	158.7	1.13	93.3%
Self_34	2.57	0.388	4.68	142.2	0.87	86.7%
Self_35	2.04	0.400	3.56	158.4	1.05	80.6%
Self_36	2.89	0.386	3.31	151.2	1.22	86.8%
Self_37	2.94	0.386	2.72	155.3	1.36	85.6%
Self_38	2.92	0.393	2.50	149.2	1.37	81.1%
Self_39	3.00	0.388	2.50	156.5	1.48	84.6%
Self_40	2.94	0.400	3.67	156.0	1.24	92.3%
Self_41	2.97	0.388	3.22	159.7	1.35	90.5%
Self_42	2.65	0.400	3.10	158.0	1.30	87.1%
Self_43	3.00	0.383	2.84	158.4	1.40	87.2%
Self_44	3.00	0.395	4.65	159.6	1.02	97.1%
Self_45	2.80	0.400	2.50	158.7	1.51	84.0%
Self_46	2.98	0.398	3.64	155.2	1.24	92.9%
Self_47	3.00	0.399	4.01	145.6	1.10	91.6%
Self_48	2.91	0.394	4.11	155.0	1.14	93.3%
Self_49	2.95	0.400	2.55	136.8	1.29	73.7%

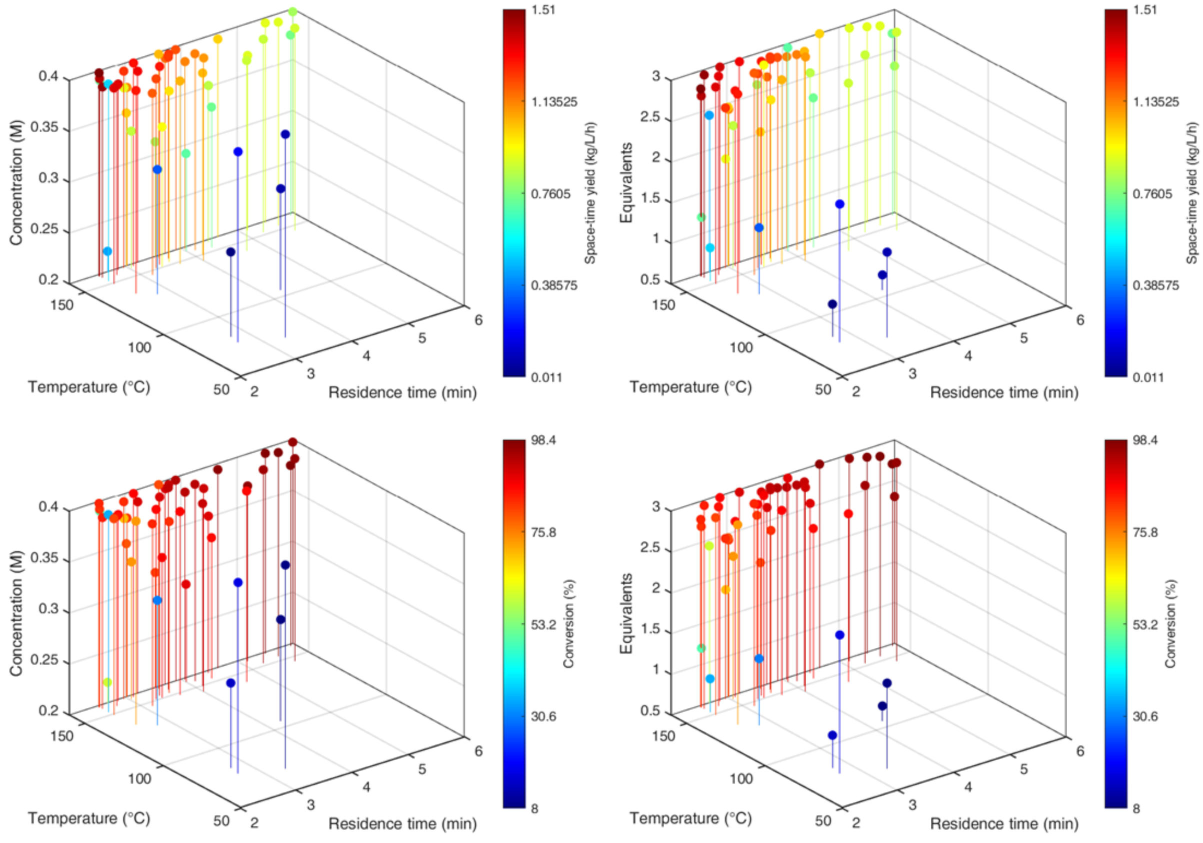

Figure S31. Optimization plots for the S_NAr reaction using center points (1) as initial data set.

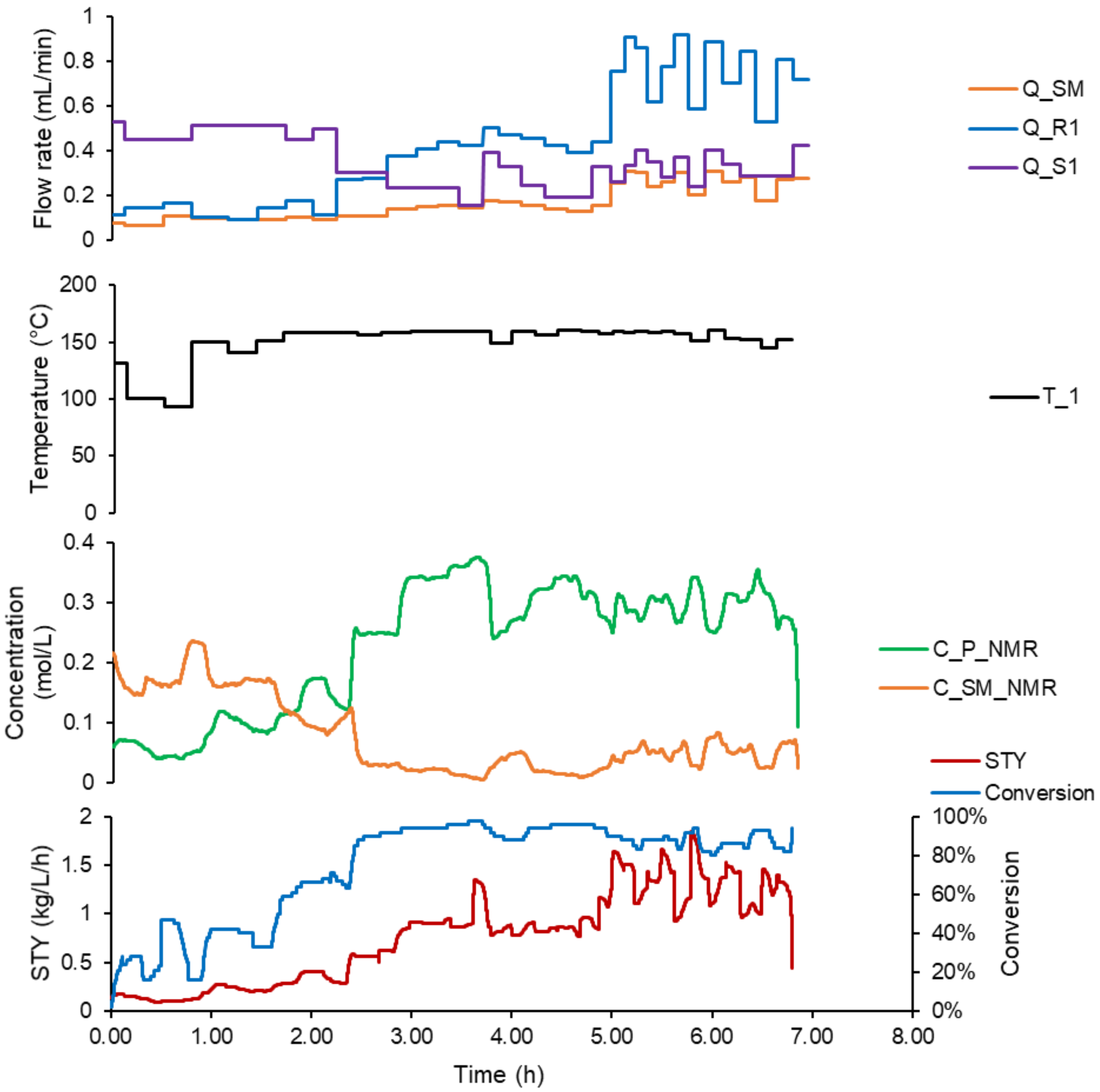

Figure S32. Real-time data from the S_NAr self-optimization experiment using center points (2) as initial data set.

Table S13. Hyperparameters resulting from the GPs generated in the experiment using centerpoints (2) as initial data set. The lower the hyperparameter for a variable, the higher its influence on the optimization objective.

Variable	STY	Conversion
$\theta_{R\_1/SM}$	4.8358	2.2666
θ_{Conc}	42.7773	158.1139
θ_{RT}	6.2120	9.0351
θ_{Temp}	0.0338	0.0080

Table S14. Results from the self-optimization experiment using center points (2) as initial data set. The adjusted variables were the ratio of reagent 1 (morpholine) to starting material (3,4-difluoronitrobenzene), concentration of starting material, residence time in the reactor and the temperature of the reactor. The objectives were space-time yield (STY) and conversion.

Entry	R_1 / SM	Conc. SM (mol/L)	RT (min)	Temp (°C)	STY (kg/L/h)	Conversion
Inital_1	0.69	0.300	4.25	105.0	0.07	9.0%
Inital_2	0.69	0.300	4.25	105.0	0.08	8.1%
Self_1	1.44	0.221	5.51	131.1	0.18	28.2%
Self_2	2.14	0.207	6.00	100.4	0.09	21.0%
Self_3	1.49	0.308	5.52	93.0	0.12	24.4%
Self_4	1.04	0.279	5.58	150.5	0.28	41.6%
Self_5	1.00	0.282	5.89	140.7	0.20	40.1%
Self_6	1.56	0.254	5.46	151.4	0.27	50.7%
Self_7	1.68	0.290	5.45	158.8	0.41	67.2%
Self_8	1.20	0.266	5.67	158.8	0.33	65.8%
Self_9	2.54	0.315	5.84	158.5	0.57	84.8%
Self_10	2.59	0.310	5.73	156.4	0.57	90.1%
Self_11	2.69	0.373	5.29	158.7	0.86	94.2%
Self_12	2.69	0.375	4.93	159.3	0.91	94.0%
Self_13	2.79	0.396	5.02	160.0	0.95	96.6%
Self_14	2.97	0.396	5.51	159.5	0.90	98.2%
Self_15	2.84	0.330	3.72	159.2	0.87	92.2%
Self_16	2.75	0.352	4.11	148.7	0.88	86.5%
Self_17	2.90	0.366	4.66	159.4	0.92	94.8%
Self_18	2.96	0.376	5.25	156.5	0.87	96.4%
Self_19	2.96	0.352	5.29	160.0	0.77	97.3%
Self_20	2.78	0.341	4.32	159.5	0.84	93.1%
Self_21	2.97	0.400	3.13	157.4	1.27	91.0%
Self_22	2.95	0.397	2.58	159.0	1.48	87.6%
Self_23	2.84	0.387	2.54	158.0	1.46	85.8%
Self_24	2.55	0.400	3.29	159.9	1.27	86.8%
Self_25	3.00	0.393	3.03	158.2	1.37	88.8%
Self_26	3.00	0.383	2.50	159.1	1.44	85.7%
Self_27	2.86	0.398	3.88	157.1	1.17	92.9%
Self_28	2.86	0.388	2.50	150.8	1.35	80.7%
Self_29	2.70	0.400	3.07	160.0	1.38	87.6%
Self_30	2.98	0.400	2.82	153.8	1.44	86.8%
Self_31	3	0.3697	4.1975	152.3	0.99	93.2%
Self_32	2.96	0.3934	2.8928	145.4	1.28	83.2%

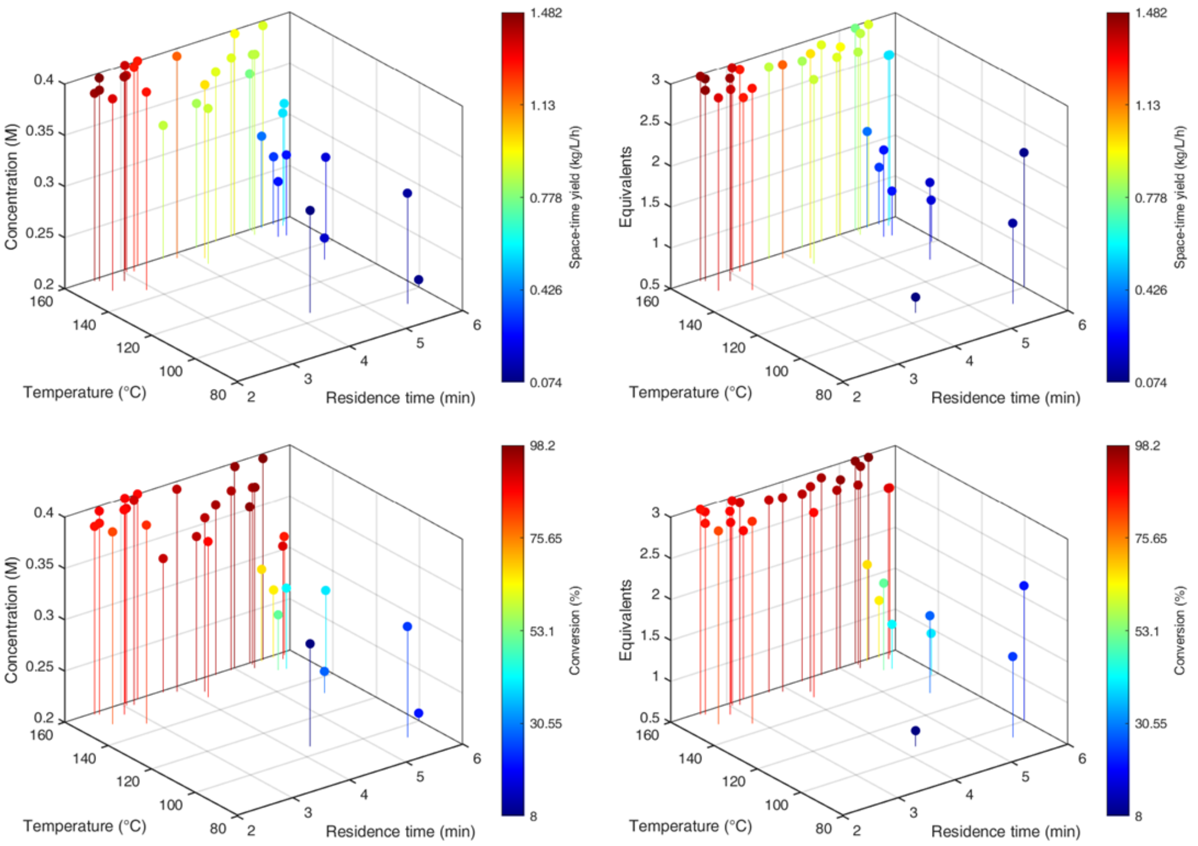

Figure S33. Optimization plots for the S_NAr reaction using center points (2) as initial data set.

5.4.6 Self-Optimization (3 Objective)

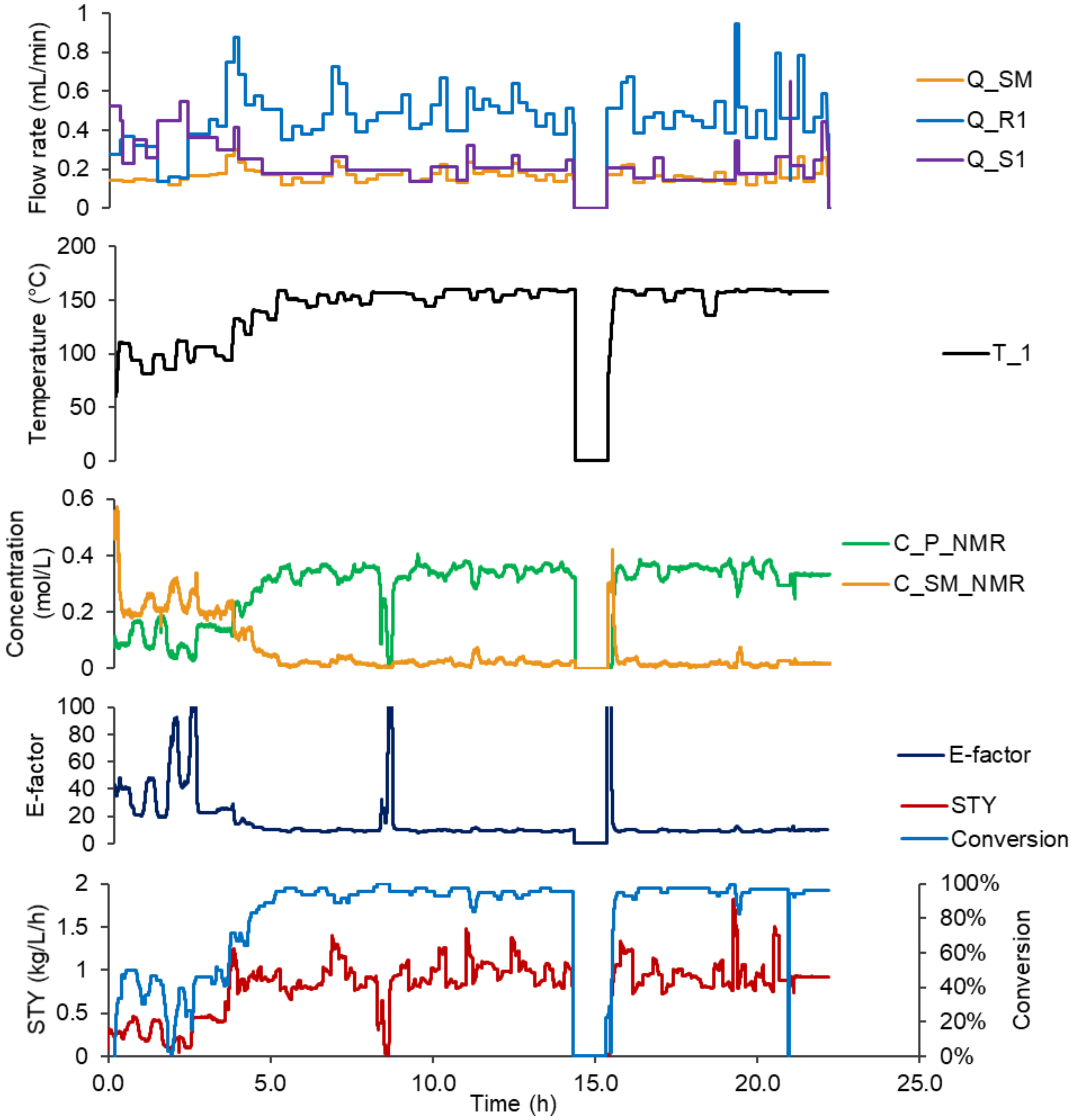

Figure S34. Real-time data from the S_NAr self-optimization experiment with 3 objectives, using center points as initial data set. The values at zero at around 15 h of processing time indicates that the experiments were continued on the next day.

Table S15. Hyperparameters resulting from the GPs generated in the experiment with 3 objectives, using center points as initial data set. The lower the hyperparameter for a variable, the higher its influence on the optimization objective.

Variable	STY	Conversion	E-Factor
$\theta_{R\_1/SM}$	3.3751	2.5907	3.2199
θ_{Conc}	40.7453	32.6134	30.6926
θ_{RT}	8.8315	9.0351	9.0351
θ_{Temp}	0.0758	0.0400	0.0547

Table S16. Results from the self-optimization experiment with 3 objectives, using center points as initial data set. The adjusted variables were the ratio of reagent 1 (morpholine) to starting material (3,4-difluoronitrobenzene), concentration of starting material, residence time in the reactor and the temperature of the reactor. The objectives were space-time yield (STY), conversion and E-factor.

Entry	R_1 / SM	Conc. SM (mol/L)	RT (min)	Temp (°C)	STY (kg/L/h)	Conversion	E-factor
Inital_1	1.95	0.300	4.25	110.0	0.27	33.1%	40.15
Inital_2	2.50	0.300	4.25	110.0	0.27	34.3%	40.27
Self_1	2.68	0.375	5.43	93.8	0.40	50.2%	20.68
Self_2	2.15	0.365	4.87	81.1	0.20	29.8%	47.02
Self_3	2.20	0.398	5.58	98.9	0.41	47.4%	19.78
Self_4	1.04	0.372	5.53	85.6	0.10	17.8%	86.82
Self_5	1.34	0.316	5.32	111.8	0.20	32.7%	42.51
Self_6	1.03	0.350	4.76	92.6	0.09	22.2%	106.70
Self_7	2.25	0.371	4.42	106.5	0.45	47.1%	22.53
Self_8	2.69	0.360	4.26	98.3	0.43	47.8%	24.93
Self_9	2.42	0.390	4.46	94.3	0.41	40.6%	24.76
Self_10	2.75	0.400	2.95	132.4	1.06	72.4%	14.33
Self_11	2.93	0.376	2.52	130.3	1.01	66.7%	17.77
Self_12	2.94	0.399	3.43	118.0	0.88	65.0%	14.77
Self_13	2.69	0.388	3.96	140.1	0.94	83.9%	11.77
Self_14	3.00	0.393	4.11	139.2	1.04	87.6%	10.21
Self_15	2.97	0.400	4.71	132.2	0.88	88.4%	10.46
Self_16	3.00	0.398	4.70	158.7	1.01	96.8%	8.99
Self_17	2.98	0.354	6.00	151.1	0.66	97.4%	10.86
Self_18	2.75	0.395	5.17	149.7	0.87	94.4%	9.67
Self_19	2.90	0.381	5.78	143.9	0.79	94.5%	9.37
Self_20	2.99	0.388	5.81	155.0	0.83	97.6%	8.71
Self_21	2.92	0.390	4.73	147.7	0.95	93.6%	9.61
Self_22	2.99	0.393	3.25	156.9	1.28	90.2%	10.49
Self_23	2.97	0.396	3.69	150.3	1.15	90.1%	10.28
Self_24	2.88	0.398	4.72	154.1	0.97	95.2%	9.36
Self_25	2.93	0.400	6.00	146.1	0.80	96.4%	8.94
Self_26	2.97	0.391	5.22	157.3	0.87	98.9%	9.44
Self_27	2.89	0.384	4.55	157.1	0.95	94.5%	9.99
Self_28	2.98	0.397	4.09	155.8	1.11	93.8%	9.46
Self_29	3.00	0.398	5.85	150.9	0.81	97.6%	8.90
Self_30	2.96	0.396	5.41	144.6	0.85	94.6%	9.44
Self_31	3.00	0.385	4.37	152.8	1.00	93.6%	9.72
Self_32	2.99	0.400	3.57	160.0	1.26	93.1%	9.69
Self_33	2.78	0.394	5.50	159.8	0.86	96.4%	8.95
Self_34	2.98	0.397	6.00	159.9	0.83	98.2%	8.52
Self_35	2.63	0.400	3.42	151.1	1.13	84.5%	11.37
Self_36	2.84	0.399	4.50	158.1	1.03	95.0%	9.22
Self_37	2.98	0.397	4.22	160.0	1.09	95.4%	9.32
Self_38	2.82	0.399	4.28	149.1	1.02	91.2%	9.96
Self_39	2.99	0.400	4.91	154.9	0.98	95.8%	8.87
Self_40	2.81	0.400	3.53	159.7	1.21	91.3%	10.20
Self_41	2.99	0.396	4.37	159.0	1.09	95.2%	9.15
Self_42	2.98	0.400	4.75	152.4	0.97	95.4%	9.40
Self_43	2.91	0.399	5.55	160.0	0.88	97.4%	8.62
Self_44	2.83	0.393	4.59	159.5	1.01	94.5%	9.27
Self_45	2.73	0.395	5.72	158.2	0.81	96.5%	9.19
Self_46	2.97	0.371	4.30	160.0	1.00	93.5%	10.08
Self_47	2.93	0.391	4.49	160.0	1.05	95.1%	8.98
Self_48	2.96	0.391	3.60	159.0	1.20	92.1%	10.10
Self_49	2.98	0.393	3.48	158.6	1.24	91.7%	10.14
Self_50	2.93	0.392	6.00	155.3	0.83	97.1%	8.67
Self_51	2.99	0.400	5.11	159.7	0.96	97.5%	8.61
Self_52	3.00	0.359	4.40	159.7	0.94	94.6%	10.51
Self_53	2.99	0.398	5.79	149.3	0.82	97.2%	8.99
Self_54	3.00	0.392	4.78	157.5	1.01	96.6%	8.98
Self_55	3.00	0.399	5.06	160.0	0.95	98.3%	8.76
Self_56	3.00	0.394	5.29	159.3	0.90	98.0%	9.00

Self_57	3.00	0.400	5.83	136.1	0.80	95.0%	9.12
Self_58	2.99	0.400	4.37	156.7	1.11	95.6%	8.80
Self_59	3.00	0.379	5.95	158.0	0.76	98.9%	9.54
Self_60	3.00	0.393	2.50	160.0	1.47	86.6%	12.10
Self_61	2.98	0.400	4.61	157.7	1.06	96.3%	8.73
Self_62	3.00	0.364	6.00	160.0	0.76	98.3%	9.55
Self_63	2.95	0.400	4.70	159.9	1.06	96.6%	8.62
Self_64	2.75	0.389	5.99	159.5	0.75	98.4%	9.51
Self_65	3.00	0.400	3.02	160.0	1.40	96.9%	10.36
Self_66	3.00	0.369	4.79	158.0	0.93	95.6%	9.66

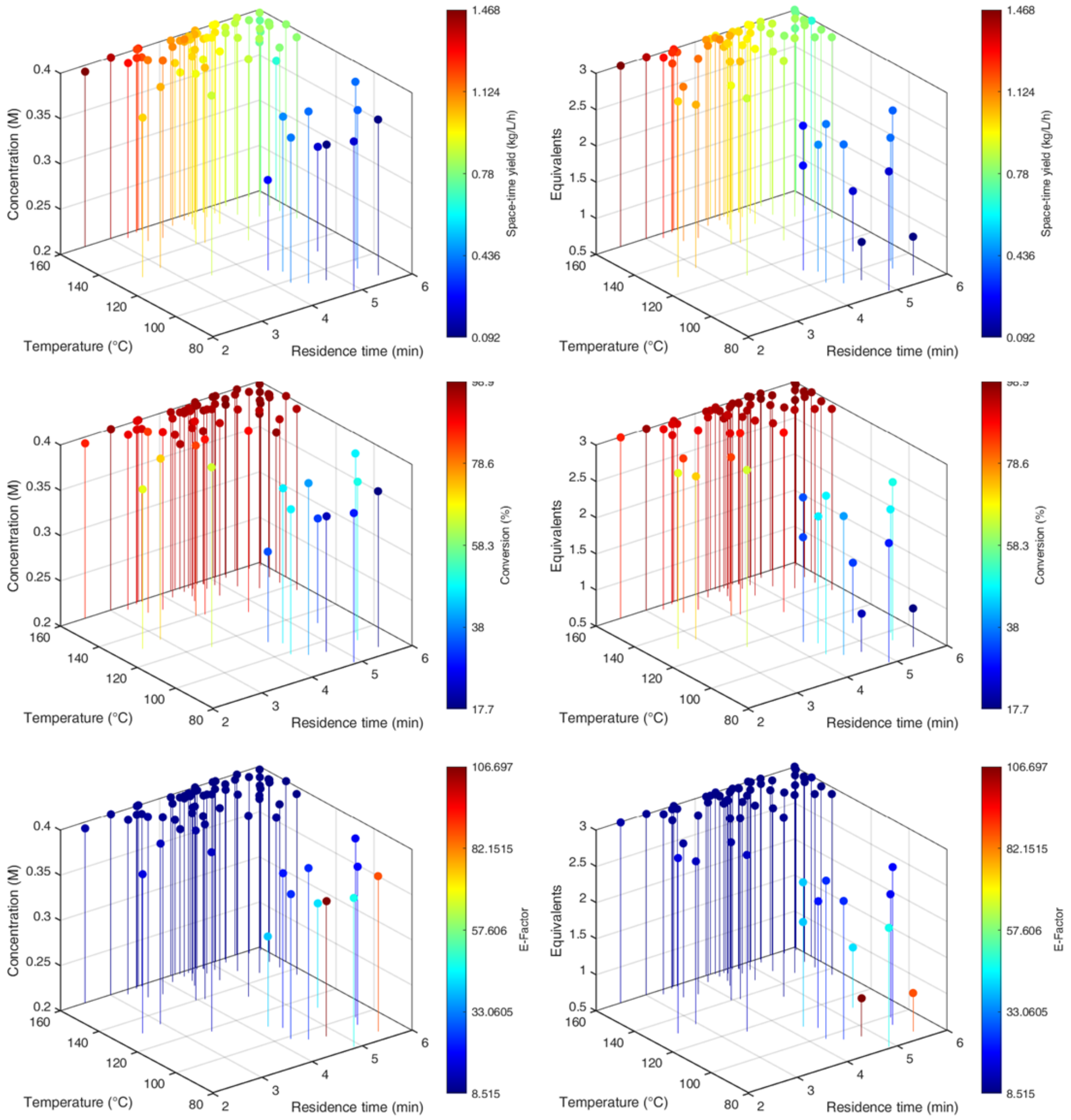

Figure S35. Optimization plots for the 3-objective optimization of the SNAr reaction using center points as initial data set.

6 Edaravone

6.1 Reactor Platform

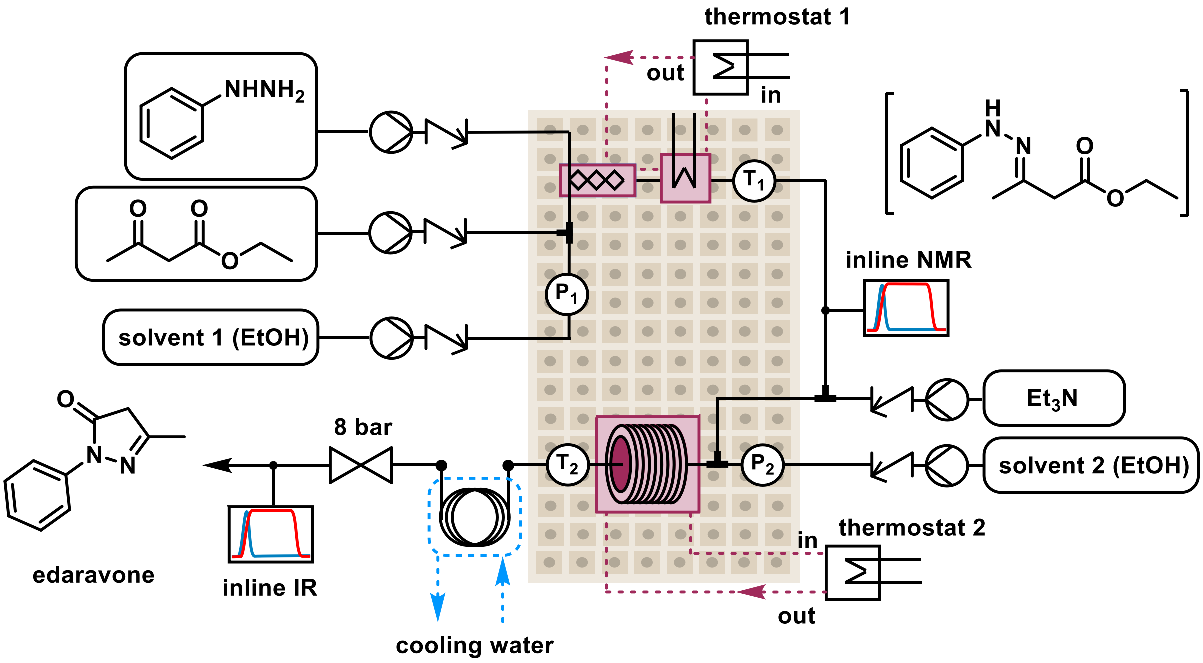

Figure S36. Detailed flow setup for the multi-step self-optimization of edaravone.

Preparation of stock solutions:

Phyenlhydrazine: The phenylhydrazine was pumped neat from a 250 mL Duran bottle with a septum.

Ethyl acetoacetate: The ethyl acetoacetate was pumped neat from a 250 mL Duran bottle with a septum.

Triethylamine: The triethylamine was pumped neat from a 250 mL Duran bottle with a septum.

Ethanol was pumped from 1.0 L Duran bottles.

The synthesis of edaravone was performed in a Modular MictroReaction System (Ehrfeld Mikrotechnik, MMRS). The phenylhydrazine, ethyl acetoacetate and solvent 1 (EtOH) streams were delivered with three SyrDos2 pumps (90 bar valve, 1.0 mL syringes) and entered the MMRS system through a 1/16" in/out connector (0711-2-0124-F, Hastelloy C-276). The solvent 1 stream passed through a pressure sensor module (0518-1-60x4-F, Hastelloy C-276) and was mixed with the ethyl acetoacetate stream in a T-type connecting module (0723-1-0004, Hastelloy C-276). The diluted ethyl acetoacetate was combined with the phenylhydrazine stream in a temperature-controlled cascade mixer (0216-3-0014-F, mixing structure 10 µL, Hastelloy C-276), followed by a temperature controlled coax heat

exchanger (0309-4-0004-F, 1.01 mL, Hastelloy C-276) used as a residence time module. The temperature in the cascade mixer and the heat exchanger was controlled by thermostat 1 (Huber, Ministat 240). Additionally, the reaction stream exited the MMRS system through a temperature sensor (0501-2-1004-X, Hastelloy C-276) and a 1/16" in/out connector (0711-2-0124-F, Hastelloy C-276). The outlet tubing from the MMRS system (0.9 mL, 0.8 mm i.d.) was connected to a 6-port valve prior to measuring the reaction stream with the benchtop NMR (Magritek, Spinsolve 43 Ulta). This 6-port valve allowed the NMR to be by-passed in case of re-shimming the instrument. NMR measurements at elevated pressure were enabled by placing a PTFE tube (3.2 mm o.d., 1.6 mm i.d., length = 600 mm, internal volume = 1.14 mL) through the NMR (see section 4.1.4). The reaction stream left the NMR through PFA tubing (0.9 mL, 0.8 mm i.d.) and entered again the MMRS for the second step through a 1/16" in/out connector (0711-2-0124-F, Hastelloy C-276).

The triethylamine stream was delivered with a SyrDos2 pump (30 bar valve, 2.5 mL syringes). The solvent 2 (EtOH) stream was pumped with a HPLC pump (Knauer AZURA P 4.1S, 10 mL/min pump head made of Hastelloy) and was connected to a cartridge BPR (IDEX, green 34 bar) to ensure enough back pressure for the HPLC pump. The two streams (triethlyamine and solvent 2) were mixed prior to the MMRS system in a T-piece (PTFE, 1.5 mm i.d.) and entered the MMRS system through a 1/16" in/out connector (0711-2-0124-F, Hastelloy C-276), followed by a pressure sensor module (0518-1-60x4-F, Hastelloy C-276). The reaction stream from the first reaction was combined with the diluted triethylamine stream in a T-type connecting module (0723-1-0004, Hastelloy C-276). The reaction mixture passed through a capillary reactor (0214-1-1004-F, built in connection body of 2.04 mL, Hastelloy C-276) which was temperature controlled by thermostat 2 (Huber, Ministat 240). After the capillary reactor the reaction solution passed through another temperature sensor (0501-2-1004-X, Hastelloy C-276) and exited the MMRS system via a 1/16" in/out connector (0711-2-0124-F, Hastelloy C-276). The reaction mixture was cooled after the MMRS to room temperature in an additional PFA tube (0.6 mL, 0.8 mm i.d.) which was placed in an actively cooled water bath. A membrane based BPR (Zaiput, BPR-10) was used to maintain 10 bar for both reaction steps. The outlet stream of the BPR was connected to the flow cell (Mettler Toledo, Micro Flow Cell DS SiComp) of the FTIR (Mettler Toledo, ReactIR 15) and analyzed prior to collection in a receiver vessel.

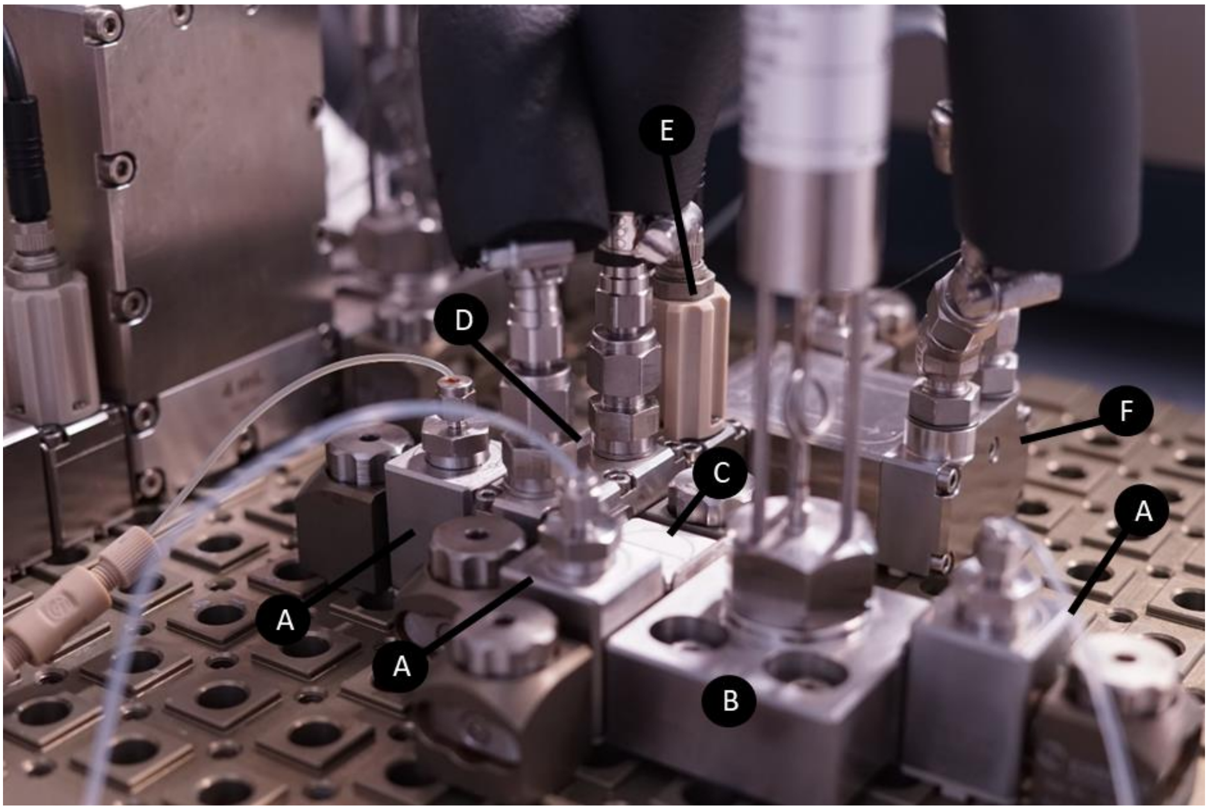

Figure S37. Image of the first edaravone reaction step in the MMRS system. (A) in/out connector, (B) pressure sensor module, (C) T-type connecting module, (D) cascade mixer, (E) temperature sensor module, (F) coax heat exchanger.

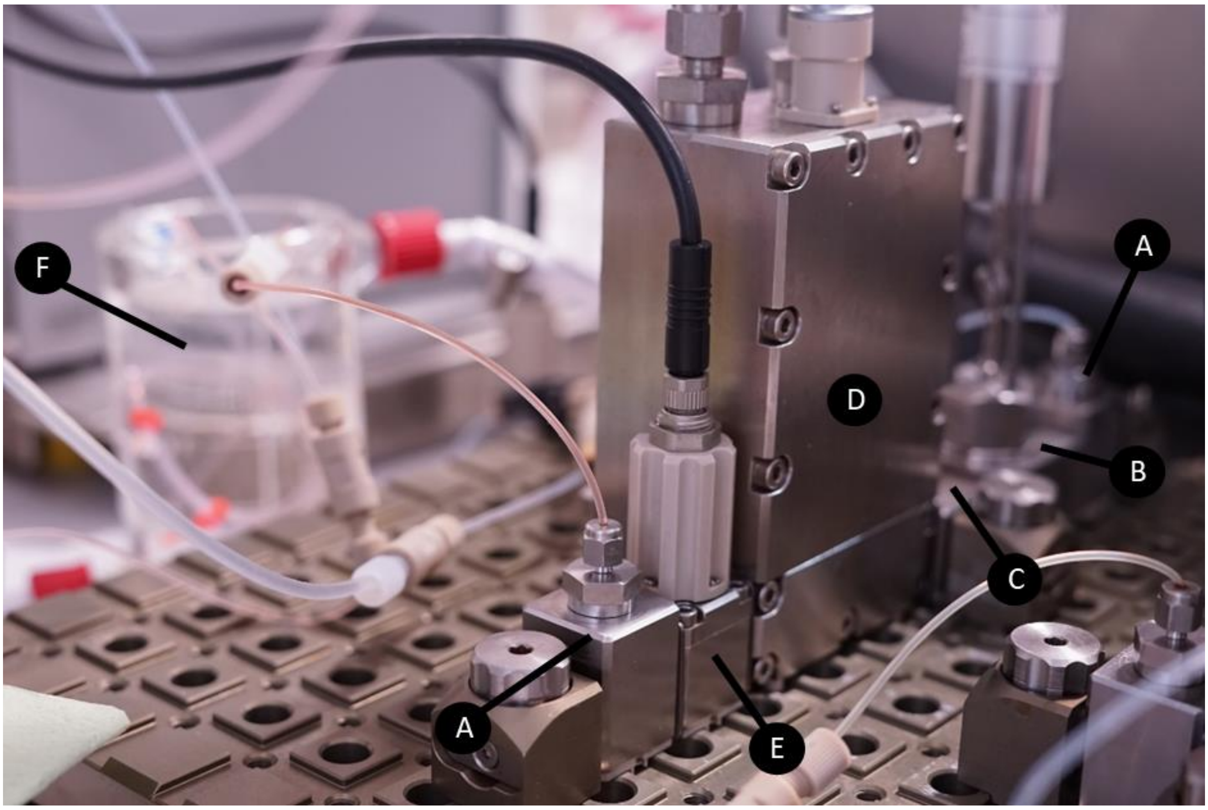

Figure S38. Image of the second edaravone reaction step in the MMRS system. (A) in/out connector, (B) pressure sensor module, (C) T-type connecting module, (D) capillary reactor, (E) temperature sensor module, (F) actively cooled water bath.

6.2 Reactor Inputs and Reactor Outputs

During the self-optimization experiment there are reactor inputs which are fixed and manipulated. Additionally there are reactor inputs which are depending on the manipulated inputs (**Figure S39**). Reactor outputs could be either measured directly from the NMR or calculated from the measured outputs.

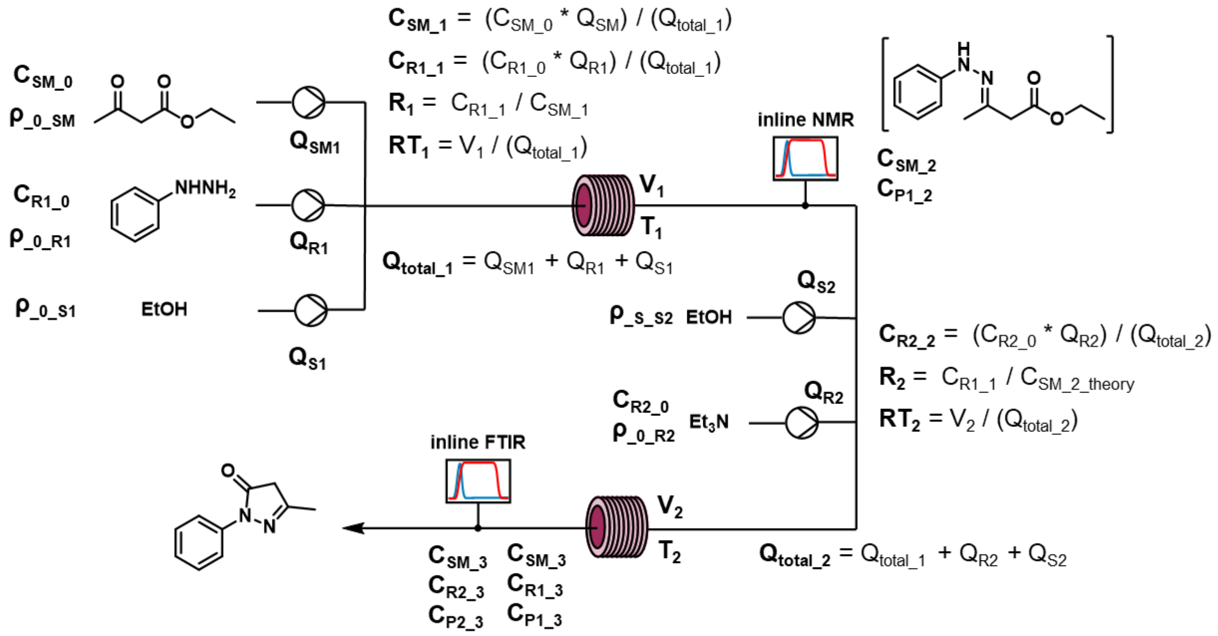

Figure S39. Schematic overview of reactor inputs and reactor outputs for the edaravone self-optimization experiments.

The following variables (reactor inputs) were fixed during a self-optimization experiment:

C_{SM_0}: initial concentration of starting material

C_{R1_0}: initial concentration of reagent 1

C_{R2_0}: initial concentration of reagent 2

V_1: reactor volume for the reaction step 1

V_2: reactor volume for the reaction step 2

$\rho_{\_0\_SM}$: density of starting material solution

$\rho_{\_0\_R1}$: density of reagent 1 solution

$\rho_{\_0\_R2}$: density of reagent 2 solution

$\rho_{\_0\_S1}$: density of solvent 1 solution

$\rho_{\_0\_S2}$: density of solvent 2 solution

The following variables (reactor inputs) were manipulated during a self-optimization experiment:

Q_{SM1}: volume flow starting material

Q_{R1}: volume flow reagent 1

Q_{R2}: volume flow reagent 2

Q_{S1}: volume flow solvent 1

Q_{S2}: volume flow solvent 2

T_1: temperature of the reactor

T_2: temperature of the reactor

The following variables (reactor inputs) were depending on the manipulated inputs during a self-optimization experiment:

C_{SM_1}: concentration of starting material

C_{R1_1}: concentration of reagent 1

R_1: ratio of reagent 1 to starting material

RT_1: residence time of step 1 (time of material in the reactor "reaction time")

C_{R2_2}: concentration of reagent 2

R_2: ratio of reagent 2 to starting material

RT_2: residence time of step 2 (time of material in the reactor "reaction time")

The following variables (reactor outputs) were measured during a self-optimization experiment.

C_{SM_2}: concentration of starting material (NMR)

C_{P1_2}: concentration of product 1 (NMR)

Q_{total_1}: total volume flow for first step

C_{SM_3}: concentration of starting material (FTIR)

C_{R1_3}: concentration of reagent 1 (FTIR)

C_{R2_3}: concentration of reagent 2 (FTIR)

C_{P1_3}: concentration of product 1 (FTIR)

C_{P2_3}: concentration of product 2 (FTIR)

Q_{total_2}: total volume flow for second step

The following variables (reactor outputs) were calculated for the measured outputs during a self-optimization experiment.

Throughput: TP_1 = C_{P1_2} * Q_{total_1}

Conversion step 1: Conv_1 = 1 - (C_{SM_2} / ((C_{SM_0} * Q_{SM_1}) / Q_{total_1}))

Yield step 1: Yield_1 = C_{P1_2} / ((C_{SM_0} * Q_{SM_1}) / Q_{total_1})

Selectivity step 1: Selectivity_1 = Yield_1 / Conv_1

Space-time yield step 1: STY_1 = (C_{P1_2} * Q_{total_1} / V1 * M_{P1} / 1000 * 60)

E-Factor step 1: E_Factor_1 = ((($\rho_{\_0\_SM}$ * Q_{SM}) + ($\rho_{\_0\_R1}$ * Q_{R1}) + ($\rho_{\_0\_S1}$ * Q_{S1})) / (C_{P1_2} * M_{P1} * Q_{total_1} / 1000)) – 1

Throughput: TP_2 = C_{P2_3} * Q_{total_2}

Conversion step 2: Conv_2 = 1 - (C_{P1_3} / $C_{P1\_2\_dil}$)

Yield step 2: Yield_2 = C_{P2_3} / ((C_{SM_0} * Q_{SM_1}) / Q_{total_2})

Selectivity step 2: Selectivity_2 = Yield_2 / Conv_2

Space-time yield step 2: STY_2 = (C_{P2_3} * Q_{total_2} / V_2 * M_{P2} / 1000 * 60)

E-Factor step 2: E_Factor_2 = ((($\rho_{\_0\_SM}$ * Q_{SM_1}) + ($\rho_{\_0\_R1}$ * Q_{R1}) + ($\rho_{\_0\_S1}$ * Q_{S1}) + ($\rho_{\_0\_R2}$ * Q_{R2}) + ($\rho_{\_0\_S2}$ * Q_{S2})) / (C_{P2_3} * M_{P2} * Q_{total_2} / 1000)) - 1

6.3 Optimization Parameters

In the self-optimization experiments of the edaravone synthesis the optimization algorithm could adjust in total seven different variables: ratio of reagent 1 (phenylhydrazine) to starting material (ethyl acetoacetate), concentration of starting material, residence time in the reactor (RT), the temperature of the reactor (Temp 1), the temperature of the second reactor (Temp 2), ratio of reagent 2 (triethylamine) to starting material and a dilution factor which was based on the total flow rate. The adjustable variables had the following upper and lower bounds (**Table S17**). The objective of the optimization was to simultaneously maximize the yield in the first reaction step, maximize the STY in the second reaction step and minimize the overall equivalents of reagents in the multi-step process, as defined by Equation (3).

Table S17. Lower and upper bounds for the seven adjustable variables in the self-optimization experiments for edaravone.

Limits	R_1 / SM	Conc. SM (mol/L)	RT (min)	Temp 1 (°C)	Temp 2 (°C)	R_2 / SM	Dilution
Lower	0.5	1.0	0.5	10	25	0.5	0
Upper	1.5	2.4	1.5	50	130	3.0	1

$$\text{minimize } [-\ln\left(\text{Yield}_{\text{Step 1}}\right), -\ln\left(\text{STY}_{\text{Step 2}}\right), \ln(\text{equivalents}_{\text{overall}}) \qquad (3)$$

6.4 Reaction Optimization (Edaravone Synthesis)

6.4.1 Batch Experiments

Ethyl (E)-3-(2-phenylhydrazineylidene)butanoate (6)

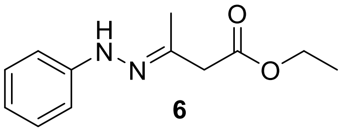

6

Ethyl acetoacetate (6.01 g, 46.2 mmol) was dissolved in DCM (25 mL) and cooled to 0 °C. Phenyl hydrazine (4.99 g, 46.2 mmol, 1.0 equiv) was added slowly at 0 °C under stirring. The resulting solution was stirred for 5 min at 0 °C, subsequently dried using MgSO4 and filtered through a glass frit (Por. 4). Solvent evaporation under reduced pressure and freeze-drying afforded the desired product as a light-yellow solid (8.06 g, 80 %), which was used without further purification.

^{1}H NMR (300 MHz, DMSO-d_6, δ): 8.85 (s, 1H, NH), 7.28 – 6.92 (m, 4H, ArH), 6.77 – 6.59 (m, 1H, ArH), 4.10 (q, *J* = 7.1 Hz, 2H, CH_2), 3.30 (s, 2H, CH_2), 1.91 (s, 3H, CH_3), 1.20 (t, *J* = 7.1 Hz, 3H, CH_3); ^{13}C NMR (75 MHz, DMSO-d_6, δ): 170.3, 146.2, 139.7, 128.8, 118.4, 112.4, 60.3, 44.0, 15.8, 14.1; HRMS (ESI, positive mode) calculated for $C_{12}H_{16}O_2N_2$ $(M+H)^+$: 221.1285, found: 221.1283

Edaravone (7)

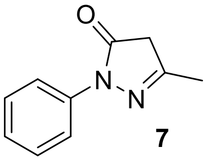

7

Edaravone (**7**) was synthesized using a modified literature procedure.[2] A round-bottom flask (25 mL) was charged with ethyl acetoacetate (4.8 mL, 37.9 mmol). Phenyl hydrazine (4.15 mL, 42.2 mmol, 1.1 equiv) was added slowly with stirring at room temperature. The resulting yellow reaction mixture was then heated to reflux (110 °C) for 2 h (reaction monitoring via HPLC). After reaction completion, it was cooled down to r.t., which led to complete solidification of the reaction mixture. The yellow product was washed with Et_2O (3 × 15 mL) to afford the desired product as an off-white solid (5.28 g, 80 %), which was used without further purification.

^{1}H NMR (300 MHz, $CDCl_3$, δ): 8.02 – 7.64 (m, 2H), 7.39 (dd, *J* = 8.5, 7.5 Hz, 2H), 7.17 (t, *J* = 7.4 Hz, 1H), 3.42 (s, 2H), 2.19 (s, 3H); ^{13}C NMR (75 MHz, $CDCl_3$, δ): 170.7, 156.4, 138.2,

128.9, 125.2, 119.0, 43.2, 17.1; HRMS (ESI, positive mode) calculated for $C_{10}H_{10}ON_2$ $(M+H)^+$: 175.0866, found: 175.0864

6.4.2 Self-Optimization

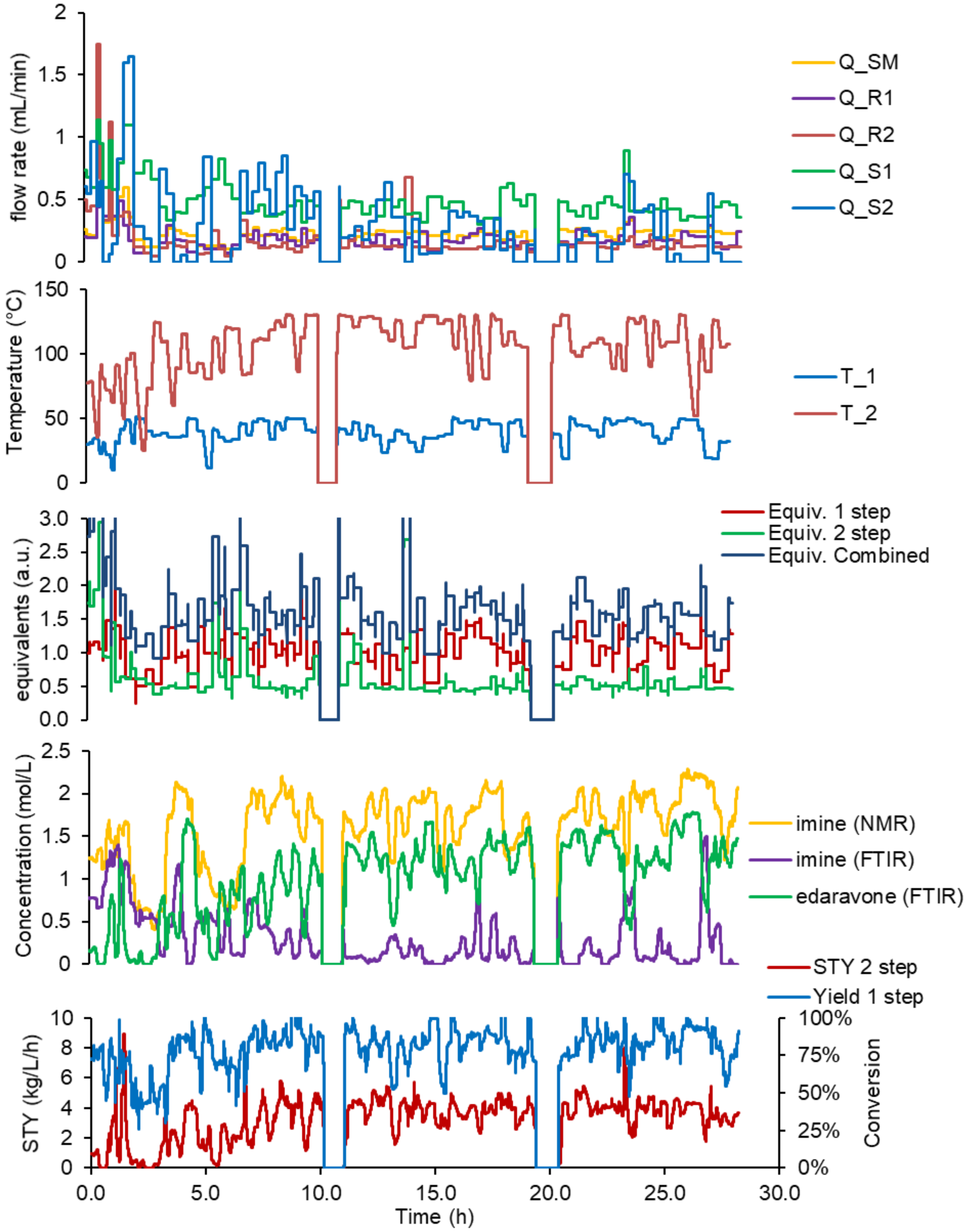

Figure S40. Real-time data from the edaravone self-optimization experiment using center points as initial data set. The values at zero at around 10 h and 20 h of processing time indicate that the experiments were continued on the next day.

Table S18. Hyperparameters resulting from the GPs generated in the multi-step self-optimization using center points as initial data set. The lower the hyperparameter for a variable, the higher its influence on the optimization objective.

Variable	Yield Step 1	STY Step 2	Overall Equiv
$\theta_{R\_1/SM}$	2.0429	13.9007	2.7639
θ_{Conc}	3.6636	9.9053	22.5877
θ_{RT}	3.3752	31.6228	17.5816
θ_{Temp_1}	0.3689	0.7906	0.7906
θ_{Temp_2}	0.3012	0.0090	0.3012
$\theta_{R\_2/SM}$	12.6491	12.6491	0.4476
$\theta_{Dilution}$	31.6228	22.1120	31.6228

Table S19. Results from the multi-step self-optimization experiment using center points as initial data set. The adjusted variables were the ratio of reagent 1 (phenylhydrazine) to starting material (ethyl acetoacetate), concentration of starting material, residence time in the reactor (RT), the temperature of the reactor (Temp 1), the temperature of the second reactor (Temp 2), ratio of reagent 2 (triethylamine) to starting material and a dilution factor which was based on the total flow rate. The objectives were yield in the first step, space-time yield (STY) in the second step and the overall equivalents of reagents in the multi-step process.

Entry	R_1 / SM	Conc. SM (mol/L)	RT (min)	Temp 1 (°C)	Temp 2 (°C)	R_2 / SM	Dilution	Yield Step 1	STY Step 2	Overall equiv.
Inital_1	1.00	1.70	1.00	30.0	77.5	1.75	0.50	72.7%	0.89	2.7
Inital_2	1.00	1.70	1.00	30.0	77.5	1.75	0.50	75.4%	1.04	2.8
Self_1	1.10	1.60	1.10	31.0	78.5	1.75	0.50	82.3%	0.00	3.1
Self_2	1.15	1.69	1.18	34.6	35.7	1.91	0.95	65.1%	2.32	2.0
Self_3	1.04	2.40	0.57	30.8	80.4	0.96	0.30	73.2%	3.11	2.4
Self_4	1.50	1.95	0.92	22.0	71.9	0.97	0.02	57.5%	0.98	2.8
Self_5	1.35	2.31	0.50	10.0	62.5	1.46	0.03	76.1%	4.89	2.0
Self_6	1.38	2.09	0.93	30.9	90.8	0.58	0.11	72.9%	0.74	1.9
Self_7	1.21	2.29	0.67	41.4	49.5	0.66	0.45	40.7%	0.43	1.2
Self_8	0.61	2.40	0.60	48.6	95.5	0.60	0.80	43.9%	0.00	1.1
Self_9	0.51	1.48	1.24	49.9	25.0	0.70	0.29	43.7%	0.22	1.3
Self_10	0.71	1.00	1.19	40.2	73.1	0.58	0.18	54.9%	1.80	0.9
Self_11	0.55	1.17	1.44	36.6	123.9	0.50	0.00	84.3%	2.51	1.4
Self_12	0.92	2.07	1.50	38.3	109.5	0.53	0.92	87.6%	1.31	1.9
Self_13	1.39	2.40	1.34	35.4	60.3	0.50	0.61	85.0%	3.95	1.4
Self_14	0.94	2.39	1.41	35.6	88.5	0.52	0.03	79.0%	4.12	1.4
Self_15	0.97	2.33	1.49	36.5	115.1	0.50	0.07	52.1%	1.37	1.2
Self_16	0.50	2.06	1.49	49.8	85.6	0.74	0.04	88.1%	1.93	1.8
Self_17	1.38	1.30	1.50	40.2	92.6	0.52	0.39	65.2%	0.26	1.5
Self_18	1.01	1.22	1.44	11.8	80.6	0.57	1.00	70.9%	2.82	2.7
Self_19	1.02	1.18	1.32	41.1	112.3	1.69	0.03	62.7%	1.52	1.8
Self_20	1.22	1.00	1.08	35.4	86.0	0.64	0.02	64.5%	1.81	1.4
Self_21	0.91	1.06	1.50	32.5	120.1	0.54	0.00	84.0%	2.95	1.9
Self_22	1.30	1.45	1.50	33.5	119.2	0.69	0.12	88.1%	1.91	2.6
Self_23	1.20	2.13	1.45	45.7	83.8	1.40	0.87	91.9%	2.72	1.7
Self_24	1.09	2.18	1.49	37.3	84.9	0.71	0.48	86.3%	4.59	1.6
Self_25	1.11	2.40	1.27	47.1	109.7	0.50	0.59	81.6%	2.15	1.3
Self_26	0.80	2.10	1.49	49.8	111.7	0.51	0.98	90.2%	3.55	1.8
Self_27	1.34	2.11	1.48	47.9	111.0	0.54	0.71	84.6%	4.63	1.6
Self_28	1.14	2.37	1.41	33.9	118.5	0.50	0.44	89.4%	4.16	1.4
Self_29	0.96	2.39	1.47	46.6	125.3	0.50	0.88	81.4%	4.22	1.6

Self_30	1.05	2.40	1.34	41.5	121.3	0.50	0.94	94.2%	4.00	1.6
Self_31	1.15	1.88	1.45	50.0	130.0	0.52	0.31	85.0%	1.27	1.4
Self_32	0.99	1.72	1.50	50.0	86.5	0.50	0.75	89.3%	3.52	2.0
Self_33	1.50	2.23	1.43	50.0	112.2	0.50	0.63	77.8%	4.29	1.4
Self_34	0.79	2.25	1.33	41.2	129.5	0.65	0.38	88.1%	4.42	2.1
Self_35	1.17	1.92	1.44	39.1	130.0	1.00	0.67	90.6%	4.04	1.8
Self_36	1.27	1.87	1.48	41.5	129.6	0.60	0.24	89.0%	4.25	2.1
Self_37	1.28	1.71	1.40	38.1	128.8	0.91	0.25	80.9%	3.90	2.0
Self_38	0.85	1.94	1.47	46.7	121.0	1.17	0.35	86.3%	4.32	1.5
Self_39	1.03	2.30	1.50	33.5	127.8	0.53	0.22	81.7%	4.16	1.4
Self_40	0.87	2.15	1.44	44.0	128.7	0.56	0.38	87.4%	4.92	1.6
Self_41	1.11	2.40	1.42	36.8	127.2	0.50	0.58	53.2%	1.96	1.0
Self_42	0.55	2.25	1.42	23.5	117.4	0.50	0.75	82.9%	4.20	1.5
Self_43	0.95	2.22	1.38	32.6	110.3	0.54	0.41	70.8%	3.77	1.2
Self_44	0.80	2.11	1.35	40.5	125.6	0.52	0.40	86.0%	4.68	3.7
Self_45	1.02	2.32	1.48	37.9	104.6	2.65	0.05	79.6%	3.92	1.3
Self_46	0.89	2.40	1.50	31.3	105.0	0.50	0.42	85.2%	4.18	1.8
Self_47	1.36	2.28	1.50	24.2	124.0	0.52	0.08	55.6%	2.79	1.0
Self_48	0.55	2.02	1.47	34.8	129.3	0.51	0.09	84.4%	4.35	1.4
Self_49	0.94	2.22	1.42	36.1	123.5	0.50	0.29	89.0%	3.63	1.9
Self_50	1.31	1.57	1.48	37.6	126.2	0.62	0.50	82.2%	3.36	1.3
Self_51	0.94	1.99	1.42	50.0	104.3	0.51	0.42	89.5%	4.14	1.7
Self_52	1.23	2.00	1.50	46.3	129.2	0.50	0.49	89.4%	2.44	1.8
Self_53	1.40	2.07	1.46	48.0	79.5	0.50	0.44	88.2%	4.43	1.9
Self_54	1.42	2.36	1.47	42.1	129.3	0.51	0.40	88.7%	3.61	1.7
Self_55	1.22	2.28	1.47	49.2	80.9	0.50	0.12	86.4%	4.92	1.8
Self_56	1.13	2.40	1.49	45.9	130.0	0.69	0.43	82.2%	4.02	1.6
Self_57	1.03	1.58	1.28	45.5	126.3	0.50	0.10	76.9%	4.00	1.5
Self_58	0.98	1.79	1.15	24.4	106.6	0.52	0.23	79.5%	3.38	1.3
Self_59	0.88	1.88	1.43	46.3	116.8	0.54	0.00	79.1%	4.07	1.4
Self_60	0.88	2.24	1.39	49.1	107.0	0.61	0.02	70.7%	2.66	1.2
Self_61	0.78	1.69	1.47	36.3	105.2	0.50	0.16	76.5%	4.31	1.3
Self_62	0.83	2.26	1.32	37.6	121.6	0.55	0.15	75.8%	3.71	1.2
Self_63	0.77	2.40	1.50	18.5	130.0	0.50	0.01	86.8%	5.01	1.7
Self_64	1.25	2.37	1.33	50.0	101.8	0.52	0.19	88.7%	4.07	2.1
Self_65	1.47	1.87	1.32	44.7	98.4	0.64	0.29	89.3%	4.40	1.8
Self_66	1.22	2.27	1.50	43.9	105.1	0.62	0.27	78.6%	3.98	1.3
Self_67	0.81	2.18	1.39	41.1	108.4	0.50	0.00	84.8%	4.05	1.6
Self_68	1.09	1.80	1.33	36.0	111.9	0.57	0.00	89.0%	3.69	1.6
Self_69	1.09	1.81	1.50	49.3	107.5	0.54	0.18	84.1%	4.01	1.5
Self_70	1.00	2.33	1.30	45.4	88.7	0.50	0.15	69.9%	2.99	1.7
Self_71	1.19	1.69	0.79	47.8	107.1	0.50	0.46	86.8%	3.08	2.0
Self_72	1.38	2.40	1.09	47.7	87.1	0.58	0.58	74.8%	4.00	1.2
Self_73	0.76	2.40	1.50	50.0	127.3	0.50	0.49	81.1%	4.32	1.3
Self_74	0.82	2.39	1.45	47.0	123.8	0.51	0.50	84.7%	4.75	1.7
Self_75	0.90	2.40	1.44	45.2	128.7	0.85	0.55	91.2%	3.48	1.7
Self_76	1.18	1.94	1.50	46.0	90.3	0.50	0.24	88.0%	4.20	1.7
Self_77	1.14	1.77	1.37	35.9	124.8	0.60	0.46	89.1%	3.74	1.5
Self_78	1.12	2.07	1.46	30.7	105.7	0.50	0.01	78.6%	4.36	1.4
Self_79	0.81	2.40	1.45	34.0	109.1	0.68	0.08	95.2%	4.20	1.7
Self_80	1.32	2.34	1.47	42.3	108.9	0.50	0.03	94.3%	4.35	1.6
Self_81	1.15	2.28	1.45	49.4	129.9	0.50	0.00	92.6%	1.75	1.6
Self_82	1.07	2.36	1.50	49.1	52.3	0.55	0.03	89.5%	5.42	2.0
Self_83	1.32	2.37	1.28	41.5	113.8	0.69	0.58	78.5%	3.04	1.3
Self_84	0.83	2.28	1.43	19.4	86.5	0.50	0.08	57.3%	3.06	1.0
Self_85	0.56	2.27	1.46	19.1	125.8	0.50	0.00	78.4%	3.45	1.2

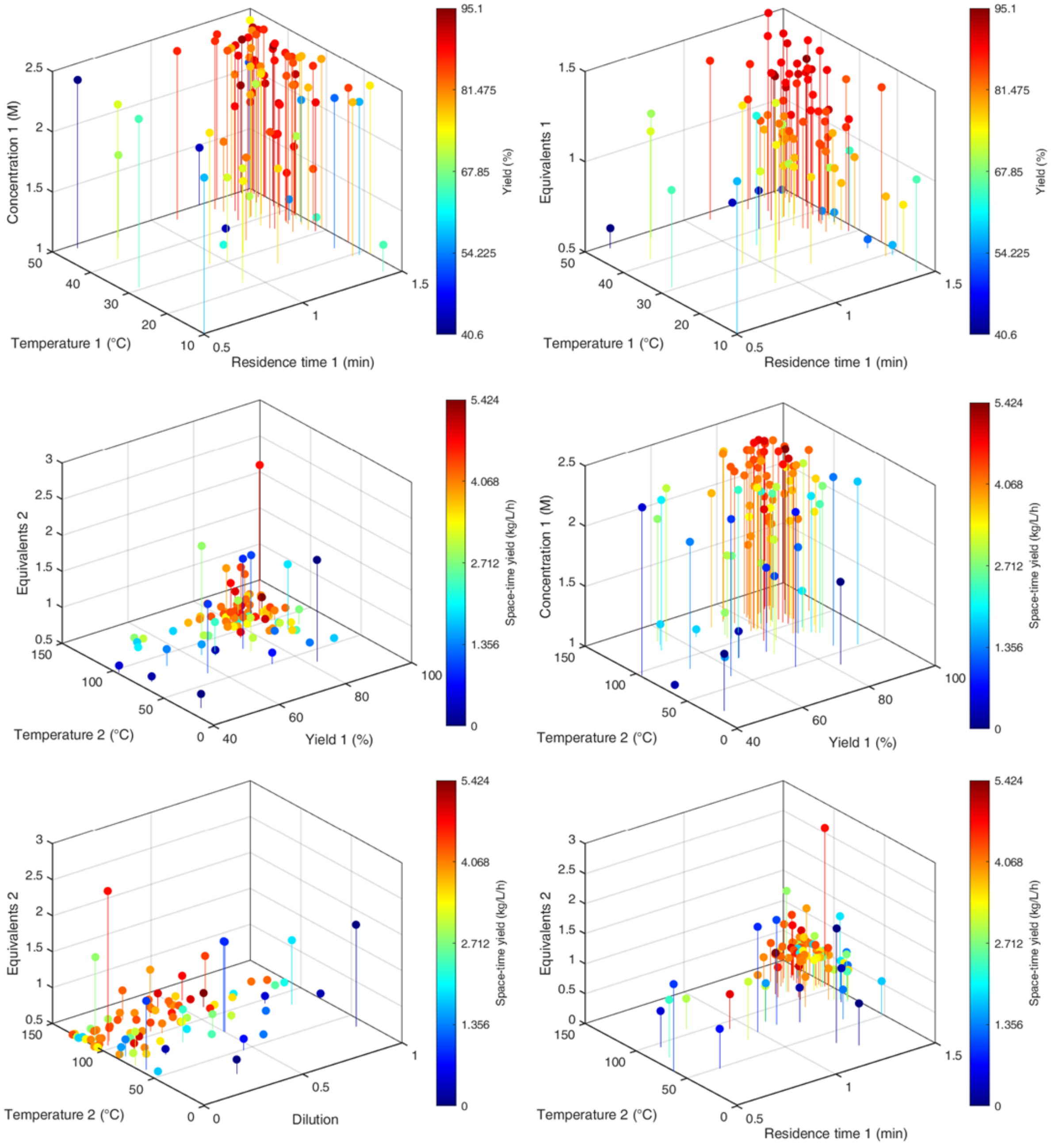

Figure S41. Optimization plots for the edaravone synthesis center points as initial data set.

7 NMR Spectra

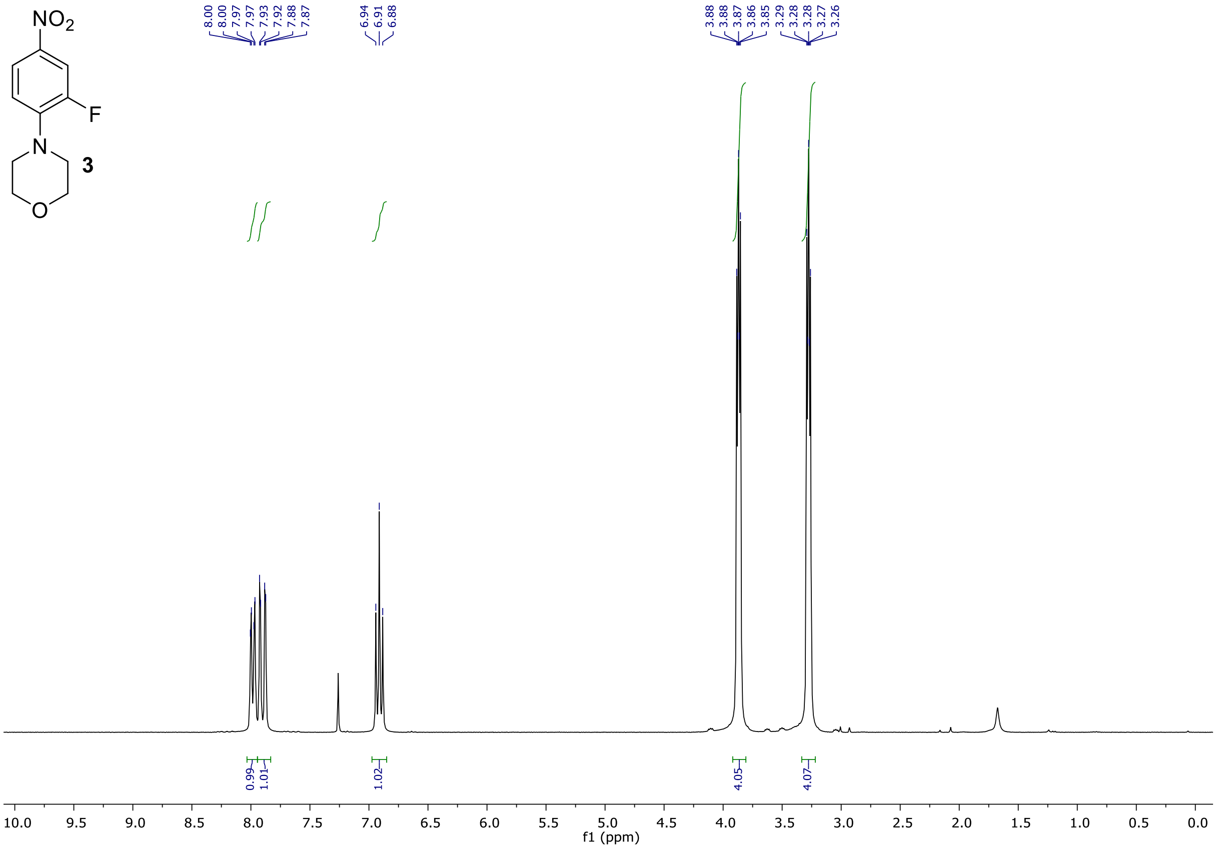

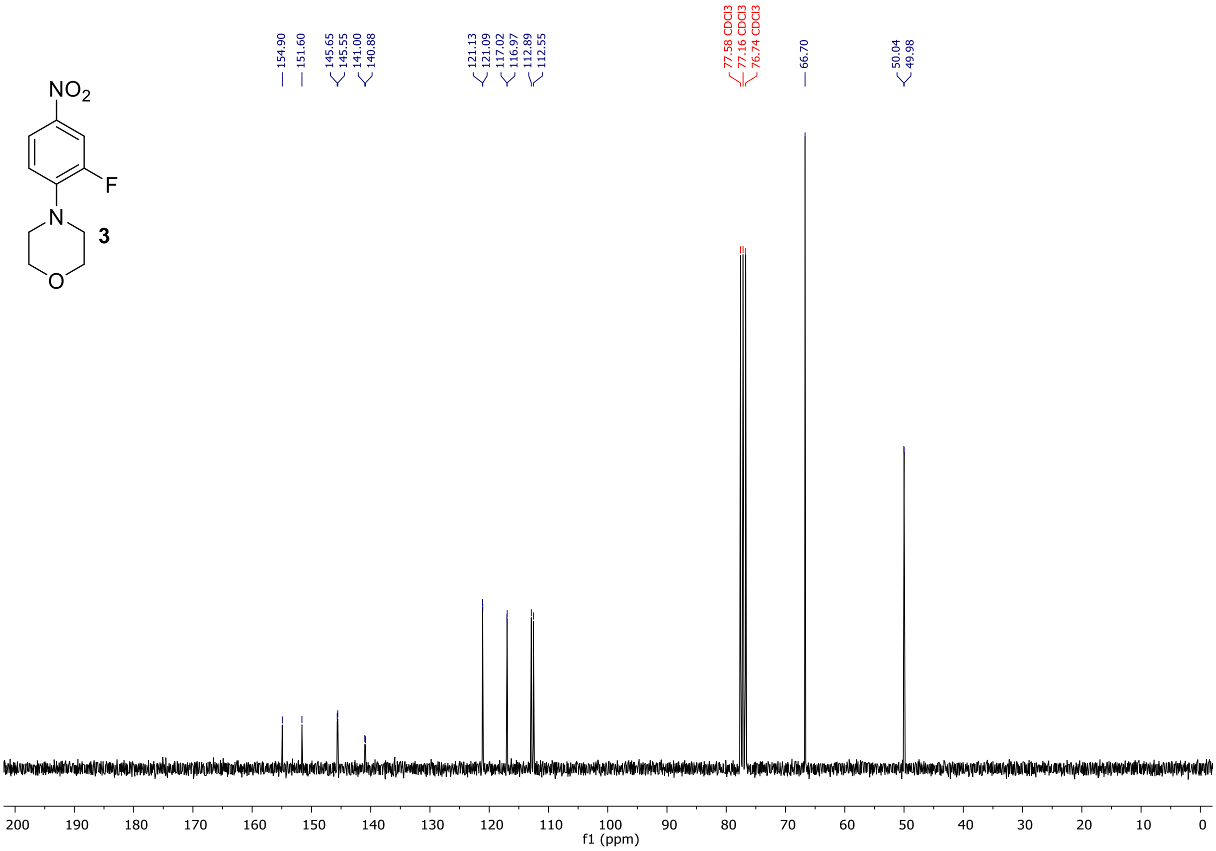

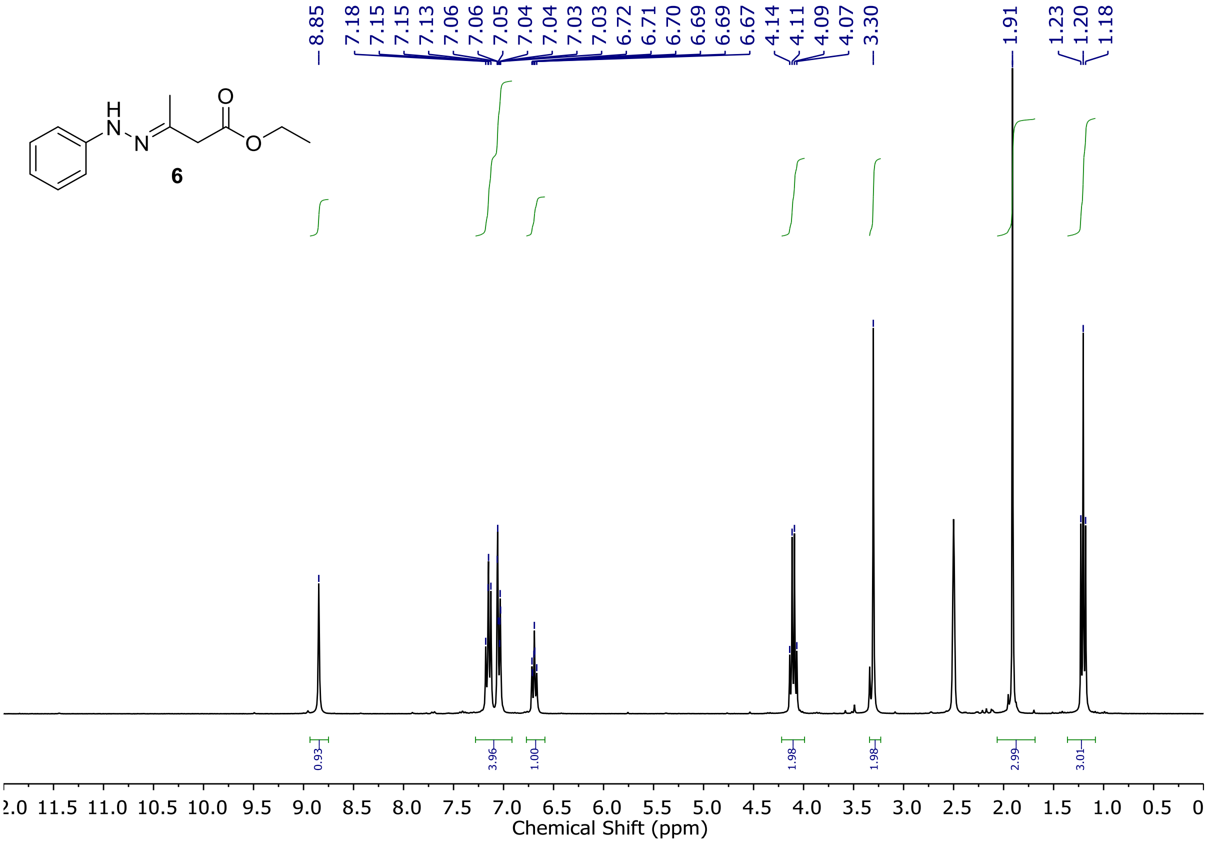
8.85
7.18
7.15
7.15
7.13
7.06
7.06
7.05
7.04
7.04
7.03
7.03
6.72
6.71
6.70
6.69
6.69
6.67
4.14
4.11
4.09
4.07
3.30
1.91
1.23
1.20
1.18
6
0.93
3.96
1.00
1.98
1.98
2.99
3.01
Chemical Shift (ppm)

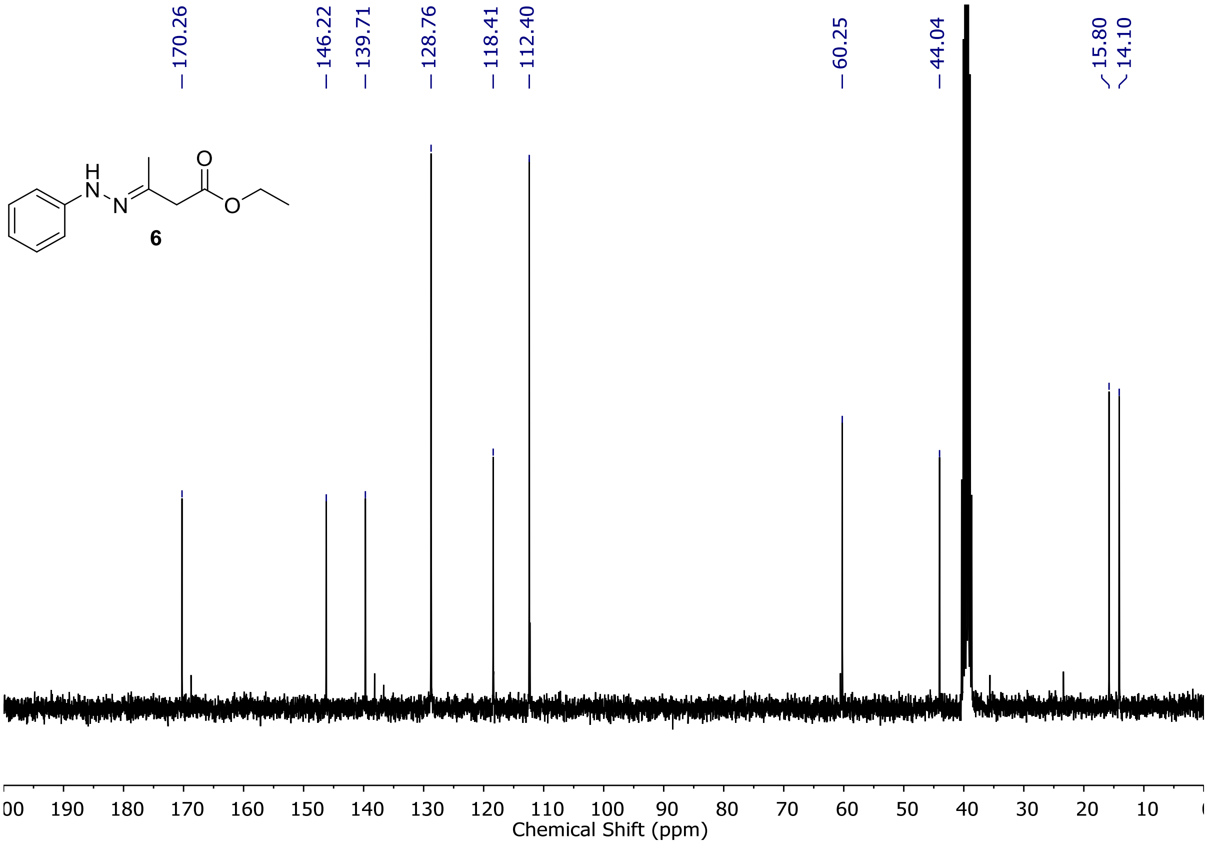
170.26
146.22
139.71
128.76
118.41
112.40
60.25
44.04
15.80
14.10
6
Chemical Shift (ppm)

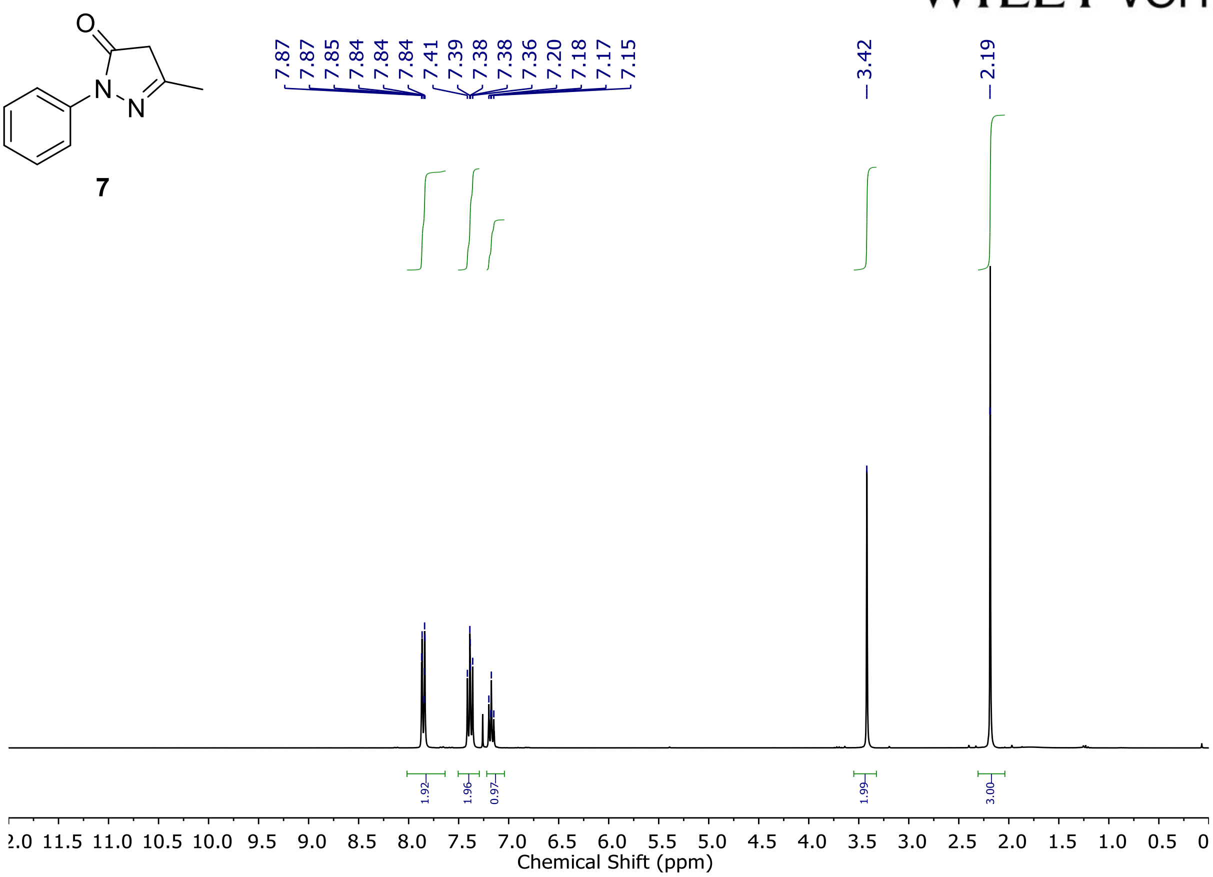
O
N
N
7
7.87
7.87
7.85
7.84
7.84
7.84
7.41
7.39
7.38
7.38
7.36
7.20
7.18
7.17
7.15
3.42
2.19
1.92
1.96
0.97
1.99
3.00
12.0 11.5 11.0 10.5 10.0 9.5 9.0 8.5 8.0 7.5 7.0 6.5 6.0 5.5 5.0 4.5 4.0 3.5 3.0 2.5 2.0 1.5 1.0 0.5 0
Chemical Shift (ppm)

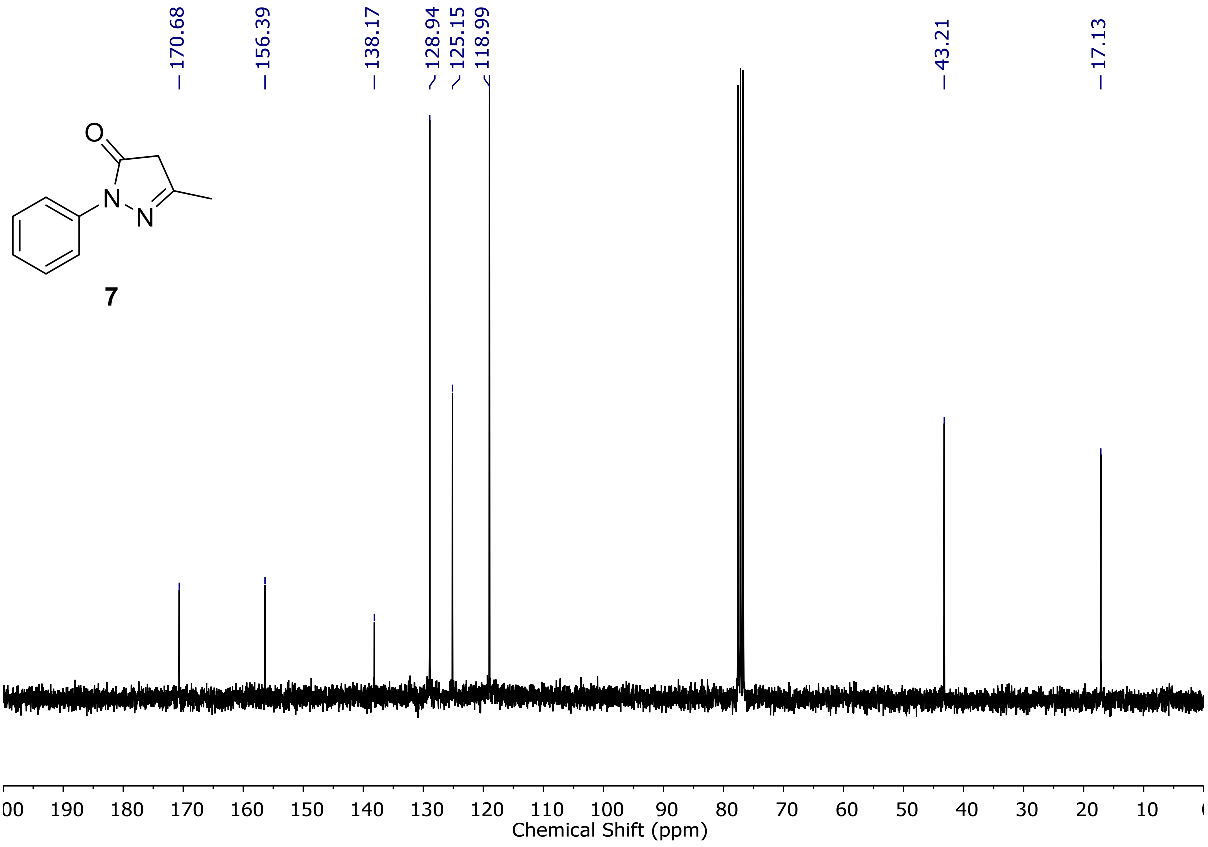
170.68
156.39
138.17
128.94
125.15
118.99
43.21
17.13
O
N
N
7
200 190 180 170 160 150 140 130 120 110 100 90 80 70 60 50 40 30 20 10 0
Chemical Shift (ppm)

8 References

[1] A. M. Schweidtmann, A. D. Clayton, N. Holmes, E. Bradford, R. A. Bourne, A. A. Lapkin, *Chem. Eng. J.* **2018**, *352*, 277–282.

[2] A. K. Tewari, V. P. Singh, P. Yadav, G. Gupta, A. Singh, R. K. Goel, P. Shinde, C. G. Mohan, *Bioorg. Chem.* **2014**, *56*, 8–15.
